# Development and Validation of an Ultra-Performance Liquid Chromatography Method for the Determination of Wedelolactone in Rat Plasma and its Application in a Pharmacokinetic Study

**DOI:** 10.3390/molecules24040762

**Published:** 2019-02-20

**Authors:** Qing Chen, Xiaoxue Wu, Xuemin Gao, Hua Song, Xuan Zhu

**Affiliations:** Fujian Provincial Key Laboratory of Innovative Drug Target Research, School of Pharmaceutical Sciences, Xiamen University, Xiamen 361002, China; chenqing@xmu.edu.cn (Q.C.); xiaoxue_wu1@126.com (X.W.); holygxm@xmu.edu.cn (X.G.)

**Keywords:** wedelolactone, pharmacokinetics, UPLC, rat plasma

## Abstract

Wedelolactone is a coumarin ether with significant hepatoprotective effects. However, there are few pharmacokinetic studies of wedelolactone, which will affect the studies of its efficacy and potential toxicity. In this study, a selective ultra-performance liquid chromatography (UPLC) method was developed to confirm the pharmacokinetic parameters of wedelolactone in rat plasma. The chromatographic separation was carried out on a Kromasil C_18_ UPLC column (250 × 4.6 mm; 5.0 μm) by gradient mobile phase of methanol-water containing 0.5% acetic acid (*v*/*v*). Perfect linearity was obtained and the samples were stable under different conditions. The intra-day and inter-day precisions (relative standard deviation, %) were within 3.81% and accuracies (relative error, %) ranged from −4.01% to 7.12%. The extraction recoveries in rat plasma ranged from 95.98% to 108.93%. This rapid method was successfully applied in the pharmacokinetic study of wedelolactone in rat plasma. Following the oral administration of 5.00 mg/kg wedelolactone, the wedelolactone was rapidly absorbed. Pharmacokinetic parameters were used to quantitatively describe the dynamic changes of wedelolactone in vivo, providing a theoretical basis for pharmacological research on drugs and preclinical medication. The study of wedelolactone can provide a theoretical basis and quick analysis for the study of other traditional Chinese medicine. This may lead to breakthroughs in the pharmacokinetic study of complex Chinese medicines.

## 1. Introduction

Wedelolactone (7-methoxy-5,11,12-trihydroxycoumestan) is an active compound of coumarin ether [1], which is isolated from *Wedelia prostrata*, *Herba ecliptae*, *Hypericum erectum*, and other traditional Chinese medicines (TCM) [2,3]. In recent years, many pharmacological studies have shown that wedelolactone exhibits a wide range of biological activities, including hepatoprotective [4], antiosteoporosis [5], enzyme inhibition [6,7], antiphlogistic [8], anti-inflammatory [9], promoting cell proliferation [10], and other effects [11,12,13]. Wedelolactone has been found to be useful in the treatment of osteoporosis, pulmonary embolism, and ulcerative colitis. It is also the main constituent of Erzhi Pills [14], which are a Chinese patent drug and used to tonify the liver and kidney. Erzhi Pills are used for the treatment of the deficiency of giddiness, tinnitus, and waist and knee pain, with good sales. Nevertheless, there are few pharmacokinetic studies of wedelolactone, including its absorption, disposition, metabolism, and excretion in vivo, which have significantly impacted studies of its efficacy and potential toxicity. Nowadays, the main methods of determination and pharmacokinetics for wedelolactone are HPLC and LC-MS [15,16,17]. Because wedelolactone is isolated from *Eclipta prostrata*, the pharmacokinetic studies of the extract in *Eclipta prostrata* were investigated in some reports [16,17]. There is not a single article which has reported the pharmacokinetic studies of wedelolactone. Additionally, pharmacokinetic parameters of wedelolactone reported in different studies vary greatly.

The UPLC technique is the main analytical technique used in drug research. It is widely applied to pharmacokinetic and metabolite identification in vitro or in vivo. At present, the content determination of wedelolactone has been reported, but is mainly concentrated in the HPLC method. Compared with traditional HPLC, UPLC has a speed of nine times, a sensitivity of three times, and a separation of 1.7 times. Therefore, UPLC should be applied in the analysis of new fields, such as pharmacokinetic and biochemical analysis. Because most samples are very complex, the number of chromatographic peaks that can be separated is not enough. Under the same conditions, UPLC is better than HPLC, which can separate more than twice the chromatographic peaks, so it is possible to study them further. In this study, a selective ultra-performance liquid chromatography (UPLC) method was developed for the determination of wedelolactone in rat plasma and its application in a pharmacokinetic study following oral administration. Furthermore, pharmacokinetic studies revealed quantitative dynamic changes of wedelolactone in vivo, which offered a scientific basis for the research and development of wedelolactone. The detailed pharmacokinetic parameters were important for further research on wedelolactone, making the application of liver protecting drugs with wedelolactone more safe and efficient in clinical treatment.

## 2. Results and Discussion

### 2.1. Method Development

Appropriate chromatographic behaviors, such as improved resolution, good peak symmetry, high sensitivity of detection, and different categories of column and mobile phases, are important for the analysis of wedelolactone. Compared with other columns, the Kromasil C_18_ UPLC column (250 × 4.6 mm; 5.0 μm) revealed improved peak shapes and an appropriate retention time for analytes and was selected for analysis to obtain better chromatographic separation. Favorable chromatographic behavior is based on the proper mobile phase. Methanol as the organic phase could show a higher response and lower background noise compared with acetonitrile. The gradient mobile phase also showed good peak symmetry and was stable for analysis. Moreover, the peak shapes were improved and the retention time was stable because of the addition of 0.5% acetic acid in water and methanol. A gradient elution at a flow rate of 1.0 mL/min was selected for determination of the analytes and IS.

A good extraction method is essential for quantitative analysis. The protein precipitation method with organic solvent has a good accuracy and solvents can be easily removed and recovered. In this study, the protein precipitation method was selected to determine the method with the best sensitivity and recovery of analytes. With the protein precipitation method, methanol showed a strong response value and clear supernatant for the analytes compared with other protein-precipitating reagents. In other words, protein precipitation with methanol was an efficient technique for the extraction of wedelolactone. As a result, simple and inexpensive precipitation of the sample was selected to be the most suitable method.

### 2.2. Method Validation

**Figure 1:**
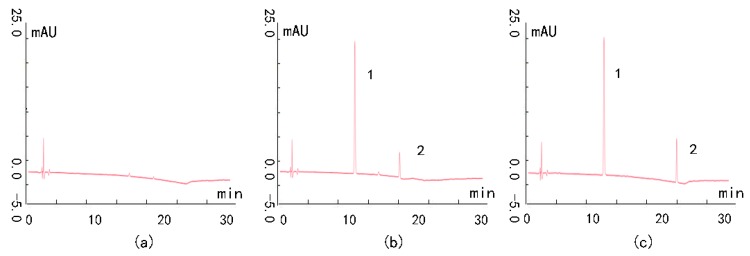
Selectivity of the ultra-performance liquid chromatography (UPLC) method. Peaks: **1** scoparone, **2** wedelolactone. Conditions: column, Kromasil C_18_ UPLC column (4.6 × 250 mm; 5.0 μm); solvent system, solvent A (methanol) and solvent B (0.5% acetic acid in water), gradient elution: 0–10 min, 35–55% A; 10-20 min, 55–80% A; 20–25 min, 80% A; flow rate, 1.0 mL/min; detection wavelength, 351 nm. (**a**) Blank plasma; (**b**) Blank plasma spiked with the wedelolactone and IS; (**c**) Rat plasma sample.

The chromatograms of blank plasma, blank plasma spiked with wedelolactone and scoparone, and rat plasma testing samples are shown in Figure 1. Wedelolactone and the IS were eluted at retention times of 12 and 18 min, respectively. No significant interfering endogenous peaks were observed in rat plasma at the retention times of the analytes and the IS under these chromatographic conditions. This result indicated that plasma samples could be accurately differentiated and quantified with this method.

The final concentrations of the standard solutions were 1.9375, 3.875, 7.75, 15.5, 31, 62, and 124 μg/mL. The linearity of the calibration curve was determined by plotting the peak area ratios (wedelolactone/IS) versus the concentrations of analytes. The linear equation of the calibration curve obtained was: *y* = 0.0394*x* − 0.0221 (r^2^ = 0.999) in a concentration range of 1.9375–124 μg/mL. The lower limit of quantification of wedelolactone was determined to be 1.9375 μg/mL, with a signal noise ratio greater than 10, which met the standard for pharmacokinetic studies in rats.

Intra-day precisions, inter-day precisions, and accuracies at the three concentration levels are summarized in Table 1. The intra- and inter-day precisions (RSD, %) were less than 3.84%, and thus within the acceptable criteria. The accuracies (RE, %) of low, medium, and high concentrations of the QCs of the analytes ranged from −4.01% to 7.12%. All of the data indicated that this method was reliable, accurate, and reproducible for the study of wedelolactone.

The extraction recoveries of wedelolactone from rat plasma are shown in Table 2. The extraction recoveries in rat plasma of the three concentrations ranged from 95.98 to 108.93%, which was within the acceptable range. The results revealed that the selected method was dependable.

### 2.3. Stability

Table 3 summarizes the stability of the analytes under different conditions. Following incubation at room temperature, a significant decrease was observed (RE values ranging from −43.91% to −15.63%), indicating that wedelolactone was not stable in plasma at room temperature. The samples could be placed at 4 °C for 24 h (RE values ranging from −3.03% to 6.27%) and RE values of the analytes placed at −20 °C for 15 d were between −4.18% and 6.19%. The results indicated that wedelolactone was stable at low temperature, and that the wedelolactone samples could be analyzed under the low temperature conditions in the sample preparation. Consequently, this method was suitable for the pharmacokinetic study of wedelolactone.

### 2.4. Pharmacokinetic Analysis

**Figure 2:**
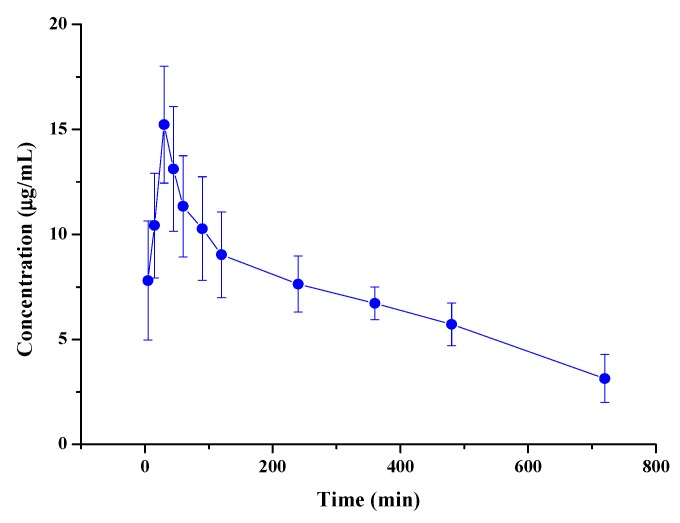
Mean plasma concentration vs. time profiles following oral (5.00 mg/kg) administration of wedelolactone in rats (n = 6).

This method was successfully employed for the pharmacokinetic analysis of wedelolactone in rats. Figure 2 illustrates the mean plasma concentration versus time profiles of the analytes. The mean pharmacokinetic parameters from non-compartment modeling are shown in Table 4. Control group results showed no effect for rats. Following the oral administration of 5.00 mg/kg wedelolactone, the concentration reached the maximum at 30 min, suggesting that wedelolactone was rapidly absorbed through the gastrointestinal tract. However, the C_max_ was only 15.22 mg/L. Rapid decline of the plasma drug concentration after T_max_ could be partly due to rapid tissue distribution (CL, 0.06 L/h and V, 0.39 L/kg). The AUC_0–t_ was 83.05 mg/L × h. The mean residence time of the analytes was 4.76 h and the T_1/2_ was 4.51 h.

### 2.5. Metabolism In Vitro

**Figure 3:**
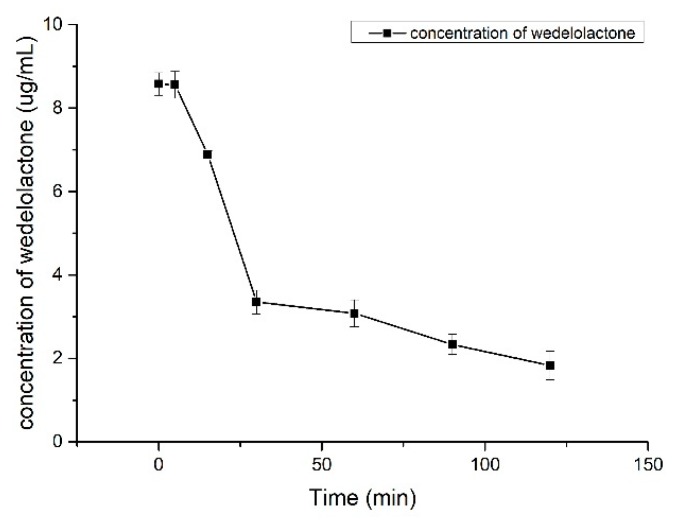
Metabolism in vitro of wedelolactone in liver microsomes (n = 3).

In the negative control group, wedelolactone did not undergo transformation. Figure 3 shows the in vitro metabolism of wedelolactone in liver microsomes under the catalysis of the NADPH enzyme. It can be seen that after incubating for 120 min, the metabolic rate of wedelolactone was 78.70%, which showed that most of the wedelolactone was metabolized in vitro. By comparing the metabolic curves of wedelolactone in vivo and in vitro, the same metabolic rule of wedelolactone in vivo and in vitro was presented. The CYP enzyme is an important part of the drug metabolic enzyme system in liver microsomes and the intestine. Under the catalytic action, wedelolactone rapidly metabolized and declined, which showed the non-specific regularity of metabolism.

In this paper, a UPLC method for the detection of wedelolactone was established. The method showed strong specificity, high sensitivity, and good stability, which met the detection requirements of biological samples. Under this chromatographic condition, wedelolactone and internal standard scoparone exhibited better separation, a shorter peak time, and endogenous substances in plasma. The gradient elution method can obviously improve the peak shape of wedelolactone and scoparone, which reached the requirements of detection. The method of methanol precipitation was used in this experiment, and it was easy to operate and can remove proteins from plasma to a great extent with a high recovery rate.

At present, the content determination of wedelolactone had been reported, but is mainly concentrated in the HPLC method. However, research on the UPLC method is less frequently reported. The established method of UPLC in this study can not only be used to detect wedelolactone efficiently, but also be used to determine the metabolic constants of drugs in vivo rapidly and accurately. This method provided important technical support for the further study of wedelolactone. The results showed that following the oral administration of 5.00 mg/kg wedelolactone, the concentration reached the maximum at 30 min, suggesting that wedelolactone was rapidly absorbed through the gastrointestinal tract. However, the C_max_ was only 15.22 mg/L. It is suggested that oral administration should not be used in pharmacological and pharmacodynamic experiments, so as to avoid first-pass elimination. This result can be used as an important basis for clinical medication.

## 3. Materials and Methods

### 3.1. Chemicals and Regents

Wedelolactone (purity > 98%) was purchased from Nanjing Jingzhu Bio Technology Co., Ltd. (Nanjing, China). Scoparone (purity > 98%) was purchased from Nanjing Guangrun Bio Technology Co., Ltd. (Nanjing, China). Methanol and acetic acid were all of HPLC grade and purchased from Xiamen Kezhan Co., Ltd. (Xiamen, China). Water used in this study was generated by a water purification system (RSJ Scientific Instruments Co., Xiamen, China). For method validation, plasma samples from nine male Sprague-Dawley rats (160–220 g, Laboratory Animal Center of Xiamen University, Xiamen, China) were obtained with heparin sodium and blank plasma samples were collected by centrifugation at 3000 rpm for 10 min at 4 °C. Plasma samples were stored at −20 °C before use. Male rat pooled liver microsomes (Sprague-Dawley) was purchased from Shanghai Yayu Biotechnology Co., Ltd. (Shanghai, China) and stored at −80 °C before use. The NADPH coenzyme system was purchased from Beijing Solarbio Technology Co., Ltd. (Beijing, China) and was stored at −20 °C. PBS buffer solution was purchased from GE Healthcare Life Science Co., Ltd. (Boston, MA, USA).

**Figure 4:**
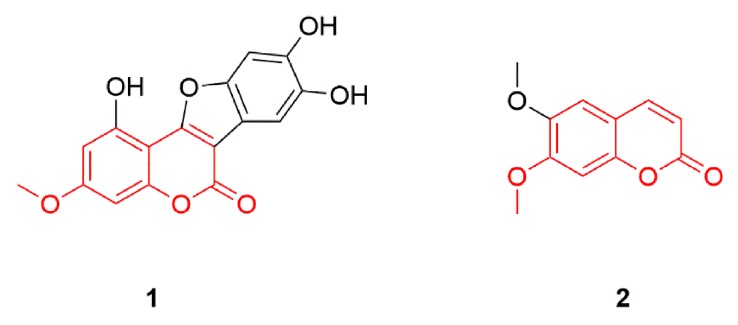
Chemical structure of wedelolactone and scoparone. Compound **1**: wedelolactone; **2**: scoparone.

Due to the structural similarity of scoparone and wedelolactone (Figure 4), scoparone was chosen as the internal standard (IS). Stock solutions of wedelolactone and the IS were prepared with methanol and used for the preparation of the calibration standards and quality control (QC) samples, which were stored at 4 °C until use.

### 3.2. Conditions of Instruments

The ultra-performance liquid chromatography (UPLC) system consisted of a Kromasil C_18_ UPLC column (250 × 4.6 mm, 5.0 μm, Thermo Fisher Scientific, Waltham, MA, USA), a quaternionic pump (Thermo Fisher Scientific, USA), an autosampler (Thermo Fisher Scientific, USA), and a UV detector (PDS-M20A, Thermo Fisher Scientific, USA). The mobile phase consisted of solvent A (methanol) and solvent B (0.5% acetic acid in water) at a flow rate of 1.0 mL/min. All solvents were filtered through a 0.45 μm membrane. The gradient elution process was carried out as follows: 0–10 min, 35–55% A; 10–20 min, 55–80% A; 20–25 min, 80% A. The detection wavelength was set at 351 nm.

### 3.3. Working Solutions

Stock solutions of wedelolactone and IS were prepared in methanol at concentrations of 1240 and 580 μg/mL, respectively. Working solutions ranging from 1.9375 to 124 μg/mL for control and standard samples were generated by dilution of the stock solution with methanol. The stock solution of IS was diluted with methanol to a concentration of 58 μg/mL, which was considered the working solution of IS. Unextracted solutions containing 1.9375, 7.75, and 31 μg/mL of wedelolactone and 58 μg/mL of IS were prepared with methanol. The solution of wedelolactone for metabolism in vitro was prepared at a concentration of 1 mg/mL with methanol. All solutions were stored at 4 °C before use.

### 3.4. Sample Processing

To stay stable before addition into plasma, the rat plasma samples, standards, and QC samples were thawed and placed at a low temperature. A total of 100 μL working solution of IS (58 μg/mL) was added to 100 μL of plasma samples in a 1.5 mL centrifuge tube. The sample was vortexed for 30 s and 200 μL of methanol was added. The mixture was vortexed again for 30 s and centrifuged at 12,000 rpm for 10 min at 4 °C. The supernatant (200 μL) was collected into a new tube. In the last step, 20 μL of the supernatant was injected into the UPLC system for analysis.

### 3.5. Preparation of Standards and QC Samples

The standard solutions were prepared by spiking working solutions into 100 μL of blank rat plasma. The final concentrations of wedelolactone ranged from 1.9375 to 124 μg/mL (1.9375, 3.875, 7.75, 15.5, 31, 62, and 124 μg/mL). The QC samples were obtained at concentrations of 1.9375, 7.75, and 31 μg/mL (low, middle, and high), which was similar to the standards. All samples were stored at −20 °C. The standard and QC samples were used to estimate the precision and accuracy of the method.

### 3.6. Method Validation

Method validation was performed according to the “Guidance for Industry Bioanalytical Method Validation”, which was recommended by the Food and Drug Administration and the European Medicines Agency [18,19].

Selectivity of the method was assessed by comparing the chromatograms of blank plasma, blank plasma spiked with wedelolactone and scoparone, and rat plasma samples (see Supplementary Materials). Three different batches were tested following the same procedure. This experiment was repeated five times.

The standard samples, ranging from 1.9375 to 124 μg/mL, were tested to determine the calibration curve. The linearity of calibration curves was determined by plotting the peak area ratios (analytes/IS) against the analyte concentrations and the data was well fitted by least-square regression with the weighting factor of 1/x^2^ in the concentration range.

The intra-day accuracies and precisions were evaluated by determining five replicated QC samples at three different concentrations: 1.9375 (low), 7.75 (medium), and 31 μg/mL (high), within one day. The inter-day accuracies and precisions were assessed by analyzing the samples five times at three days at the three different concentrations. Concentrations were calculated with the obtained calibration curves. The relative standard deviation (RSD) was used to evaluate the precision and the accuracy was expressed by relative error (RE).

The extraction recoveries of wedelolactone were measured by the comparison of the peak area of QC samples with those of post-extracted spiked samples at three concentrations (1.9375, 7.75, and 31 μg/mL; n = 5).

### 3.7. Stability

Under different conditions, wedelolactone in rat plasma was investigated five times using three different concentrations (1.9375, 7.75, and 31 μg/mL; low, middle, and high, respectively) of QC samples. The stability of wedelolactone was evaluated by assessing the exposure of the spiked samples 1) at 4 °C for 24 h, 2) at −20 °C for 15 d, and 3) at room temperature for 4 h.

### 3.8. Pharmacokinetic Analysis

The pharmacokinetics of wedelolactone were investigated in rats following oral administration. All animal protocols were approved according to the guiding principle of the Xiamen University Institutional Animal Care and Use Committee. Male Sprague-Dawley rats (160–220 g) were used in this study. Rats were housed in a room under standard environmental conditions (12 h light and 12 h dark cycle) for a few days before the experiment. Standard animal food and water were provided ad libitum. Before the experiment, diet was prohibited for 12 h while water was continuously provided.

Wedelolactone was dissolved in normal saline for the preparation of dosing solutions. Six rats were given dosing solutions by oral administration at a dose of 5.00 mg/kg and three rats were given the same doses for the control group. Following anaesthetization using ether, 0.5 mL blood samples were collected in 1.5 mL heparinized centrifuge tubes at 5, 15, 30, 45, 60, 90, 120, 240, 360, 480, and 720 min. Blood samples were placed on ice and centrifuged immediately (3000 rpm, 4 °C, 10 min). The plasma samples were collected and stored at −20 °C until analysis.

### 3.9. Metabolism In Vitro

Male rat pooled liver microsomes (Sprague-Dawley 20 mg/mL) were diluted into suspension with a concentration of 1.0 mg/mL by PBS buffer solution. A total of 100 μL of wedelolactone solution was added to 1.0 mL suspension and mixed. This mixture was preincubated for 3 min in a 35 °C shaker. Then, assays were initiated by the addition of the solution of NADPH to the mixed solution, with a final concentration of 0.1 mmol/L. After incubation for 0, 5, 15, 30, 60, 90, and 120 min at 35 °C, the reaction was stopped by adding 2 mL methanol. The mixture was vortexed for 2 min, centrifuged at 2500 rpm for 10 min. The supernatant was dissolved in 2 ml of mobile phase and was prepared for HPLC analysis. Each incubation was conducted in duplicate using the same pools of microsomes (n = 3).

### 3.10. Statistical Analysis

The plasma concentration of wedelolactone was expressed as the mean ± standard deviation, and the curve of mean concentration–time was plotted. Data fitting and pharmacokinetic parameters were based on non-compartmental analysis using WinNonLin Software (version 6.1; Pharsight Corporation, St. Louis, MO, USA)

## 4. Conclusions

The present study described the pharmacokinetic parameters of wedelolactone with significant pharmacological activity, particularly hepatoprotective effects. The UPLC method was first applied for the determination of wedelolactone in rat plasma. Through the same rule of metabolism in vivo and in vitro, the relationship between the efficacy and dose of wedelolactone can be understood easily through the analysis of Pharmacokinetic/Pharmacodynamics (PK/PD) and the study of PK/PD will be helpful for the clinical study of multiple sclerosis.

Additional studies will be performed to further investigate the pharmacokinetic parameters of wedelolactone in rat plasma following intravenous administration and to determine the absolute oral bioavailability. Determination of the pharmacokinetic parameters by different routes of administration will also be necessary. Understanding the metabolism of wedelolactone will be important, which will provide a theoretical basis for the pharmacological research on drugs. First pass metabolism can be overcome through selecting a proper route of administration and analysis of different pharmacokinetic parameters of wedelolactone.

Wedelolactone is the main component of many Chinese medicines, including *Wedelia prostrata*, *Herba ecliptae*, and *Hypericum erectum.* With the development of clinical pharmacies in China, study of the pharmacokinetics of Chinese medicines has attracted more and more attention. The study of wedelolactone can act as guidance for the study of other TCM. The UPLC method was applied for the determination and pharmacokinetic study of wedelolactone in rat plasma, which was a quick, simple, and accurate method. Therefore, this method may be widely used in the determination of many compounds. Today, although some achievements in the pharmacokinetic study of TCM have been achieved, there is still a lot of work to do. This work described a simple UPLC method looking at not only wedelolactone, but also the metabolic products peak found in vitro and in vivo. Metabolic pathways and metabolites can also be further studied. Wedelolactone is only a single compound, but in TCM, complex components and interactions between them will affect the metabolism of drugs in vivo. Therefore, the next research direction can be changed from single to multiple compounds and should consider investigating multiple compounds. The pharmacokinetics of various and effective ingredients in TCM should be analyzed and new technologies and new theories like UPLC and mass spectrometry can be used in pharmacokinetic studies. This may lead to breakthroughs in the pharmacokinetic study of complex Chinese medicines.

## Figures and Tables

**Table 1:** Precision and accuracy of the method for wedelolactone in rat plasma (n = 5).

Concentration (μg·mL^−1^)	Intra-Day		Inter-Day	
	RSD (%)	RE (%)	RSD (%)	RE (%)
1.9375	3.79	5.72	3.72	7.12
7.75	1.88	−2.41	2.53	−3.15
31	2.56	−2.73	3.84	−4.01

**Table 2:** Extraction recovery of wedelolactone in rat plasma (n = 5).

Concentration (μg·mL^−1^)	Extraction Recovery (%)
1.9375	98.11 ± 2.23
7.75	100.54 ± 5.43
31	102.26 ± 6.73

**Table 3:** Stability of wedelolactone in different conditions in rat plasma (n = 5).

Concentration (μg·mL^−1^)	4 °C for 24 h		−20 °C for 15 d		RT for 4 h	
	RSD (%)	RE (%)	RSD (%)	RE (%)	RSD (%)	RE (%)
1.9375	1.12	6.27	2.97	6.19	10.94	−40.44
7.75	1.35	−2.09	1.82	−2.01	5.65	−43.91
31.00	2.21	−3.03	1.94	−4.18	7.45	−15.63

**Table 4:** Mean pharmacokinetic parameters of wedelolactone (n = 6).

Parameters	Unit	Oral (5.00 mg/kg)
		Mean
T_max_	h	0.50
C_max_	mg/L	15.22
T_1/2_	h	4.51
AUC_0−∞_	mg/L × h	83.05
CL/F	L/h	0.06
V/F	L/kg	0.39
MRT	h	4.76

T_max_, peak time; C_max_, maximum concentration; T_1/2_, half-life; AUC_0-t_, area under the curve; CL/F, clearance; V/F, apparent volume of distribution; MRT, mean residence time.

## Supplementary Materials

The following UPLC chromatograms of methodological validation and metabolism in six rats are available as supporting data. Supplementary materials are available.
