# Supplementary material for: Development and Validation of an Ultra-Performance Liquid Chromatography Method for the Determination of Wedelolactone in Rat Plasma and its Application in a Pharmacokinetic Study
Source: Molecules. 2019 Feb 20;24(4):762. doi: 10.3390/molecules24040762 (PMC6413069; doi:10.3390/molecules24040762)
Supplement: Supplementary file 1 [file molecules-24-00762-s001.pdf]

Supplementary Materials

Development and Validation of an Ultra-Performance Liquid Chromatography Method for the Determination of Wedelolactone in Rat Plasma and its Application in a Pharmacokinetic Study

Qing Chen †, Xiaoxue Wu †, Xuemin Gao, Hua Song * and Xuan Zhu *

Fujian Provincial Key Laboratory of Innovative Drug Target Research, School of Pharmaceutical Sciences, Xiamen University, Xiamen 361002, China; chenqing@xmu.edu.cn (Q.C.); xiaoxue_wu1@126.com (X.W.); holygxm@xmu.edu.cn (X.G.)

* Correspondence: songhua@xmu.edu.cn (H.S.); zhuxuan@xmu.edu.cn (X.Z.); Tel.: +0592-2881181 (X.Z.)

† These authors contributed equally to this paper.

Selectivity

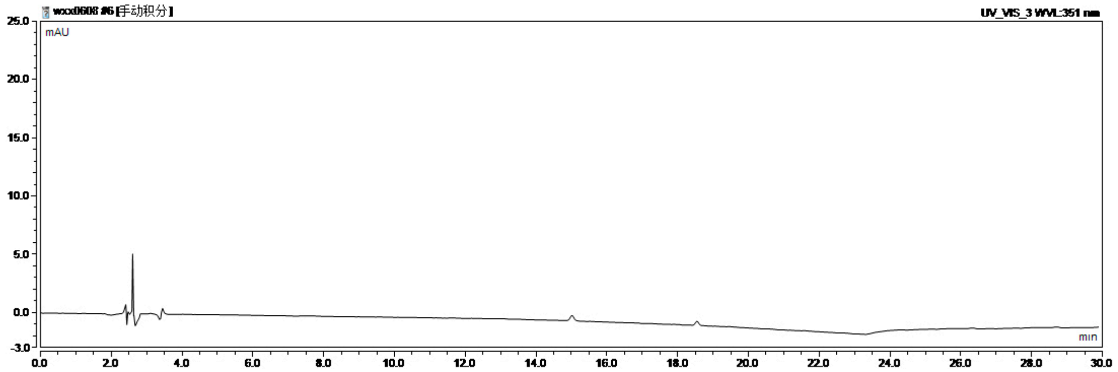

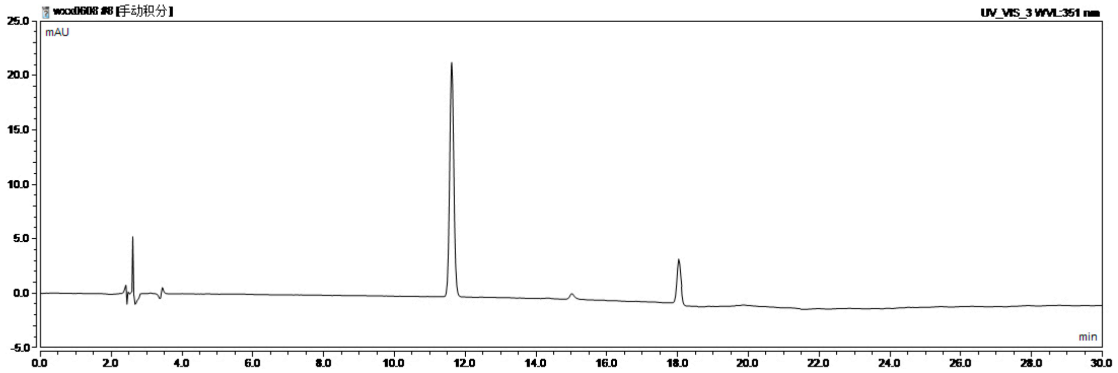

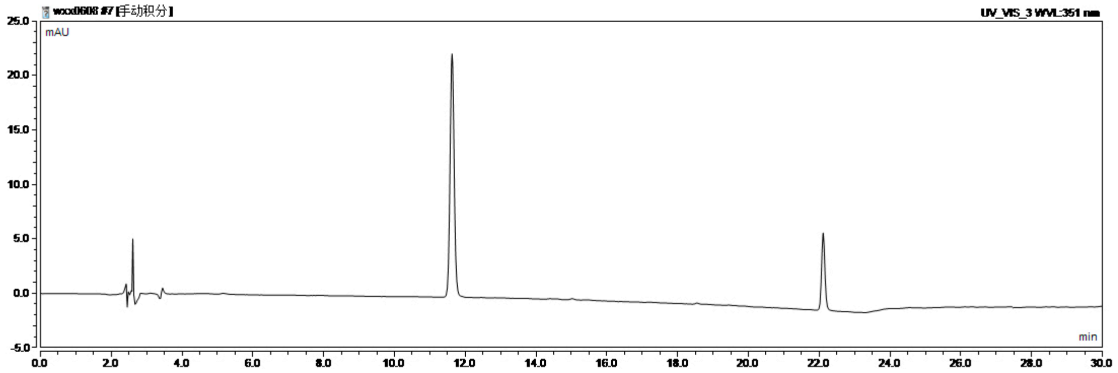
wxx0608 #7 [手动积分]
UV_VIS_3 WVL:351 nm
mAU
min

Linear

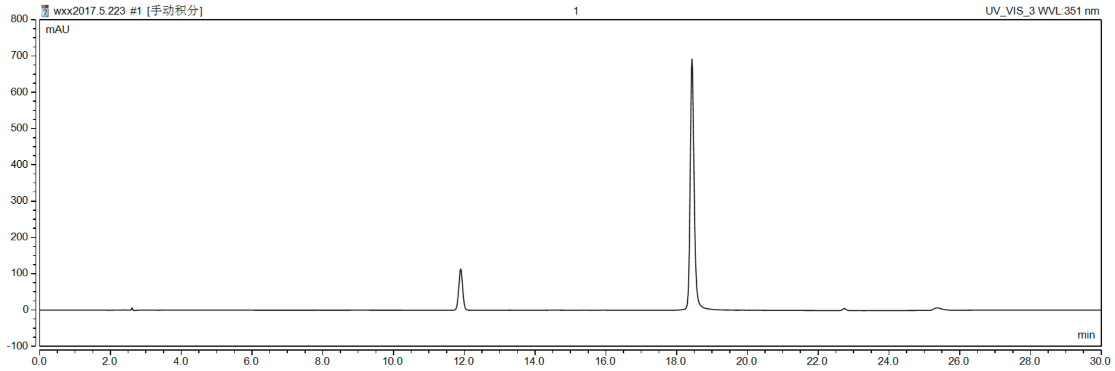
wxx2017.5.223 #1 [手动积分]
1
UV_VIS_3 WVL:351 nm
mAU
min

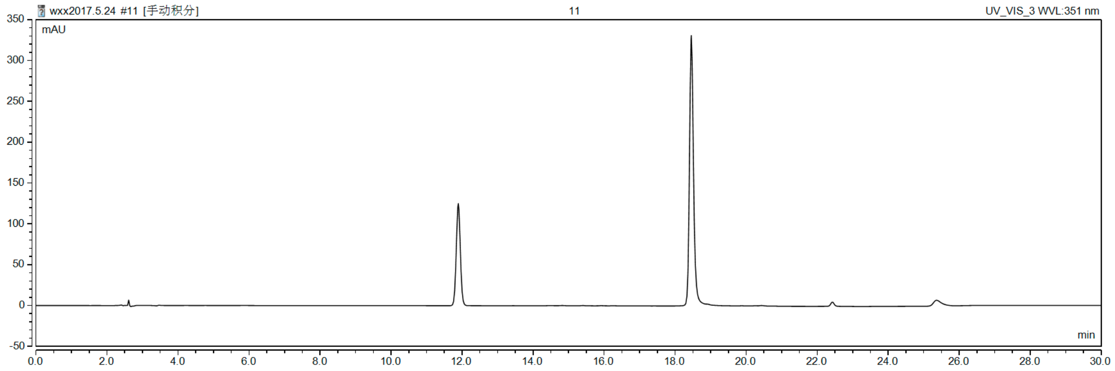
wxx2017.5.24 #11 [手动积分]
11
UV_VIS_3 WVL:351 nm
mAU
min

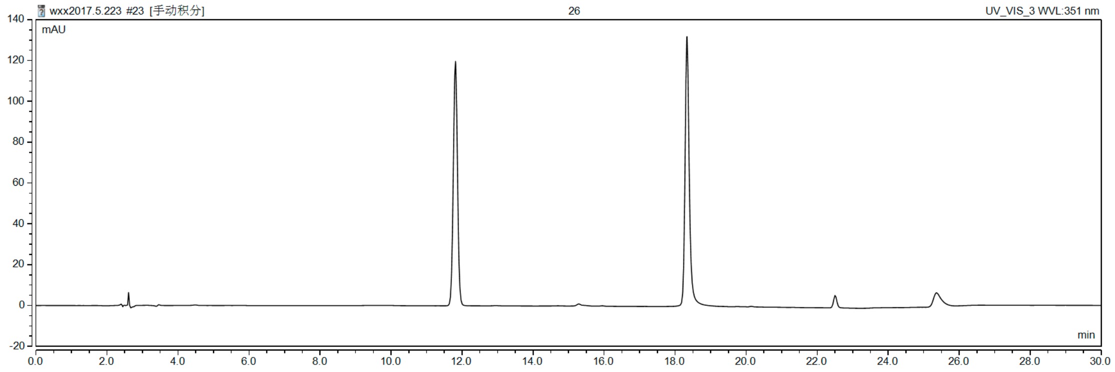
wxx2017.5.223 #23 [手动积分]
26
UV_VIS_3 WVL:351 nm
mAU
min

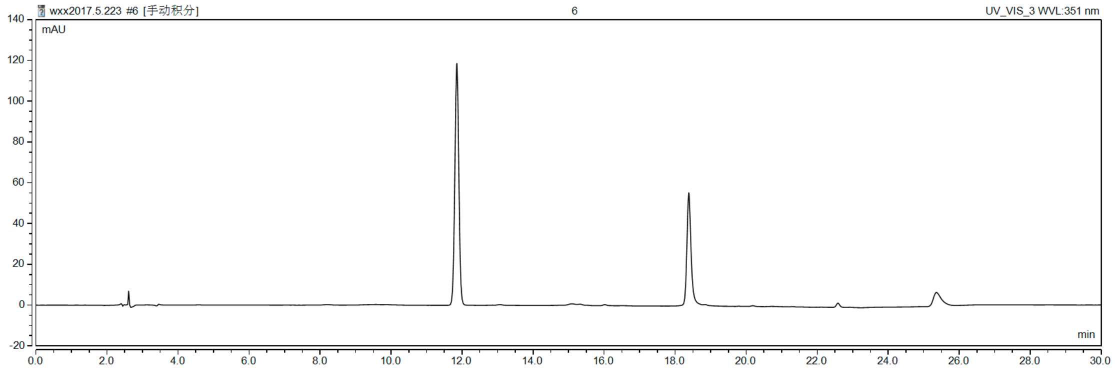

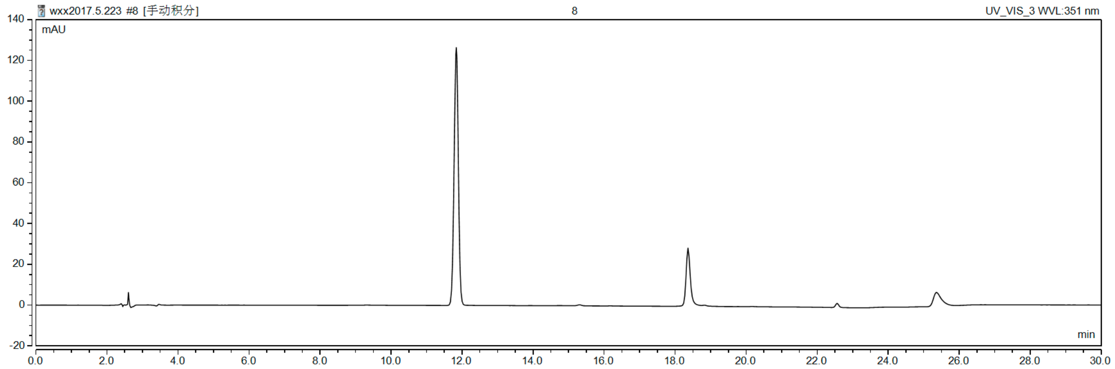

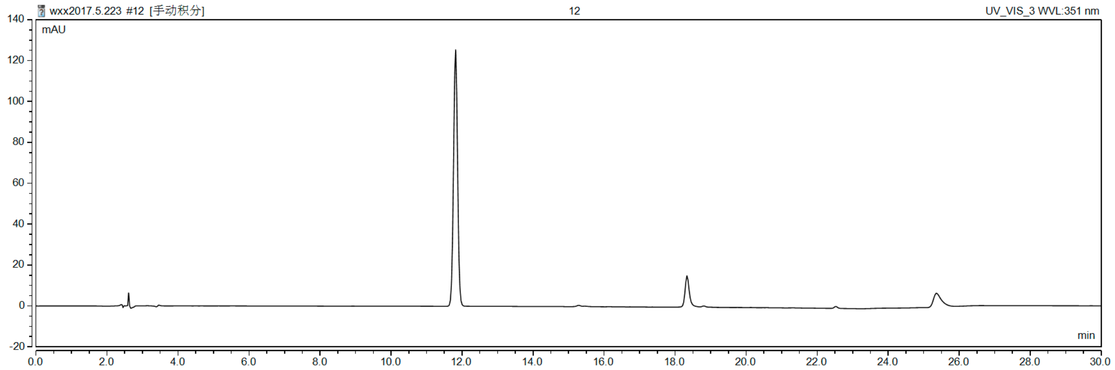

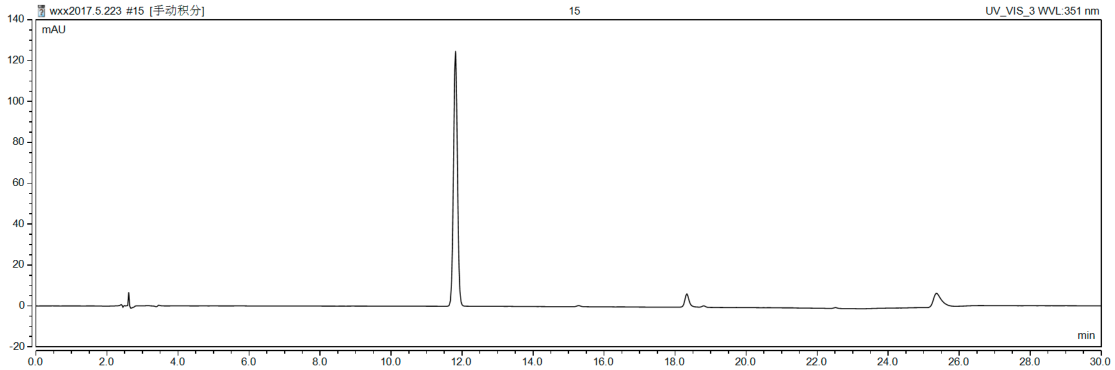

Precision and accuracy

DAY 1

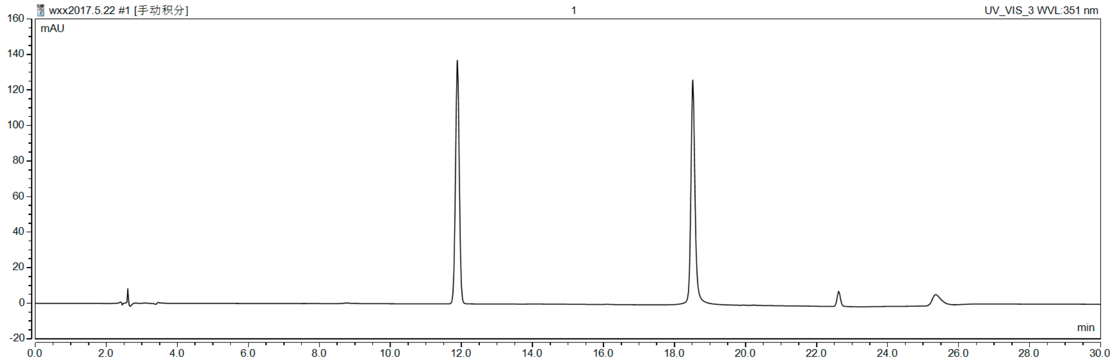
wxx2017.5.22 #1 [手动积分]
1
UV_VIS_3 WVL:351 nm
mAU
min

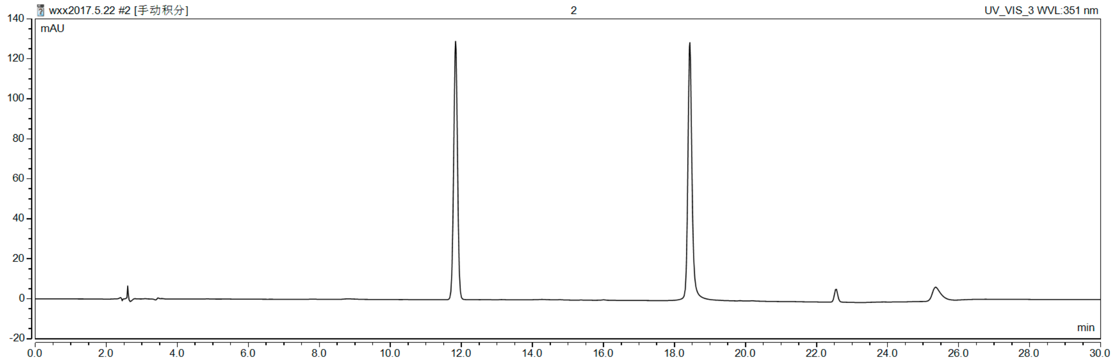
wxx2017.5.22 #2 [手动积分]
2
UV_VIS_3 WVL:351 nm
mAU
min

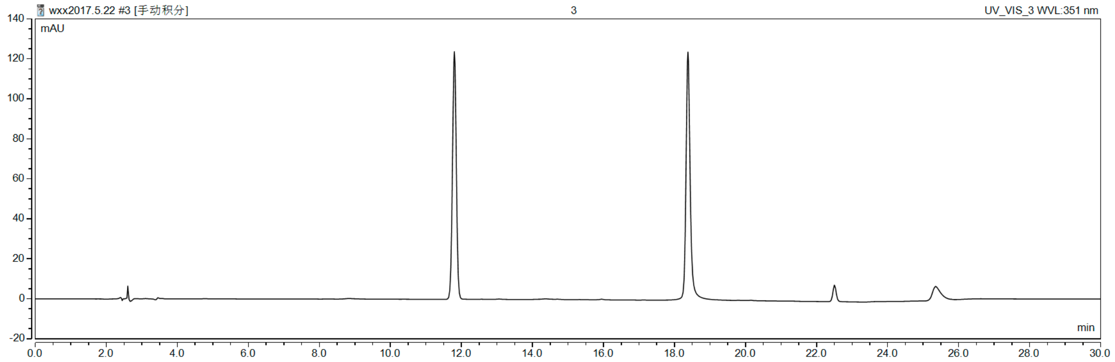
wxx2017.5.22 #3 [手动积分]
3
UV_VIS_3 WVL:351 nm
mAU
min

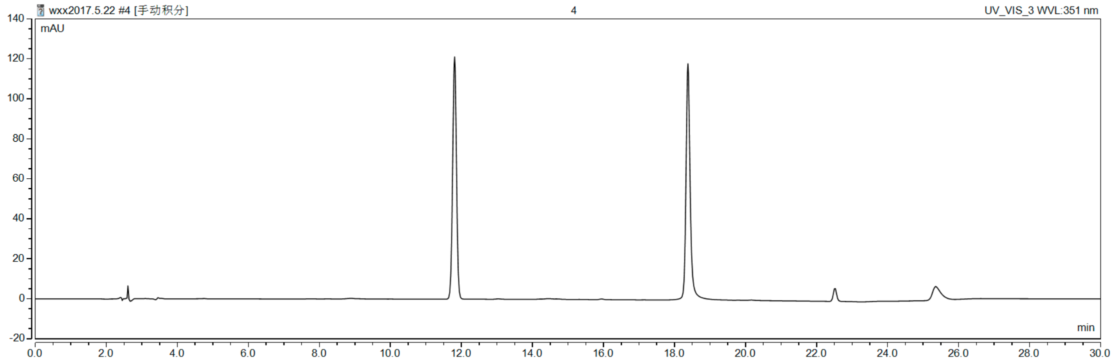
wxx2017.5.22 #4 [手动积分]
4
UV_VIS_3 WVL:351 nm
mAU
min

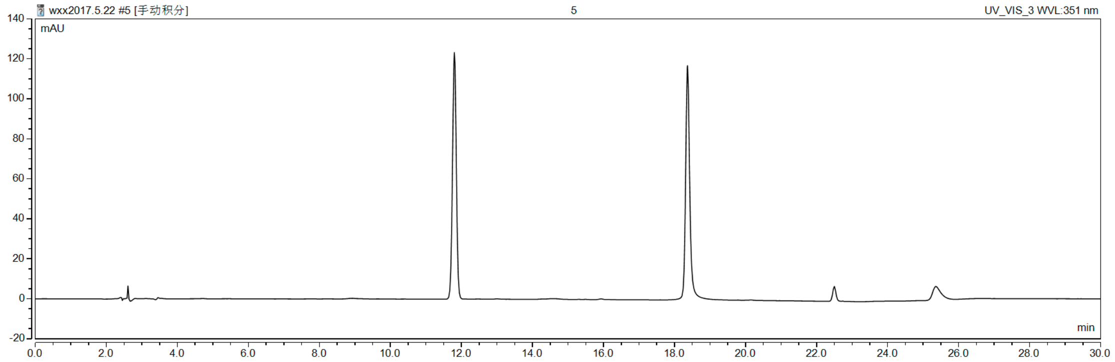
wxx2017.5.22 #5 [手动积分]
5
UV_VIS_3 WVL:351 nm
mAU
min

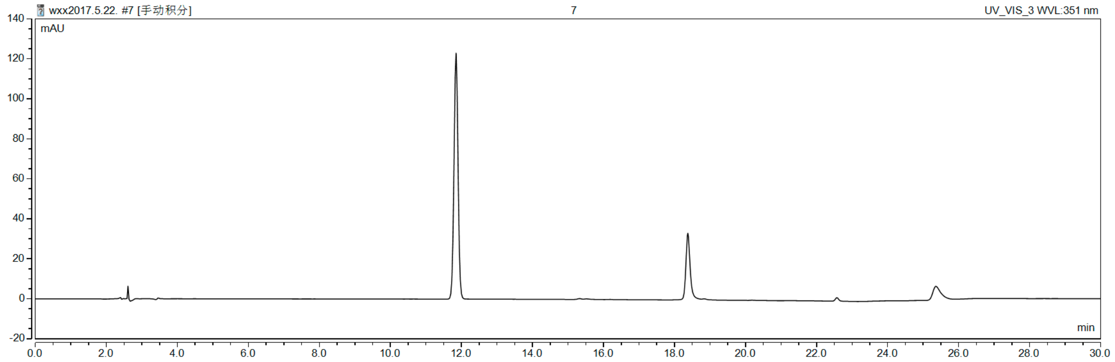
wxx2017.5.22. #7 [手动积分]
7
UV_VIS_3 WVL:351 nm
mAU
min

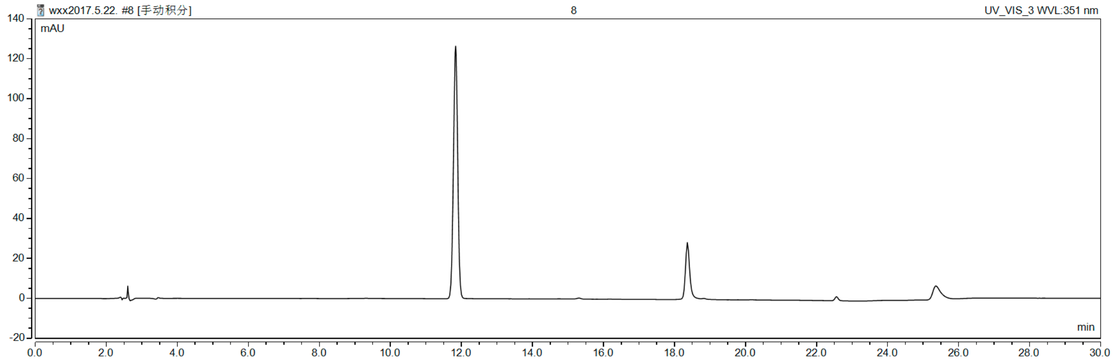
wxx2017.5.22. #8 [手动积分]
8
UV_VIS_3 WVL:351 nm
mAU
min

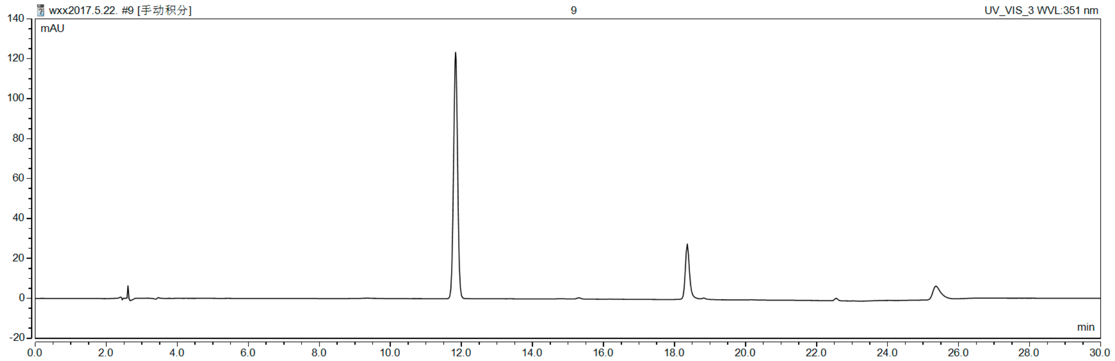
wxx2017.5.22. #9 [手动积分]
9
UV_VIS_3 WVL:351 nm
mAU
min

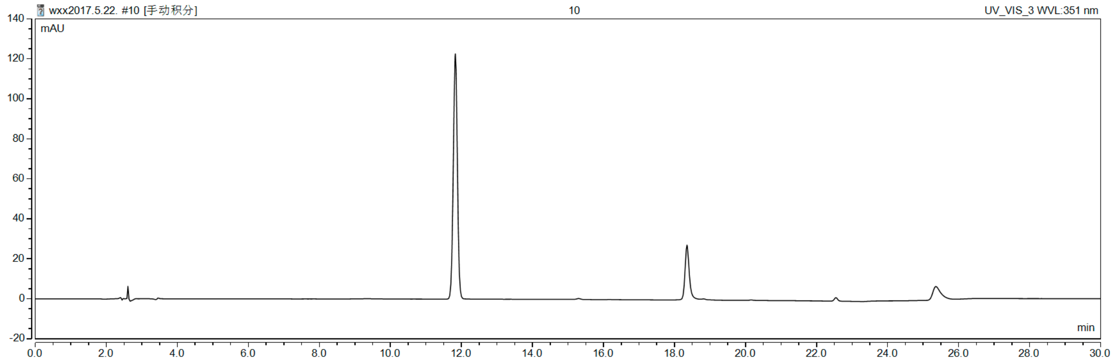
wxx2017.5.22. #10 [手动积分]
10
UV_VIS_3 WVL:351 nm
mAU
min

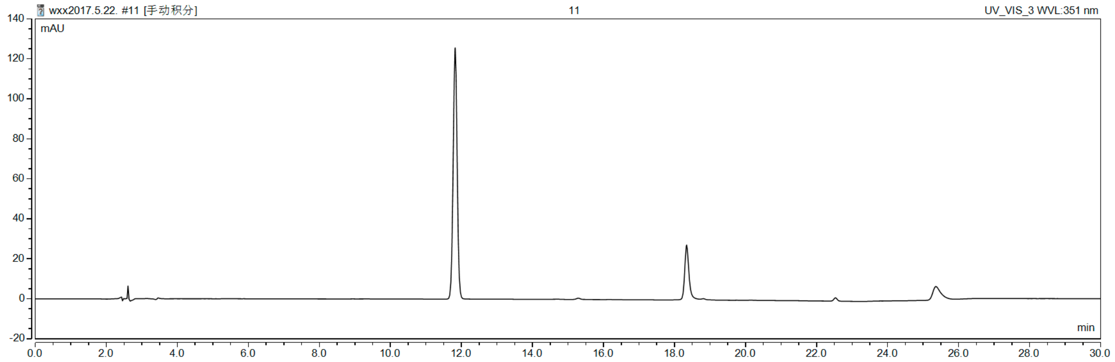
wxx2017.5.22. #11 [手动积分]
11
UV_VIS_3 WVL:351 nm
mAU
min

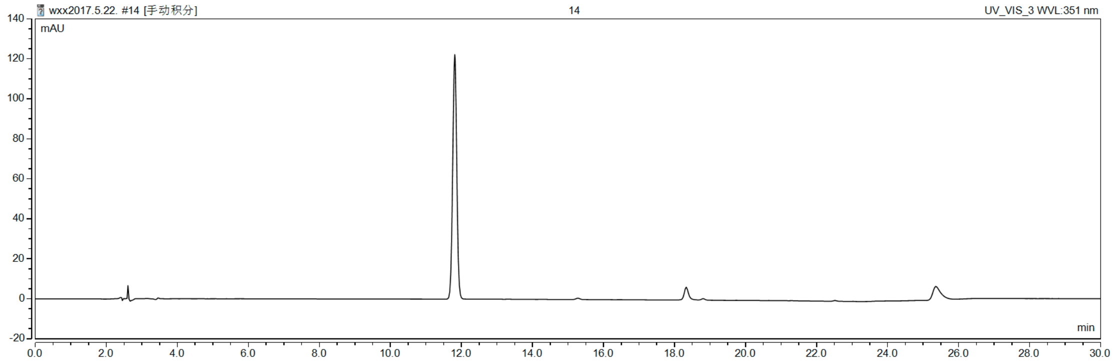
wxx2017.5.22. #14 [手动积分]
14
UV_VIS_3 WVL:351 nm
mAU
min

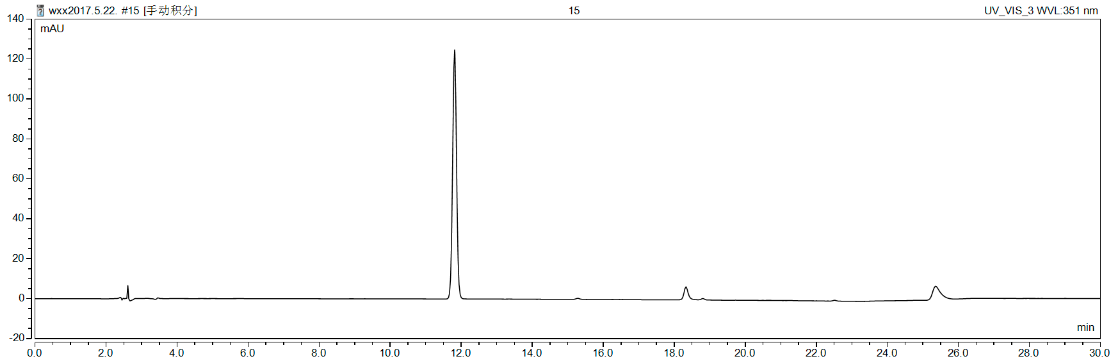
wxx2017.5.22. #15 [手动积分]
15
UV_VIS_3 WVL:351 nm
mAU
min

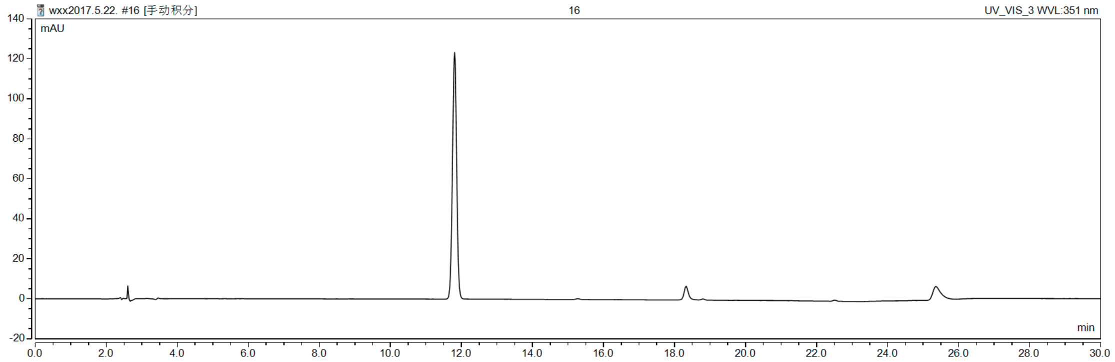
wxx2017.5.22. #16 [手动积分]
16
UV_VIS_3 WVL:351 nm
mAU
min

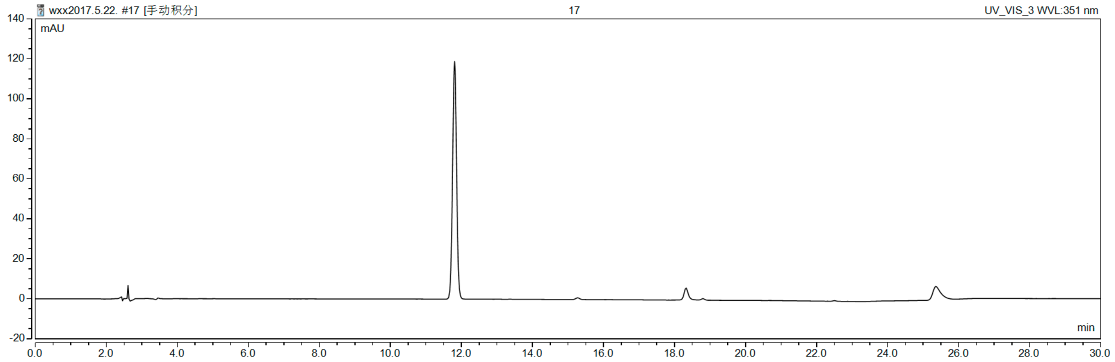
wxx2017.5.22. #17 [手动积分]
17
UV_VIS_3 WVL:351 nm
mAU
min

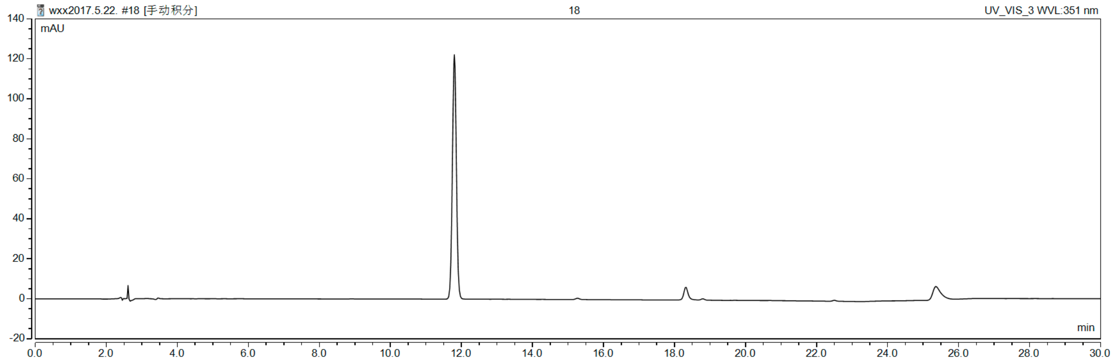
wxx2017.5.22. #18 [手动积分]
18
UV_VIS_3 WVL:351 nm
mAU
min

DAY 2

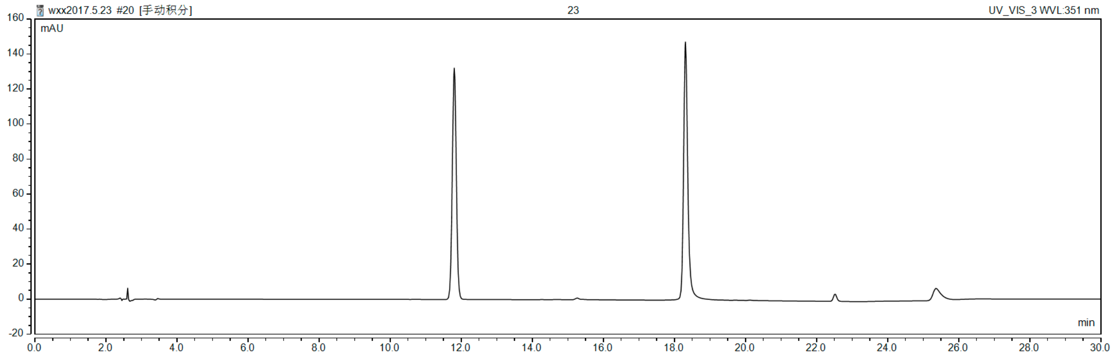
wxx2017.5.23 #20 [手动积分]
23
UV_VIS_3 WVL:351 nm
mAU
min

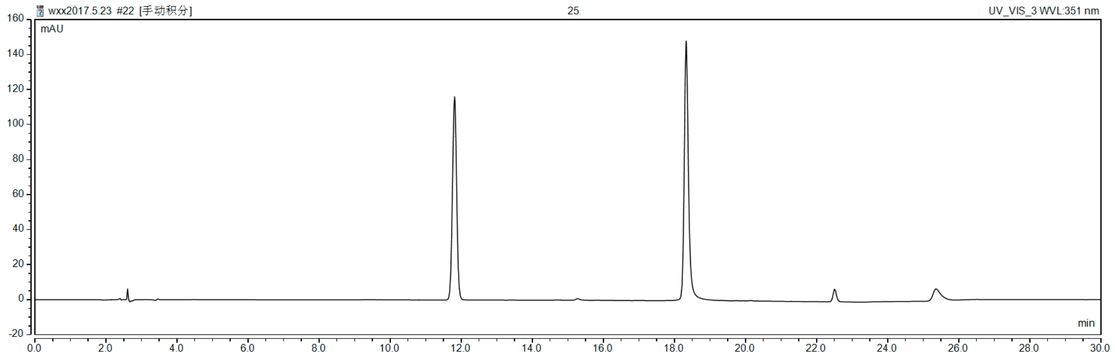
wxx2017.5.23 #22 [手动积分]
25
UV_VIS_3 WVL:351 nm
mAU
min

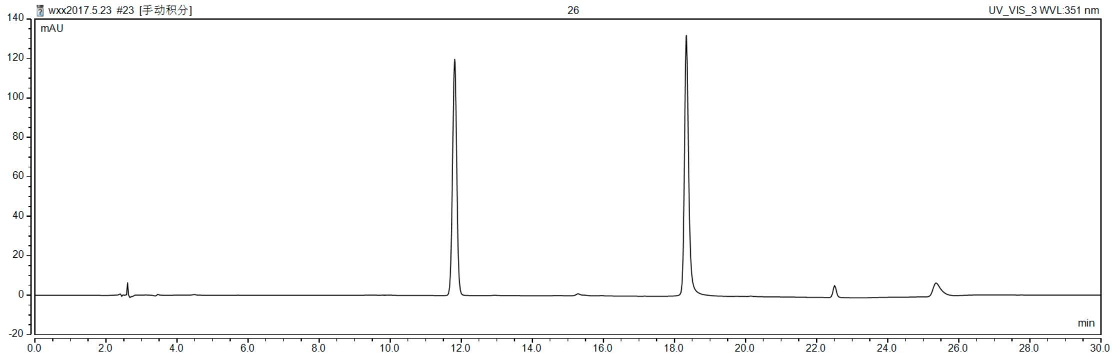
wxx2017.5.23 #23 [手动积分]
26
UV_VIS_3 WVL:351 nm
mAU
min

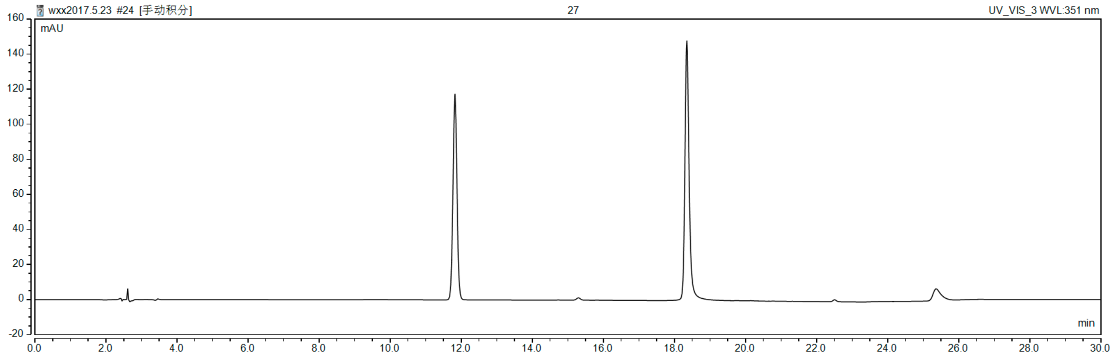
wxx2017.5.23 #24 [手动积分]
27
UV_VIS_3 WVL:351 nm
mAU
min

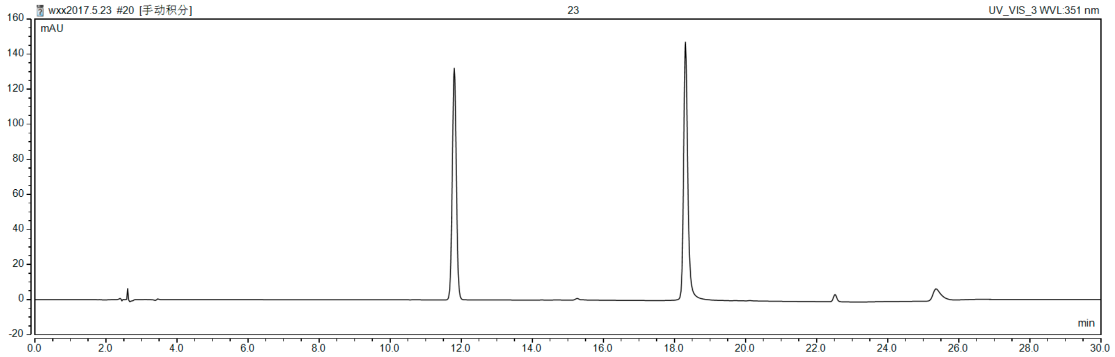
wxx2017.5.23 #20 [手动积分]
23
UV_VIS_3 WVL:351 nm
mAU
min

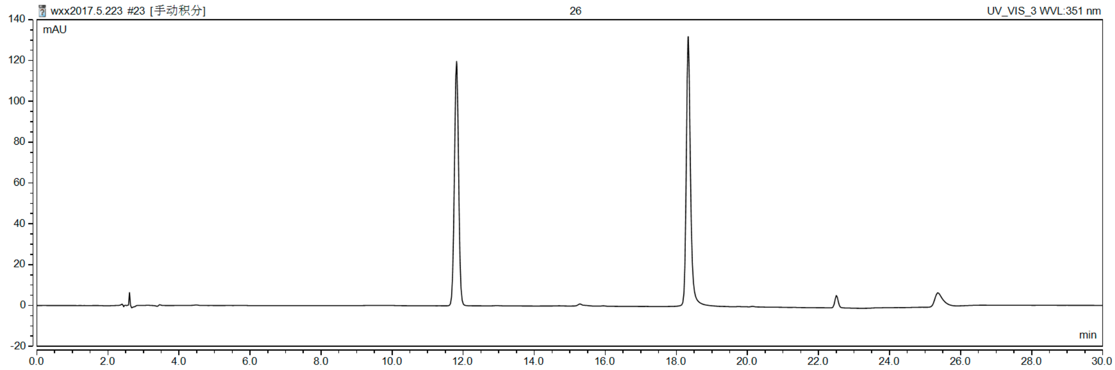
wxx2017.5.223 #23 [手动积分]
26
UV_VIS_3 WVL:351 nm
mAU
min

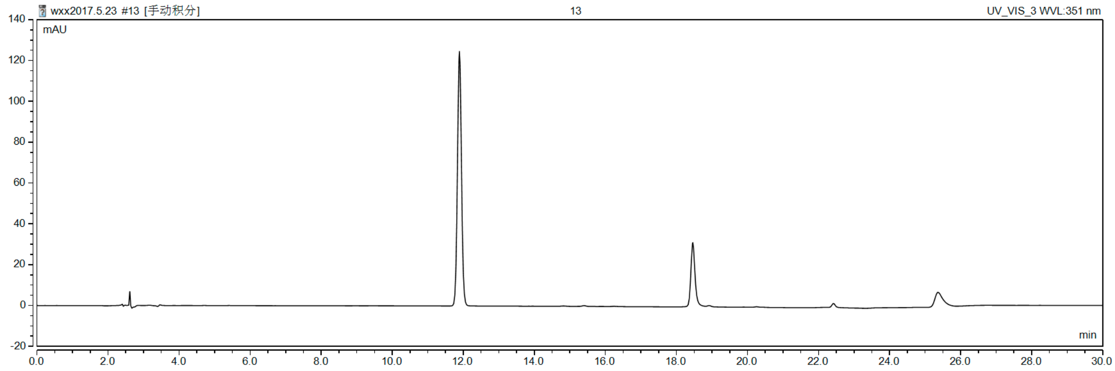
wxx2017.5.23 #13 [手动积分]
13
UV_VIS_3 WVL:351 nm
mAU
min

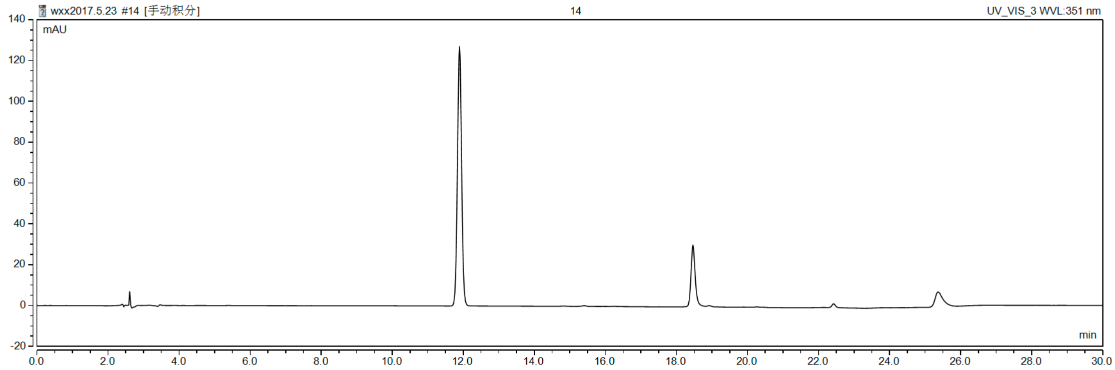
wxx2017.5.23 #14 [手动积分]
14
UV_VIS_3 WVL:351 nm
mAU
min

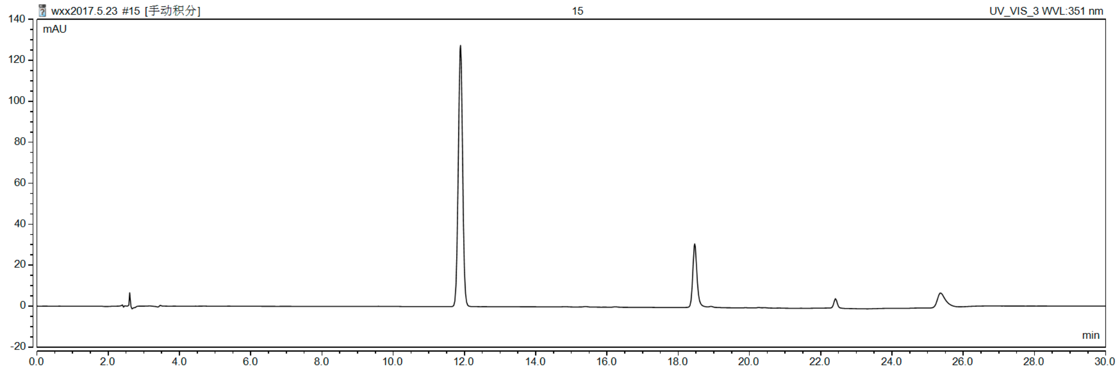
wxx2017.5.23 #15 [手动积分]
15
UV_VIS_3 WVL:351 nm
mAU
min

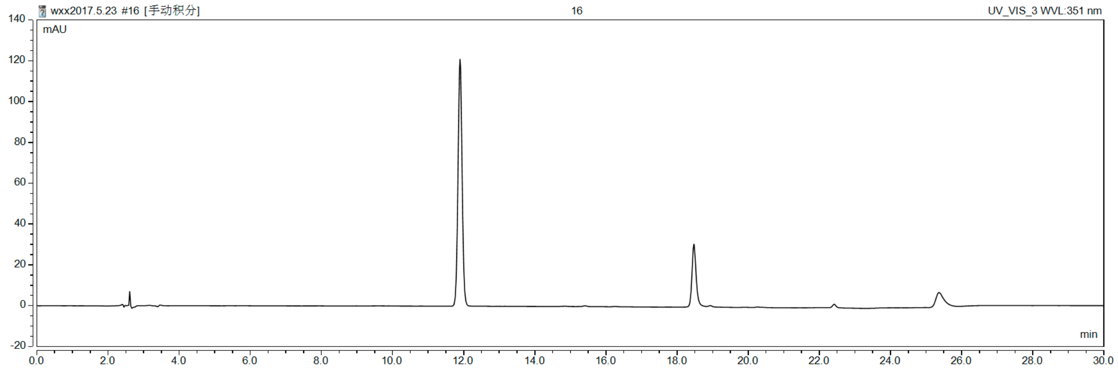
wxx2017.5.23 #16 [手动积分]
16
UV_VIS_3 WVL:351 nm
mAU
min
140
120
100
80
60
40
20
0
-20
0.0
2.0
4.0
6.0
8.0
10.0
12.0
14.0
16.0
18.0
20.0
22.0
24.0
26.0
28.0
30.0

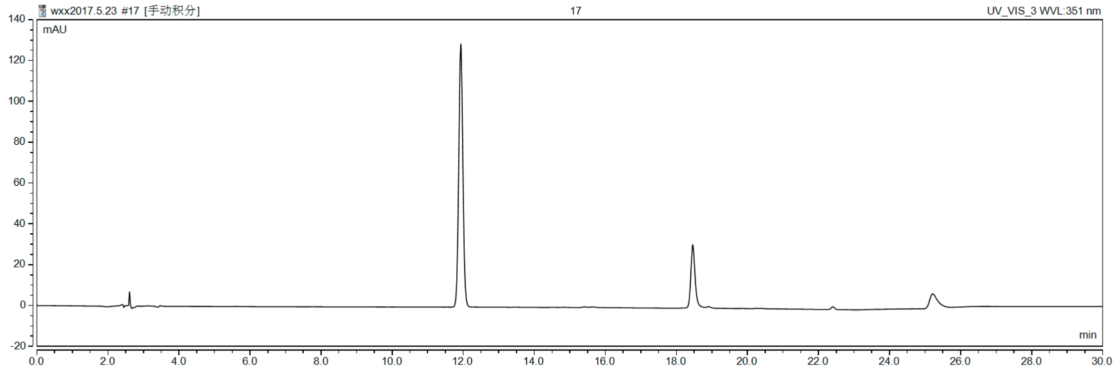
wxx2017.5.23 #17 [手动积分]
17
UV_VIS_3 WVL:351 nm
mAU
min
140
120
100
80
60
40
20
0
-20
0.0
2.0
4.0
6.0
8.0
10.0
12.0
14.0
16.0
18.0
20.0
22.0
24.0
26.0
28.0
30.0

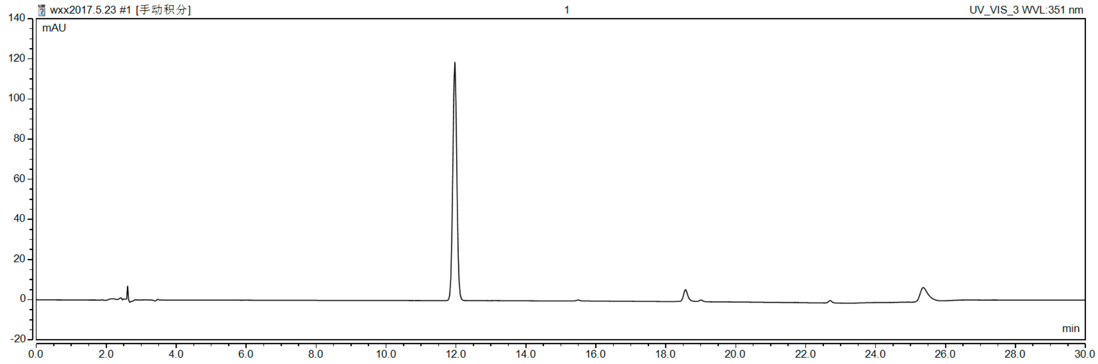
wxx2017.5.23 #1 [手动积分]
1
UV_VIS_3 WVL:351 nm
mAU
min
140
120
100
80
60
40
20
0
-20
0.0
2.0
4.0
6.0
8.0
10.0
12.0
14.0
16.0
18.0
20.0
22.0
24.0
26.0
28.0
30.0

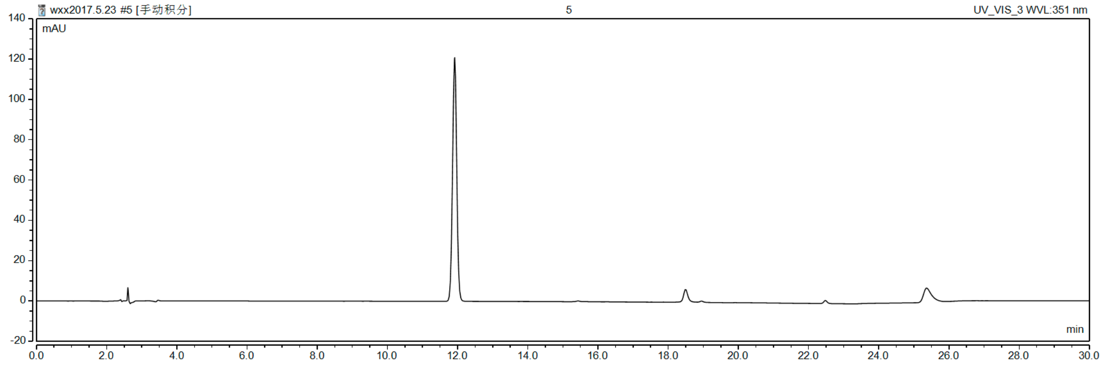
wxx2017.5.23 #5 [手动积分]
5
UV_VIS_3 WVL:351 nm
mAU
min
140
120
100
80
60
40
20
0
-20
0.0
2.0
4.0
6.0
8.0
10.0
12.0
14.0
16.0
18.0
20.0
22.0
24.0
26.0
28.0
30.0

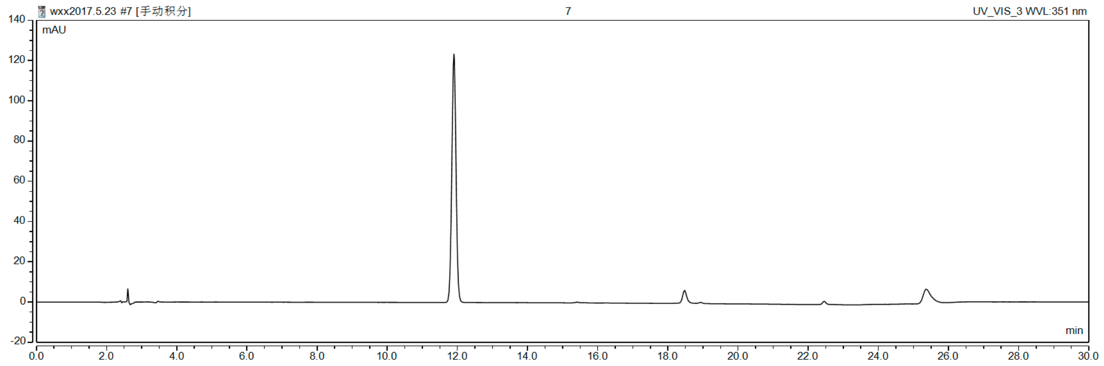
wxx2017.5.23 #7 [手动积分]
7
UV_VIS_3 WVL:351 nm
mAU
min

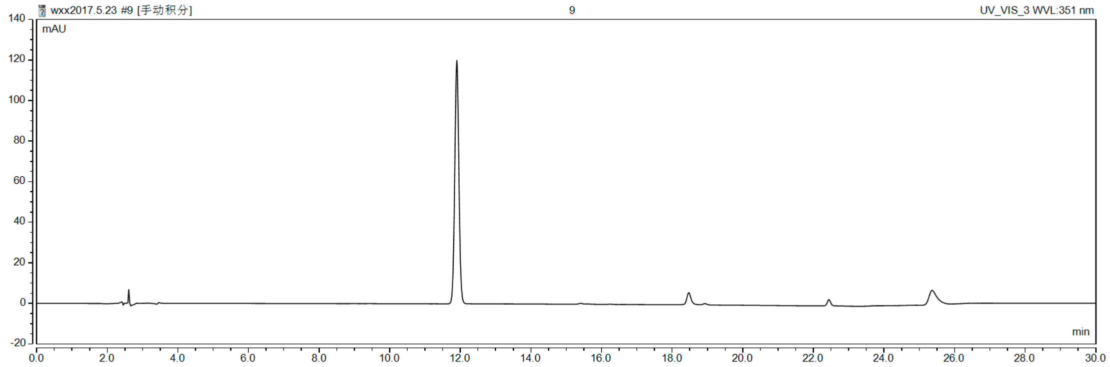
wxx2017.5.23 #9 [手动积分]
9
UV_VIS_3 WVL:351 nm
mAU
min

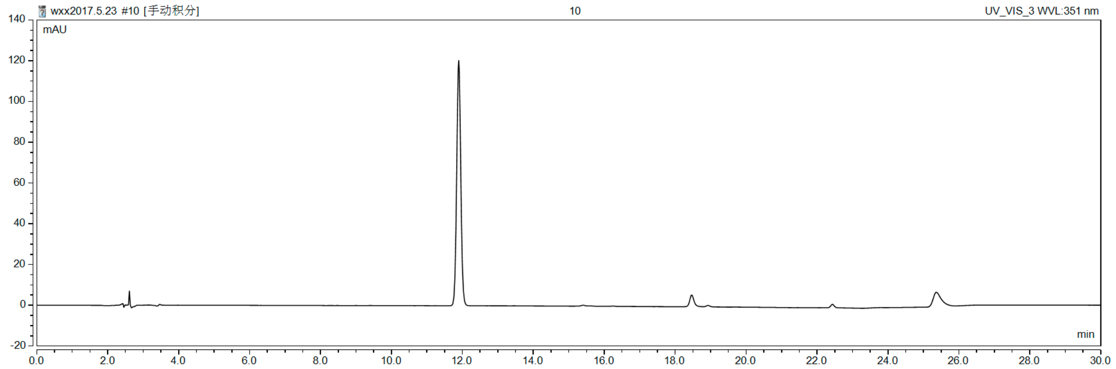
wxx2017.5.23 #10 [手动积分]
10
UV_VIS_3 WVL:351 nm
mAU
min

DAY 3

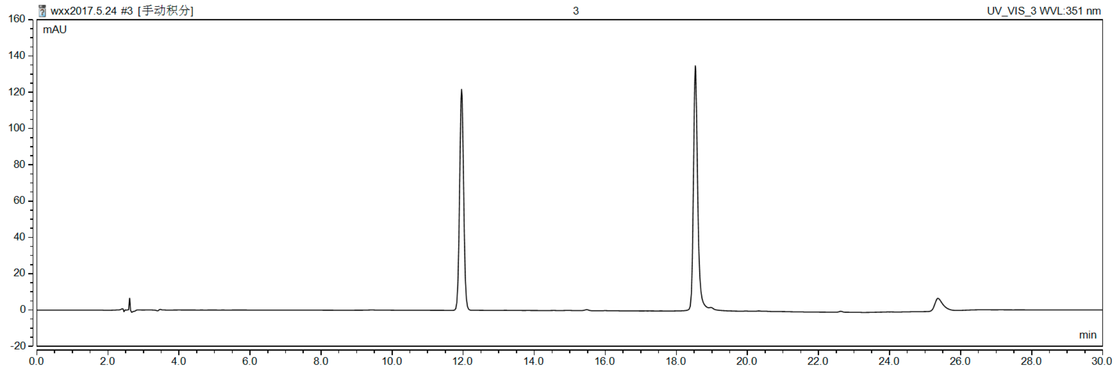
wxx2017.5.24 #3 [手动积分]
3
UV_VIS_3 WVL:351 nm
mAU
min

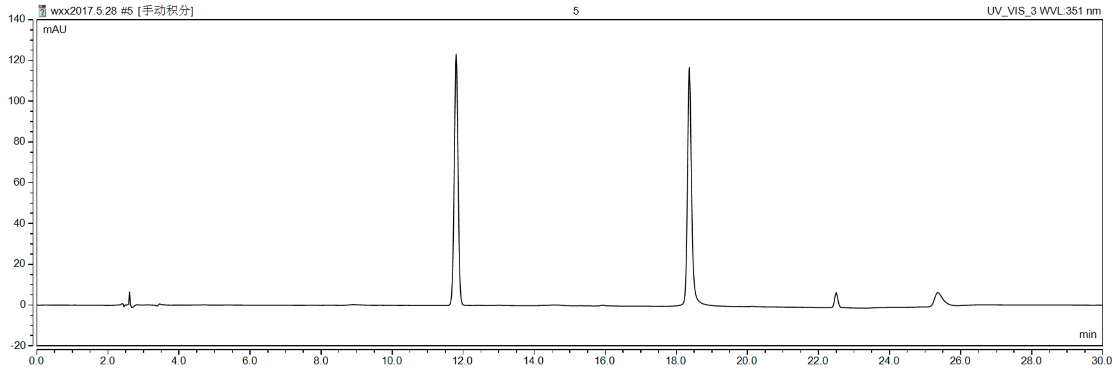
wxx2017.5.28 #5 [手动积分]
5
UV_VIS_3 WVL:351 nm
mAU
min

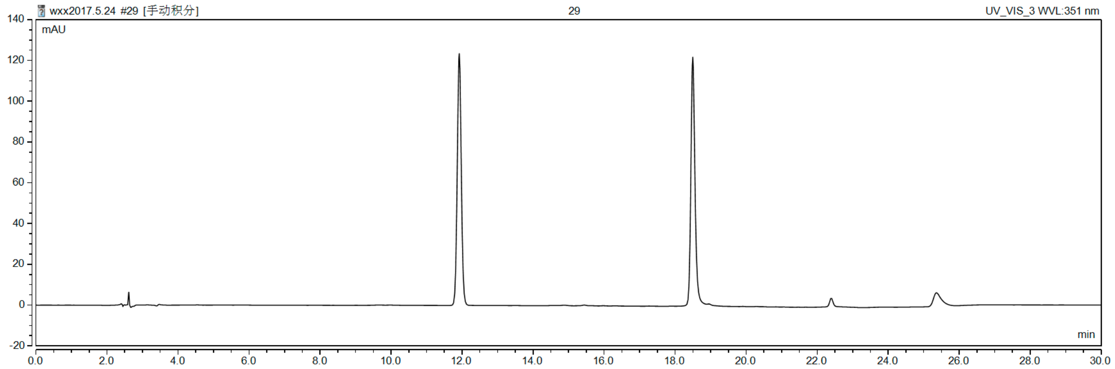
wxx2017.5.24 #29 [手动积分]
29
UV_VIS_3 WVL:351 nm
mAU
min

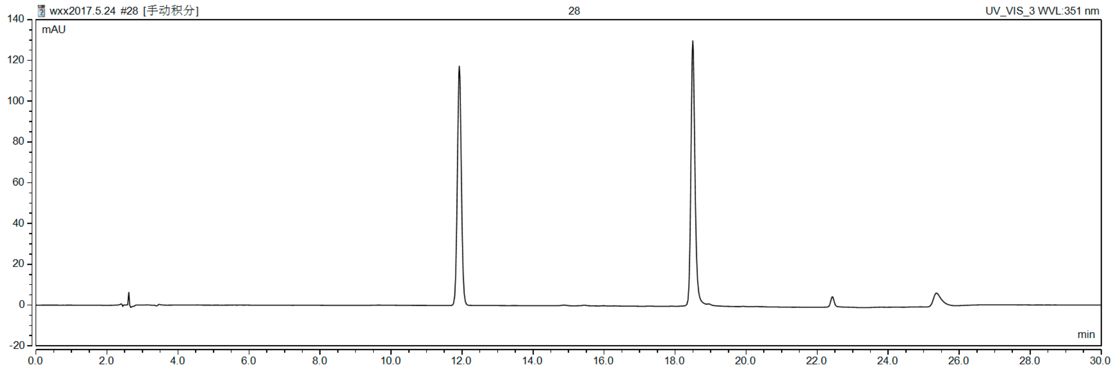
wxx2017.5.24 #28 [手动积分]
28
UV_VIS_3 WVL:351 nm
mAU
min

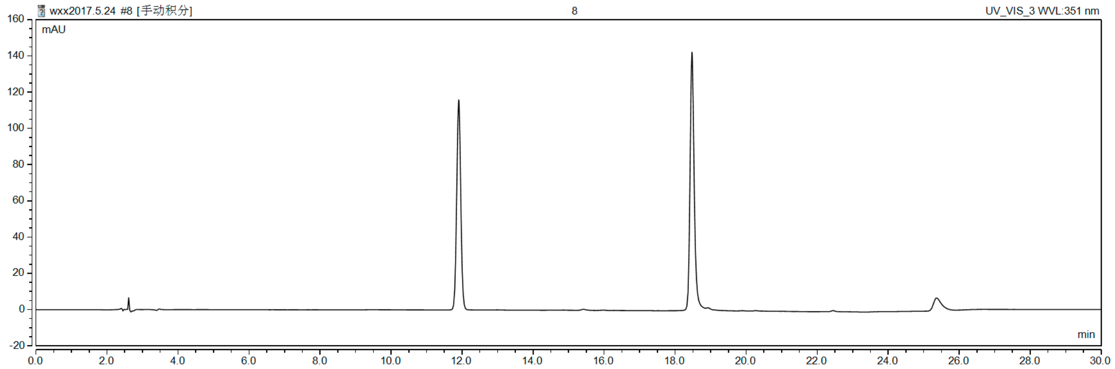
wxx2017.5.24 #8 [手动积分]
8
UV_VIS_3 WVL:351 nm
mAU
min

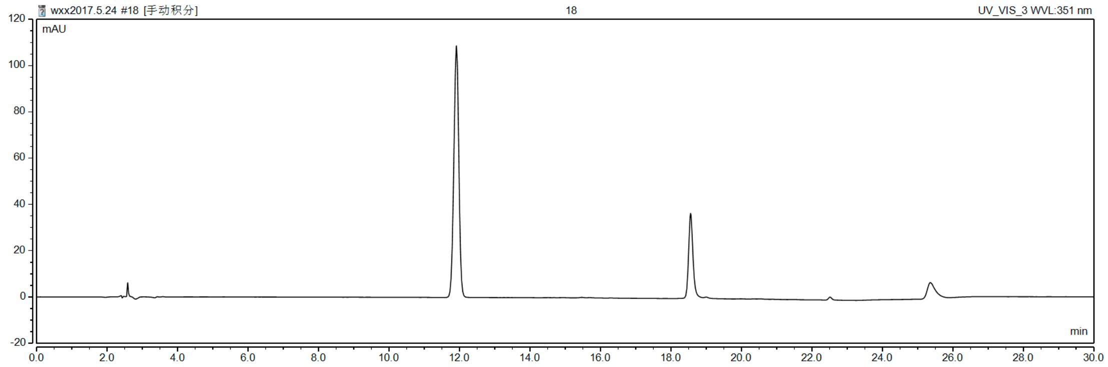
wxx2017.5.24 #18 [手动积分]
18
UV_VIS_3 WVL:351 nm
mAU
min

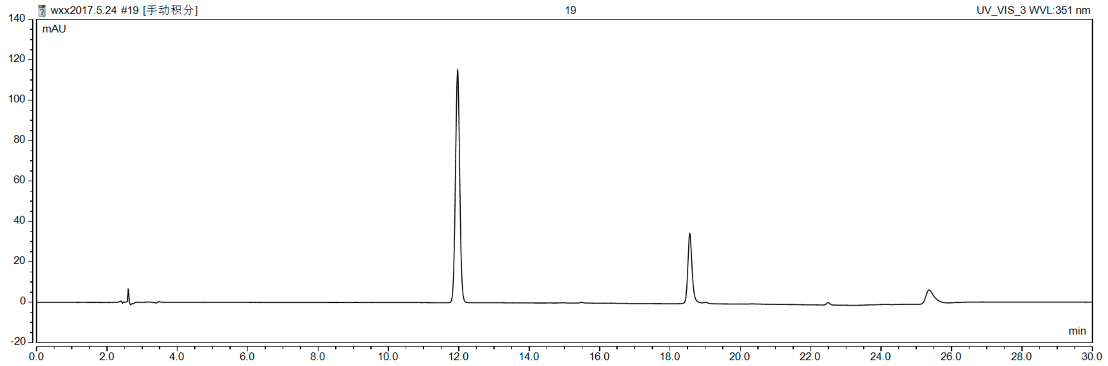
wxx2017.5.24 #19 [手动积分]
19
UV_VIS_3 WVL:351 nm
mAU
min

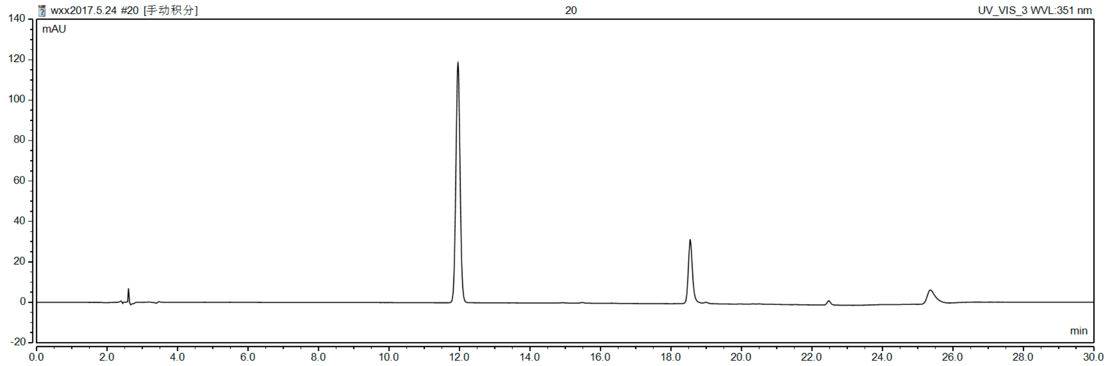
wxx2017.5.24 #20 [手动积分]
20
UV_VIS_3 WVL:351 nm
mAU
min

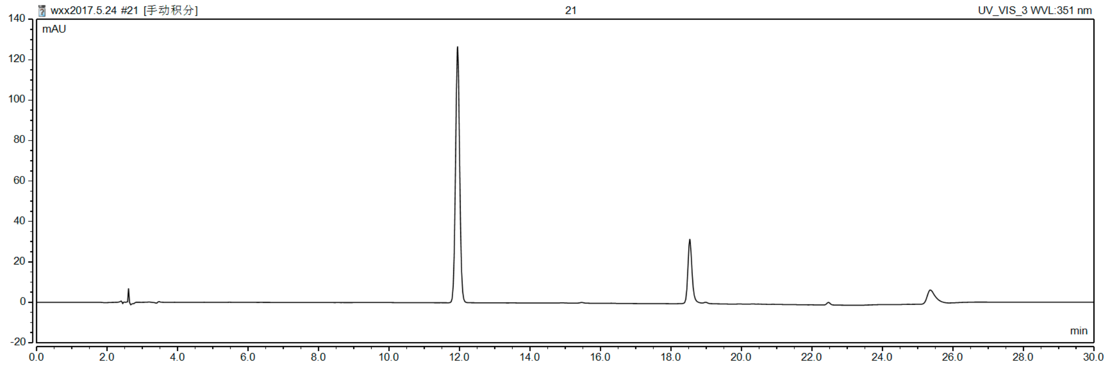
wxx2017.5.24 #21 [手动积分]
21
UV_VIS_3 WVL:351 nm
mAU
min

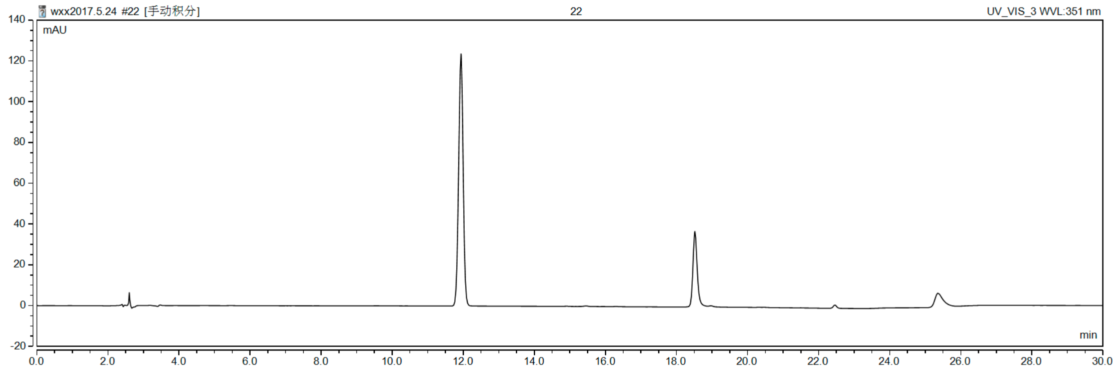
wxx2017.5.24 #22 [手动积分]
22
UV_VIS_3 WVL:351 nm
mAU
min

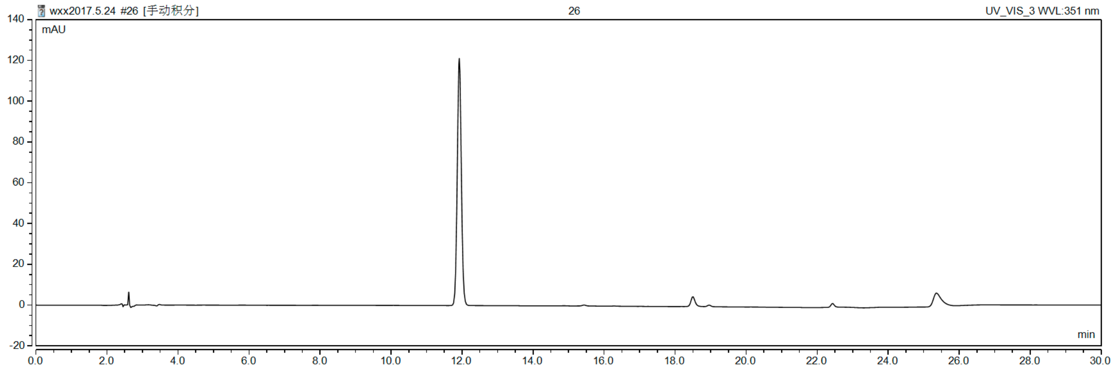
wxx2017.5.24 #26 [手动积分]
26
UV_VIS_3 WVL:351 nm
mAU
min

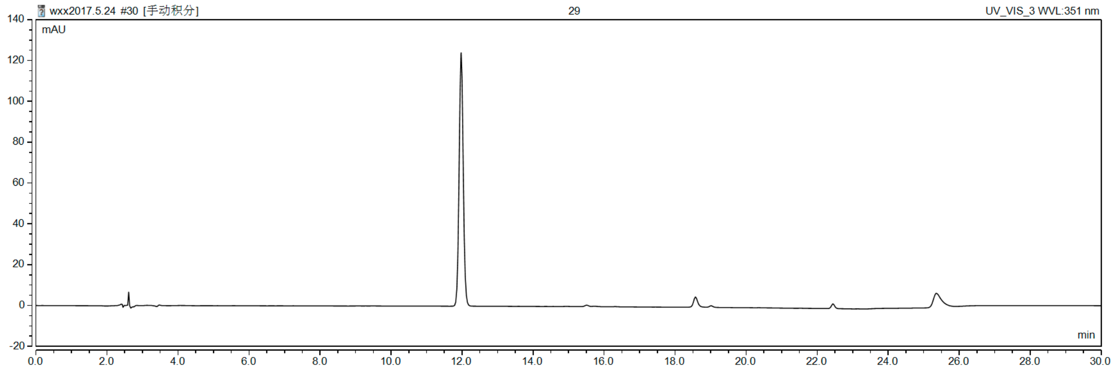
wxx2017.5.24 #30 [手动积分]
29
UV_VIS_3 WVL:351 nm
mAU
min

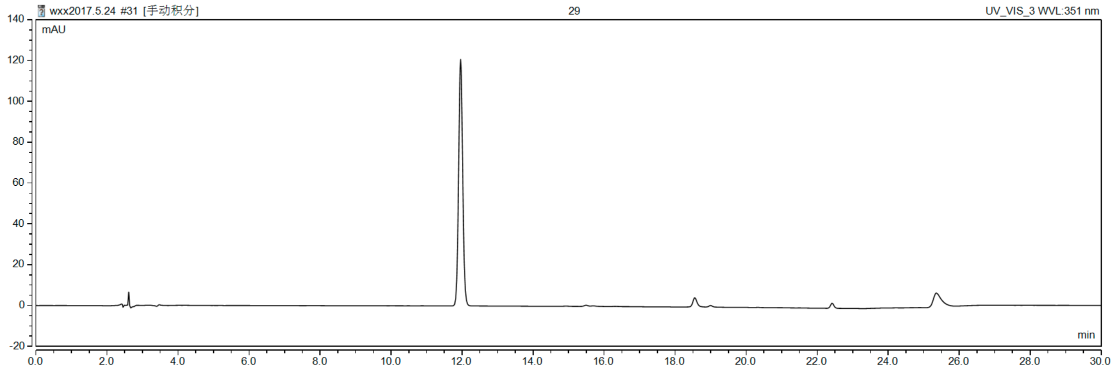
wxx2017.5.24 #31 [手动积分]
29
UV_VIS_3 WVL:351 nm
mAU
min

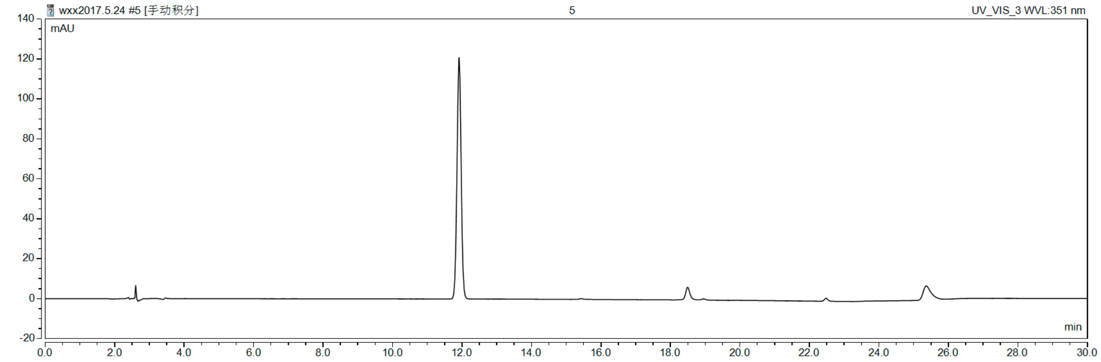
wxx2017.5.24 #5 [手动积分]
5
UV_VIS_3 WVL:351 nm
mAU
min

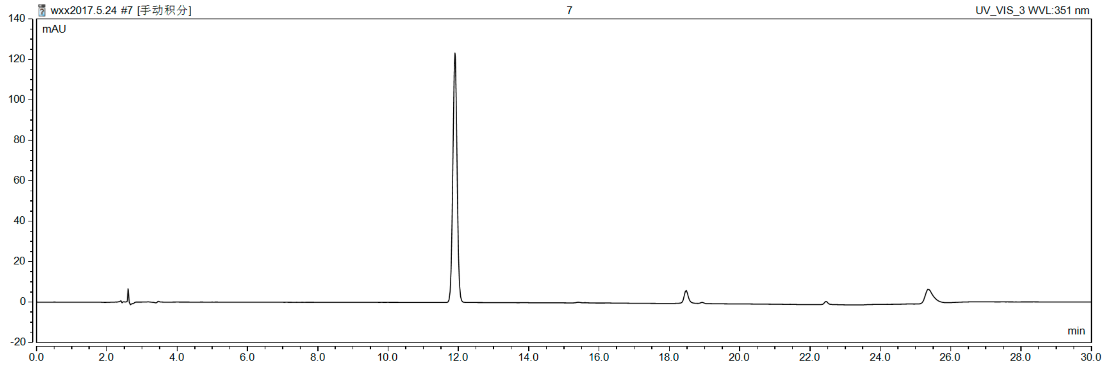
wxx2017.5.24 #7 [手动积分]
7
UV_VIS_3 WVL:351 nm
mAU
min

Recovery

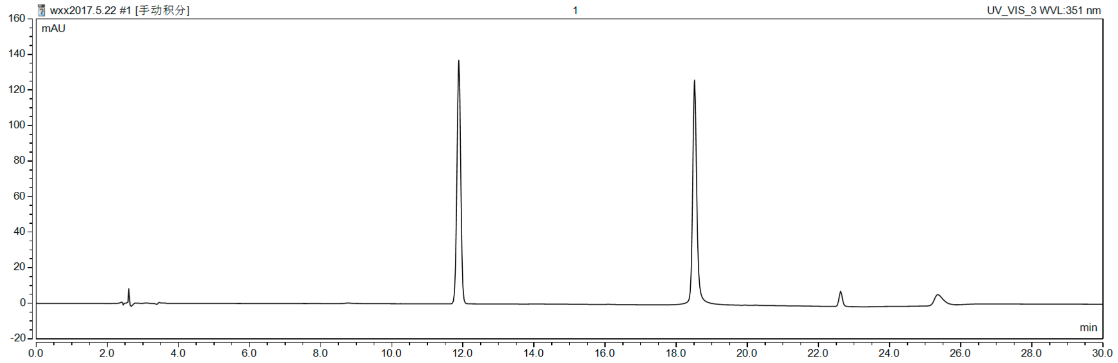
wxx2017.5.22 #1 [手动积分]
1
UV_VIS_3 WVL:351 nm
mAU
min

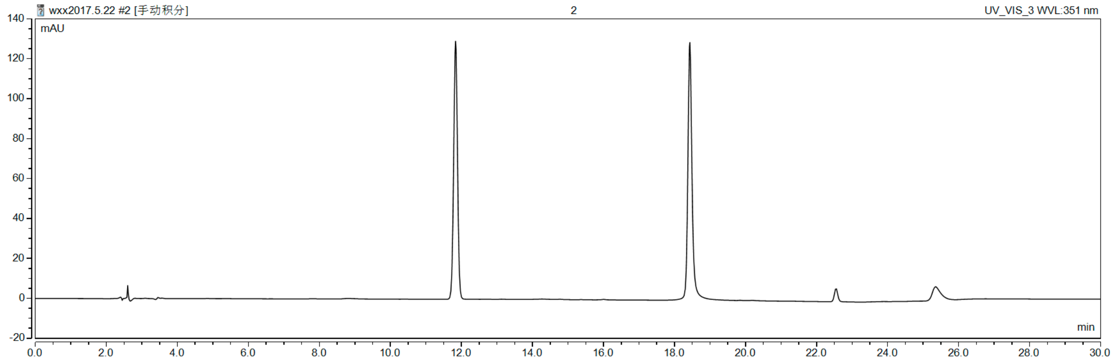
wxx2017.5.22 #2 [手动积分]
2
UV_VIS_3 WVL:351 nm
mAU
min

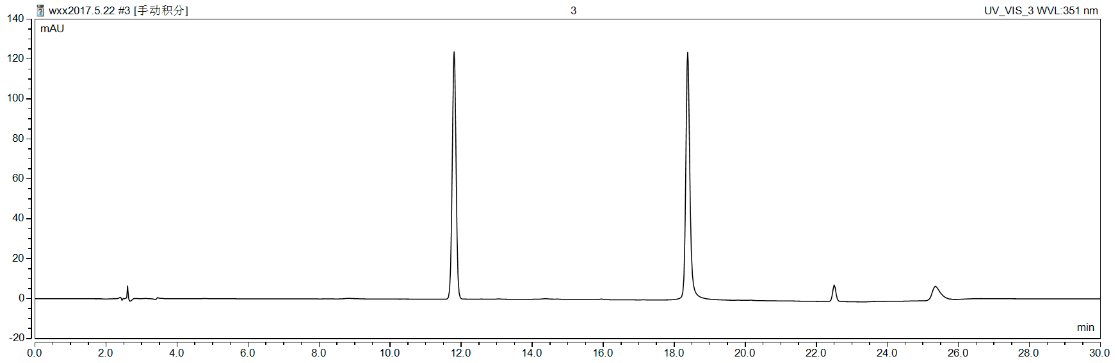
wxx2017.5.22 #3 [手动积分]
3
UV_VIS_3 WVL:351 nm
mAU
min
140
120
100
80
60
40
20
0
-20
0.0 2.0 4.0 6.0 8.0 10.0 12.0 14.0 16.0 18.0 20.0 22.0 24.0 26.0 28.0 30.0

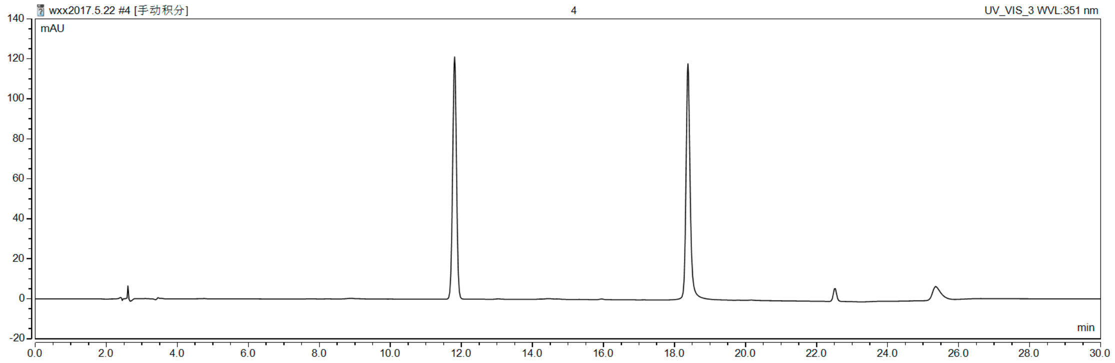
wxx2017.5.22 #4 [手动积分]
4
UV_VIS_3 WVL:351 nm
mAU
min
140
120
100
80
60
40
20
0
-20
0.0 2.0 4.0 6.0 8.0 10.0 12.0 14.0 16.0 18.0 20.0 22.0 24.0 26.0 28.0 30.0

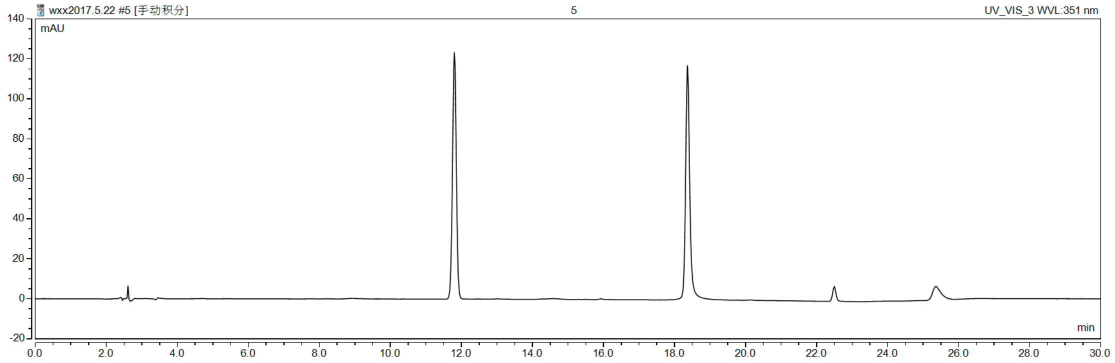
wxx2017.5.22 #5 [手动积分]
5
UV_VIS_3 WVL:351 nm
mAU
min
140
120
100
80
60
40
20
0
-20
0.0 2.0 4.0 6.0 8.0 10.0 12.0 14.0 16.0 18.0 20.0 22.0 24.0 26.0 28.0 30.0

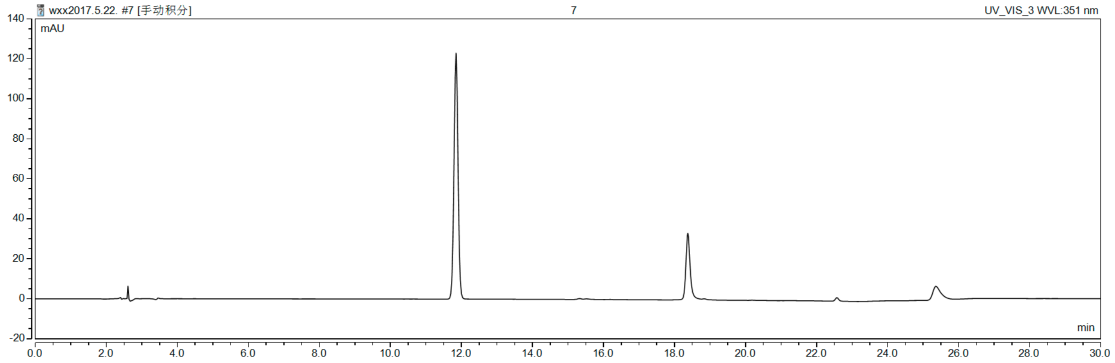
wxx2017.5.22. #7 [手动积分]
7
UV_VIS_3 WVL:351 nm
mAU
min
140
120
100
80
60
40
20
0
-20
0.0 2.0 4.0 6.0 8.0 10.0 12.0 14.0 16.0 18.0 20.0 22.0 24.0 26.0 28.0 30.0

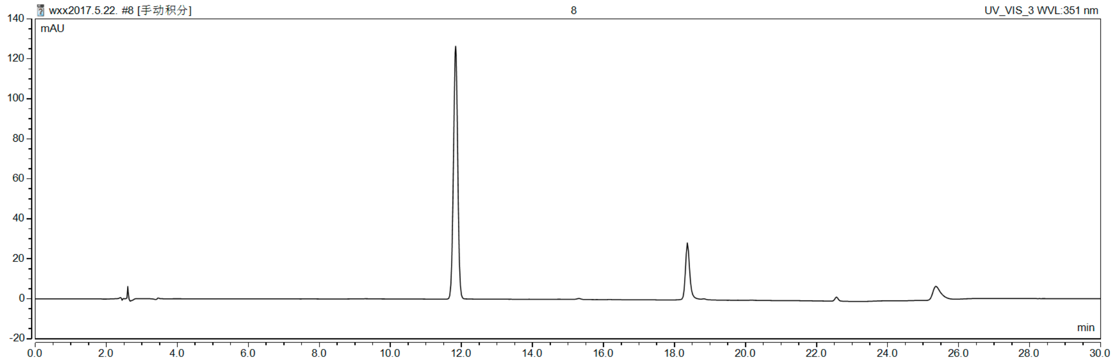
wxx2017.5.22. #8 [手动积分]
8
UV_VIS_3 WVL:351 nm
mAU
min
140
120
100
80
60
40
20
0
-20
0.0
2.0
4.0
6.0
8.0
10.0
12.0
14.0
16.0
18.0
20.0
22.0
24.0
26.0
28.0
30.0

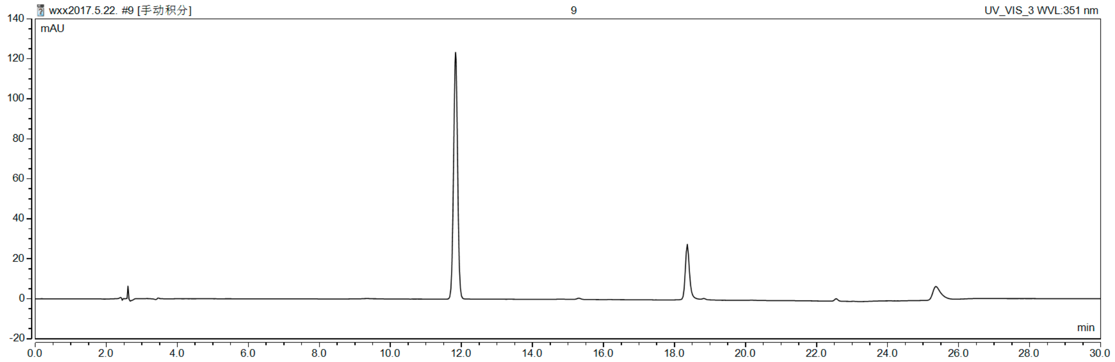
wxx2017.5.22. #9 [手动积分]
9
UV_VIS_3 WVL:351 nm
mAU
min
140
120
100
80
60
40
20
0
-20
0.0
2.0
4.0
6.0
8.0
10.0
12.0
14.0
16.0
18.0
20.0
22.0
24.0
26.0
28.0
30.0

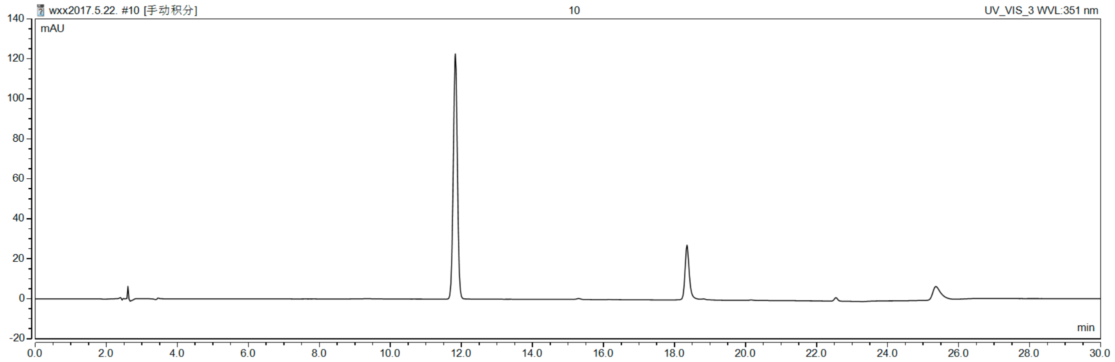
wxx2017.5.22. #10 [手动积分]
10
UV_VIS_3 WVL:351 nm
mAU
min
140
120
100
80
60
40
20
0
-20
0.0
2.0
4.0
6.0
8.0
10.0
12.0
14.0
16.0
18.0
20.0
22.0
24.0
26.0
28.0
30.0

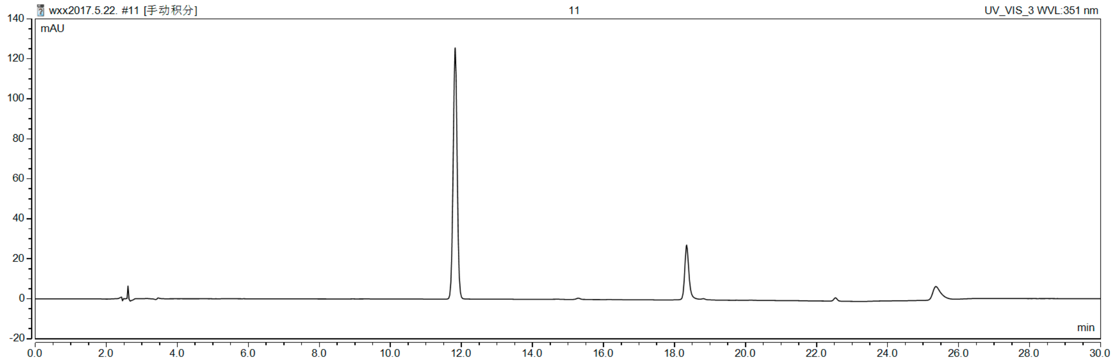
wxx2017.5.22. #11 [手动积分]
11
UV_VIS_3 WVL:351 nm
mAU
min
140
120
100
80
60
40
20
0
-20
0.0
2.0
4.0
6.0
8.0
10.0
12.0
14.0
16.0
18.0
20.0
22.0
24.0
26.0
28.0
30.0

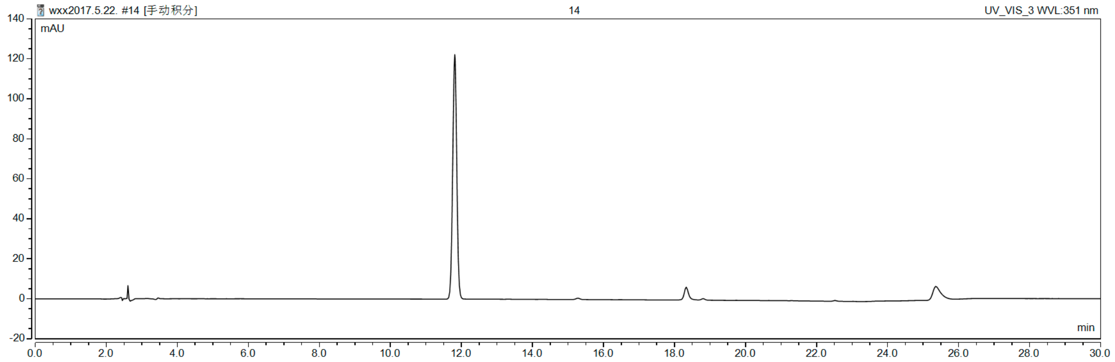
wxx2017.5.22. #14 [手动积分]
14
UV_VIS_3 WVL:351 nm
mAU
min

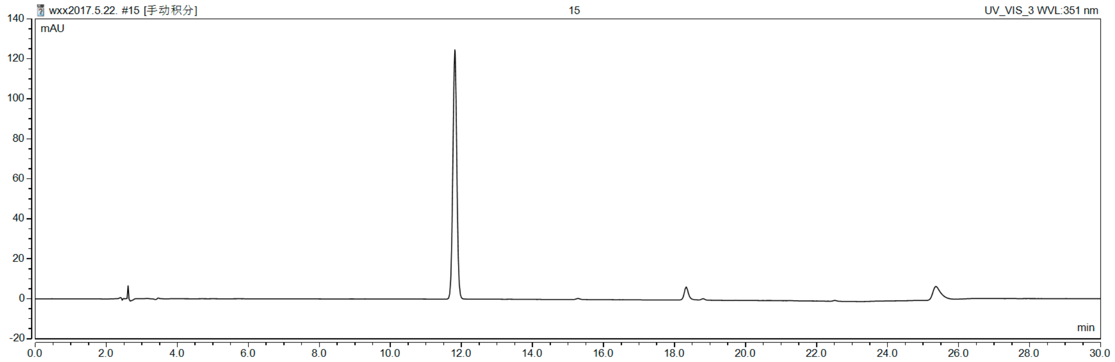
wxx2017.5.22. #15 [手动积分]
15
UV_VIS_3 WVL:351 nm
mAU
min

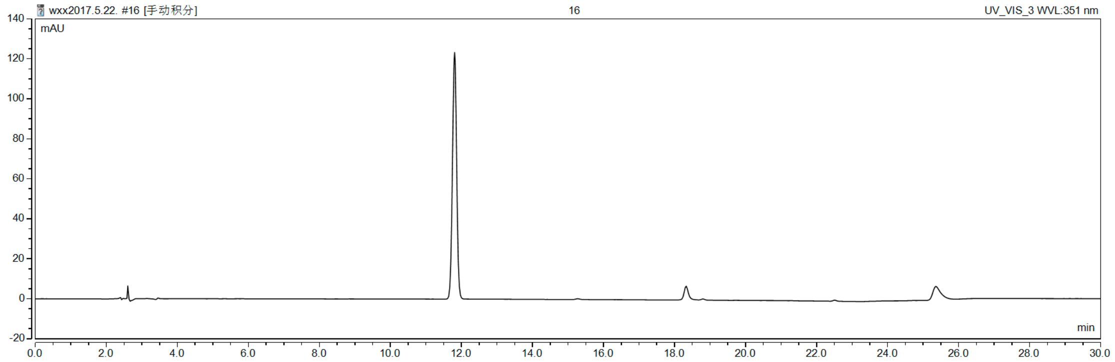
wxx2017.5.22. #16 [手动积分]
16
UV_VIS_3 WVL:351 nm
mAU
min

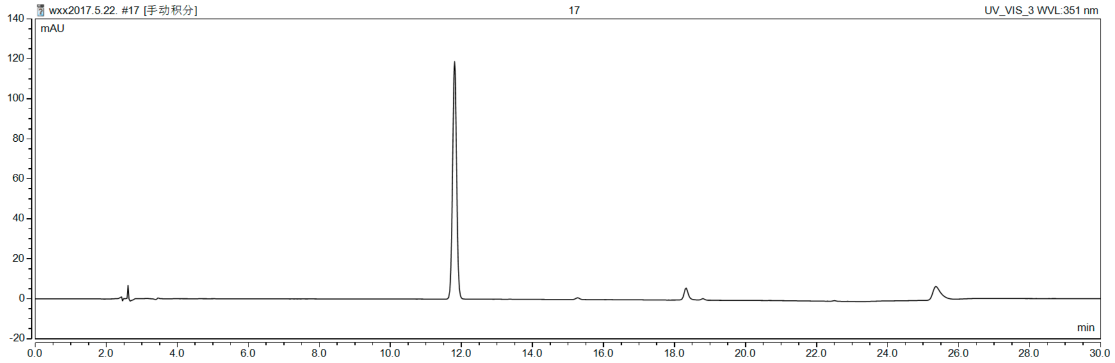
wxx2017.5.22. #17 [手动积分]
17
UV_VIS_3 WVL:351 nm
mAU
min

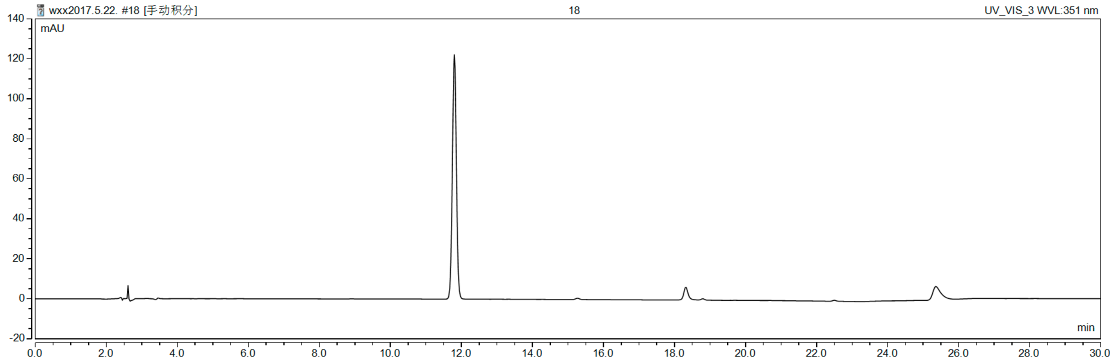
wxx2017.5.22. #18 [手动积分]
18
UV_VIS_3 WVL:351 nm
mAU
min
140
120
100
80
60
40
20
0
-20
0.0
2.0
4.0
6.0
8.0
10.0
12.0
14.0
16.0
18.0
20.0
22.0
24.0
26.0
28.0
30.0

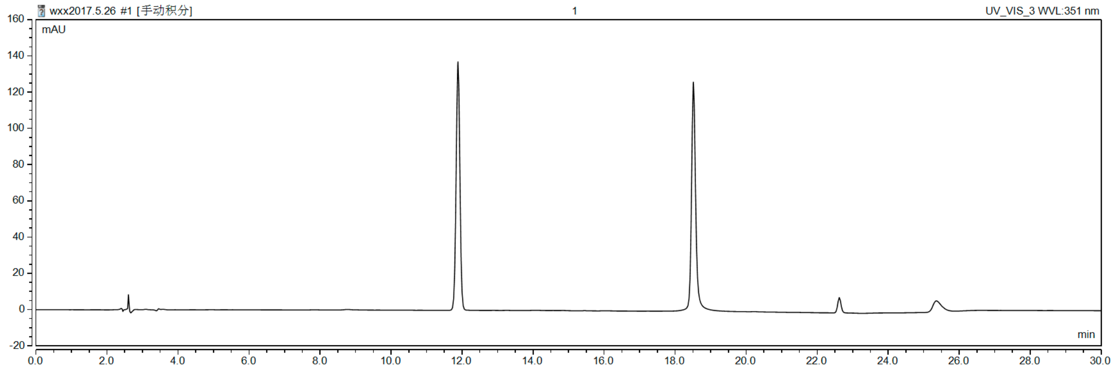
wxx2017.5.26 #1 [手动积分]
1
UV_VIS_3 WVL:351 nm
mAU
min
160
140
120
100
80
60
40
20
0
-20
0.0
2.0
4.0
6.0
8.0
10.0
12.0
14.0
16.0
18.0
20.0
22.0
24.0
26.0
28.0
30.0

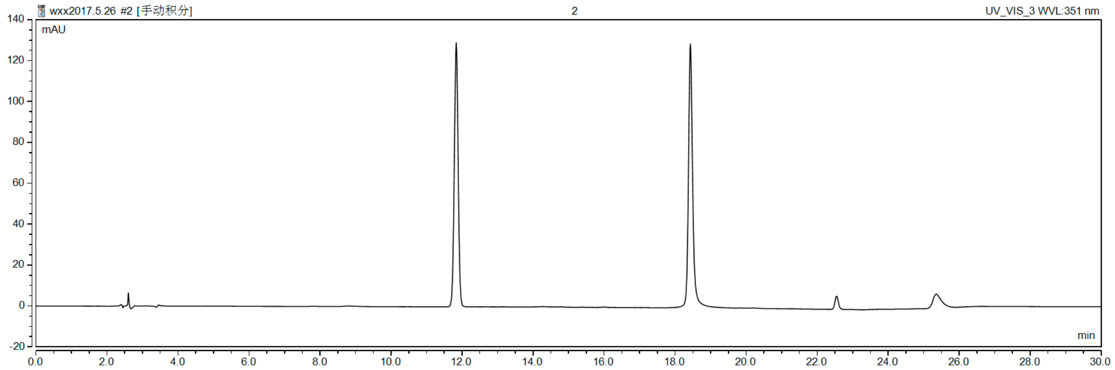
wxx2017.5.26 #2 [手动积分]
2
UV_VIS_3 WVL:351 nm
mAU
min
140
120
100
80
60
40
20
0
-20
0.0
2.0
4.0
6.0
8.0
10.0
12.0
14.0
16.0
18.0
20.0
22.0
24.0
26.0
28.0
30.0

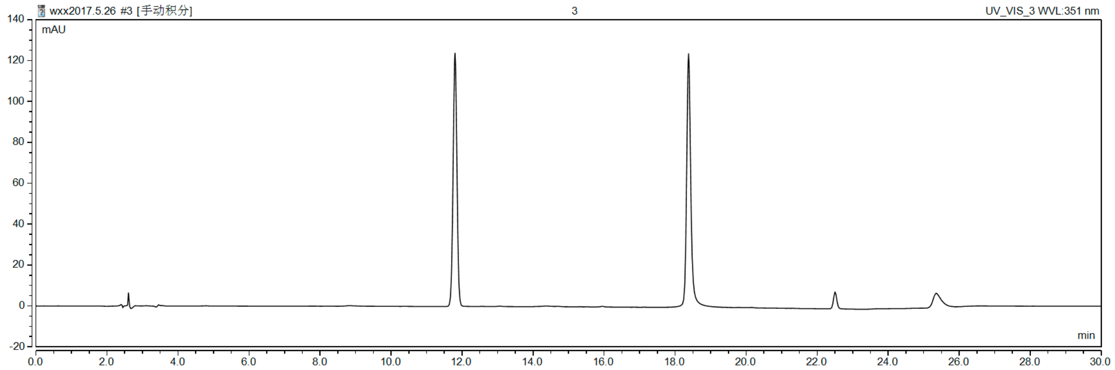
wxx2017.5.26 #3 [手动积分]
3
UV_VIS_3 WVL:351 nm
mAU
min
140
120
100
80
60
40
20
0
-20
0.0
2.0
4.0
6.0
8.0
10.0
12.0
14.0
16.0
18.0
20.0
22.0
24.0
26.0
28.0
30.0

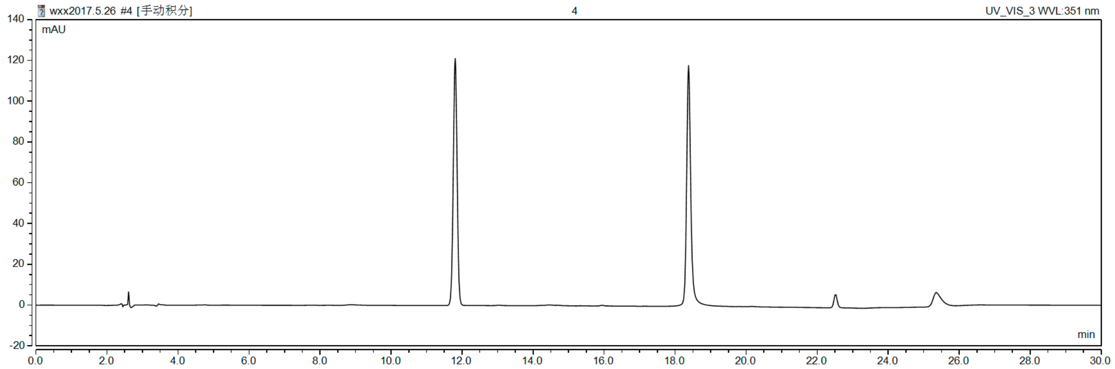
wxx2017.5.26 #4 [手动积分]
4
UV_VIS_3 WVL:351 nm
mAU
min

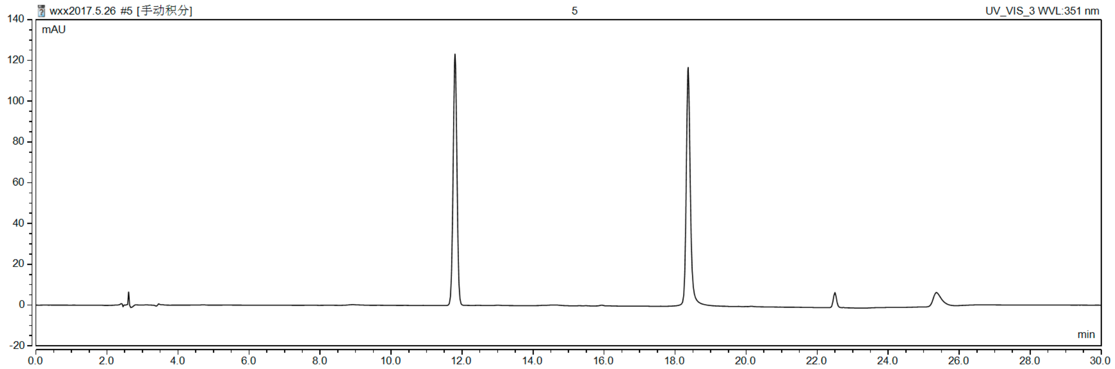
wxx2017.5.26 #5 [手动积分]
5
UV_VIS_3 WVL:351 nm
mAU
min

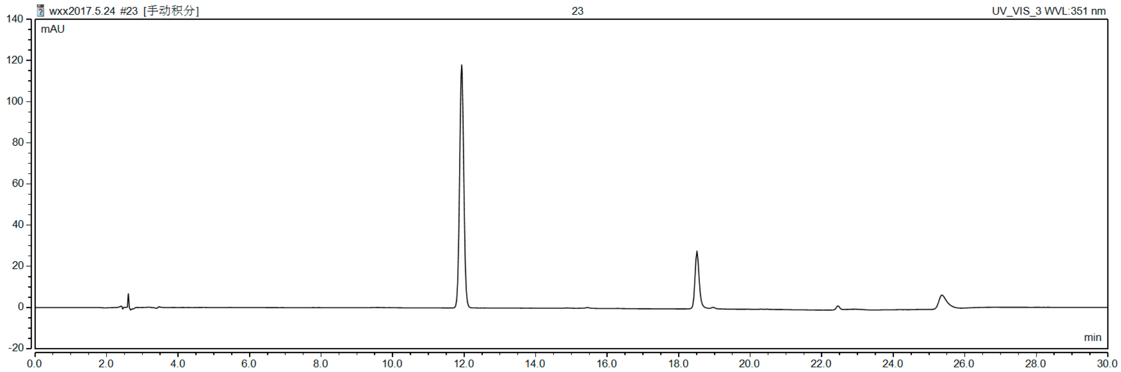
wxx2017.5.24 #23 [手动积分]
23
UV_VIS_3 WVL:351 nm
mAU
min

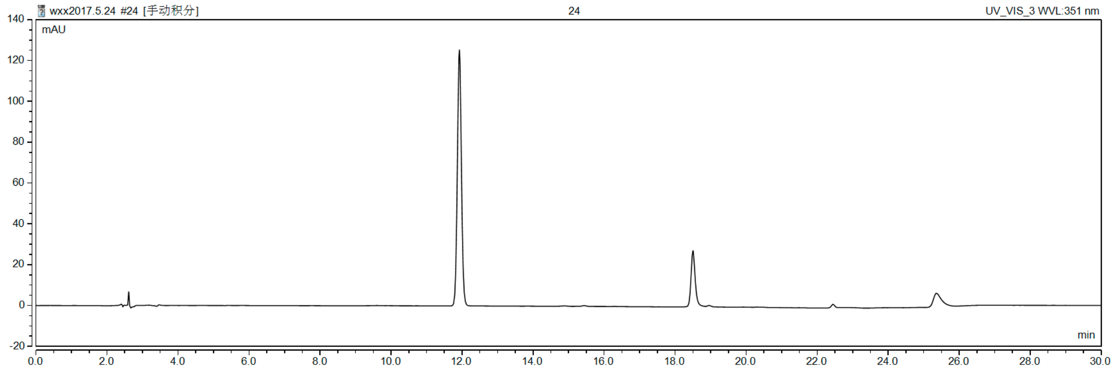
wxx2017.5.24 #24 [手动积分]
24
UV_VIS_3 WVL:351 nm
mAU
min

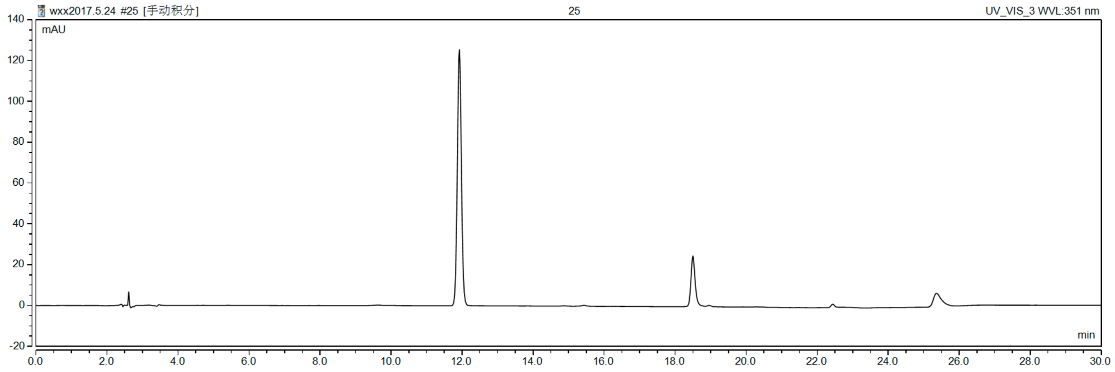
wxx2017.5.24 #25 [手动积分]
25
UV_VIS_3 WVL:351 nm
mAU
min

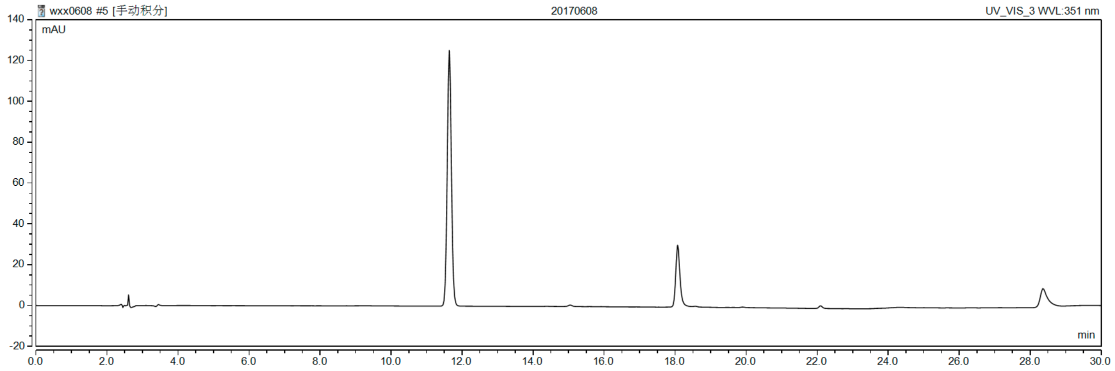
wxx0608 #5 [手动积分]
20170608
UV_VIS_3 WVL:351 nm
mAU
min

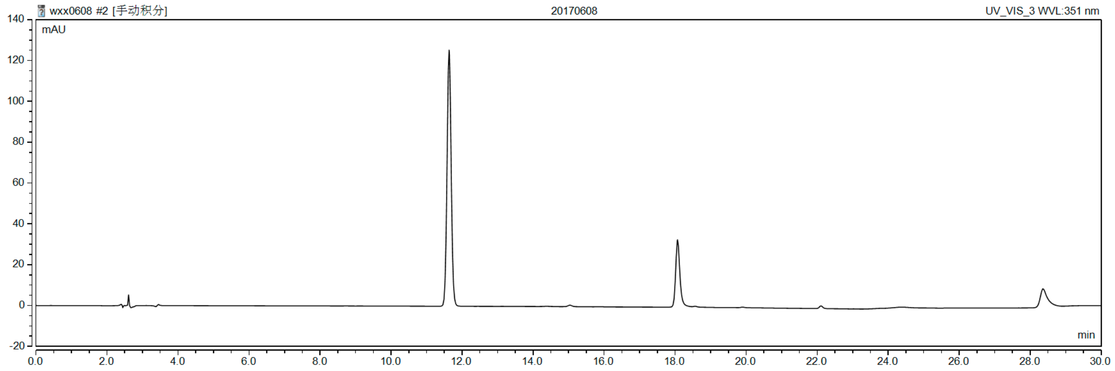
wxx0608 #2 [手动积分]
20170608
UV_VIS_3 WVL:351 nm
mAU
min

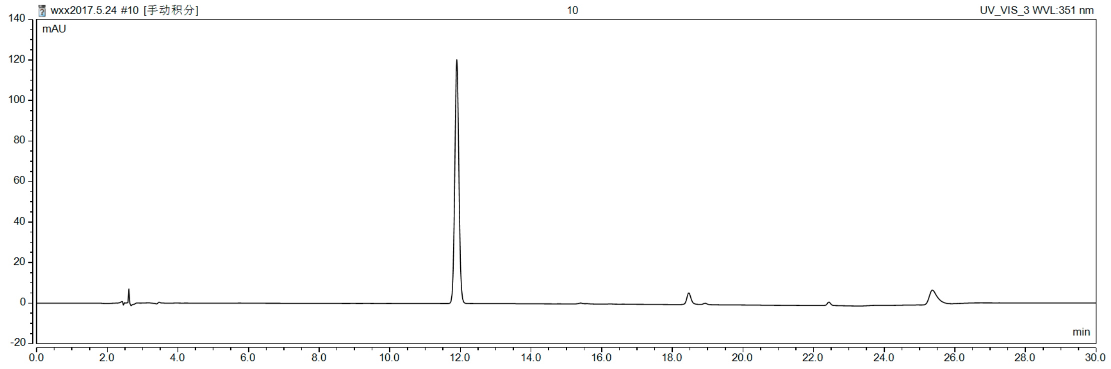
wxx2017.5.24 #10 [手动积分]
10
UV_VIS_3 WVL:351 nm
mAU
min

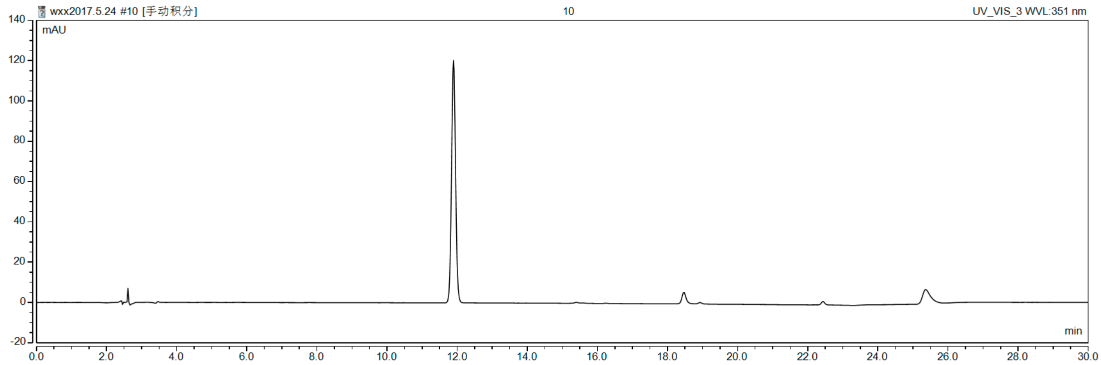
wxx2017.5.24 #10 [手动积分]
10
UV_VIS_3 WVL:351 nm
mAU
min

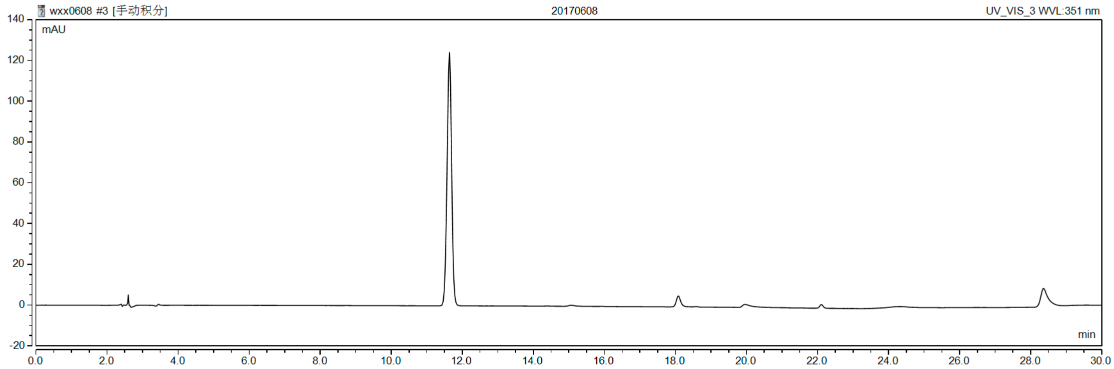
wxx0608 #3 [手动积分]
20170608
UV_VIS_3 WVL:351 nm
mAU
min

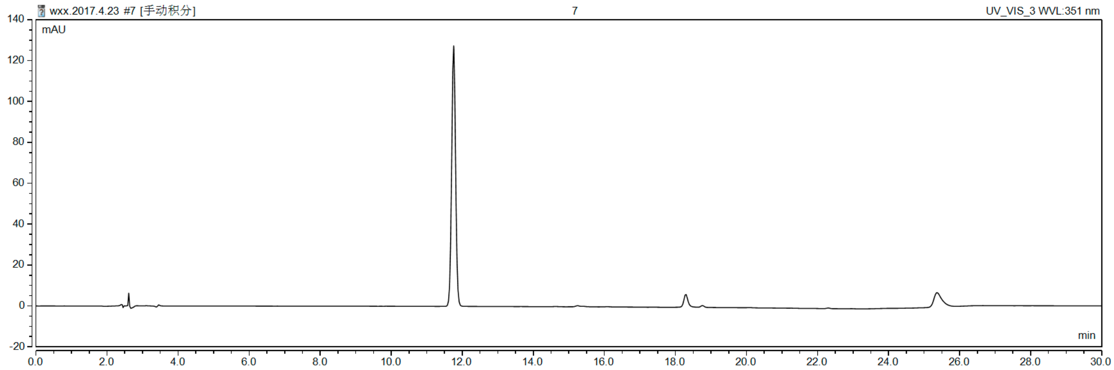
wxx.2017.4.23 #7 [手动积分]
7
UV_VIS_3 WVL:351 nm
mAU
min

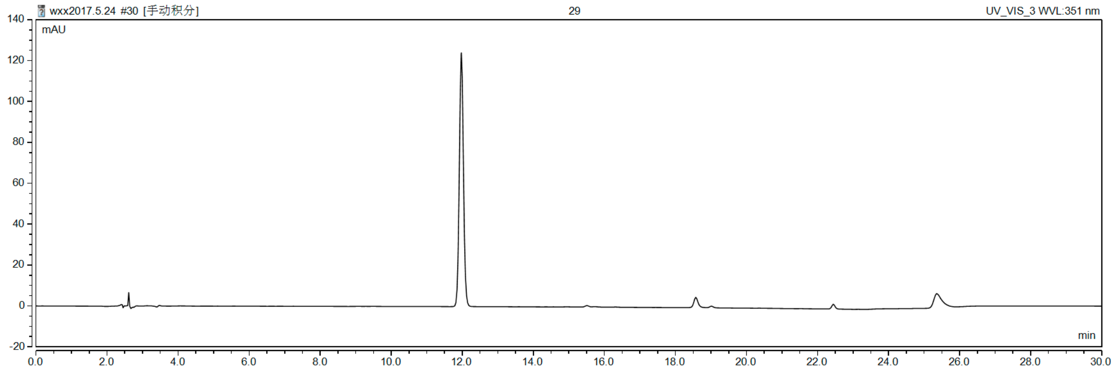
wxx2017.5.24 #30 [手动积分]
29
UV_VIS_3 WVL:351 nm
mAU
min

Stability

4℃

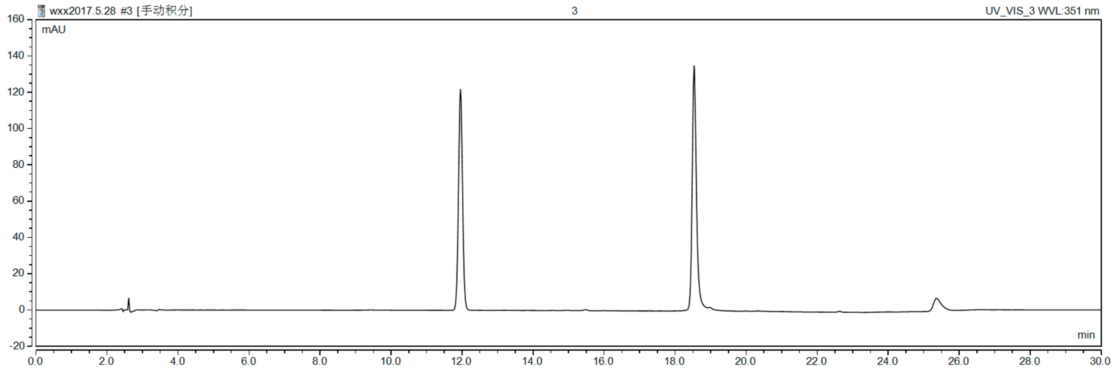
wxx2017.5.28 #3 [手动积分]
3
UV_VIS_3 WVL:351 nm
mAU
160
140
120
100
80
60
40
20
0
-20
min
0.0
2.0
4.0
6.0
8.0
10.0
12.0
14.0
16.0
18.0
20.0
22.0
24.0
26.0
28.0
30.0

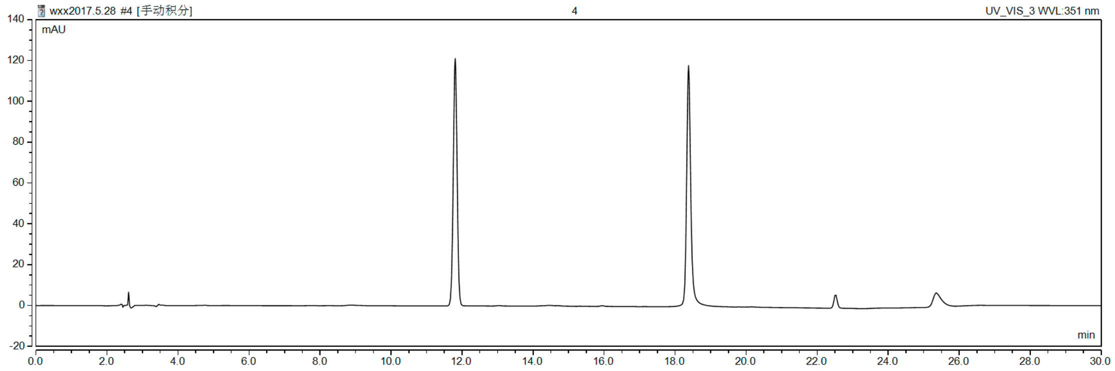
wxx2017.5.28 #4 [手动积分]
4
UV_VIS_3 WVL:351 nm
mAU
140
120
100
80
60
40
20
0
-20
min
0.0
2.0
4.0
6.0
8.0
10.0
12.0
14.0
16.0
18.0
20.0
22.0
24.0
26.0
28.0
30.0

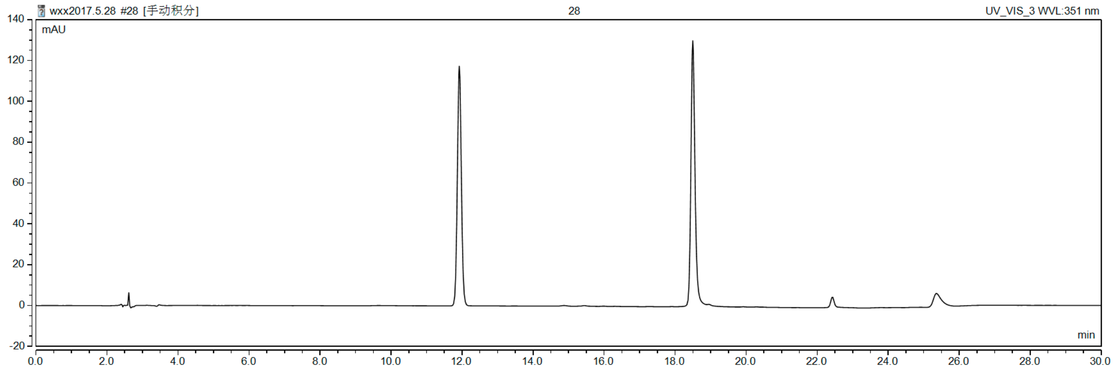
wxx2017.5.28 #28 [手动积分]
28
UV_VIS_3 WVL:351 nm
mAU
140
120
100
80
60
40
20
0
-20
min
0.0
2.0
4.0
6.0
8.0
10.0
12.0
14.0
16.0
18.0
20.0
22.0
24.0
26.0
28.0
30.0

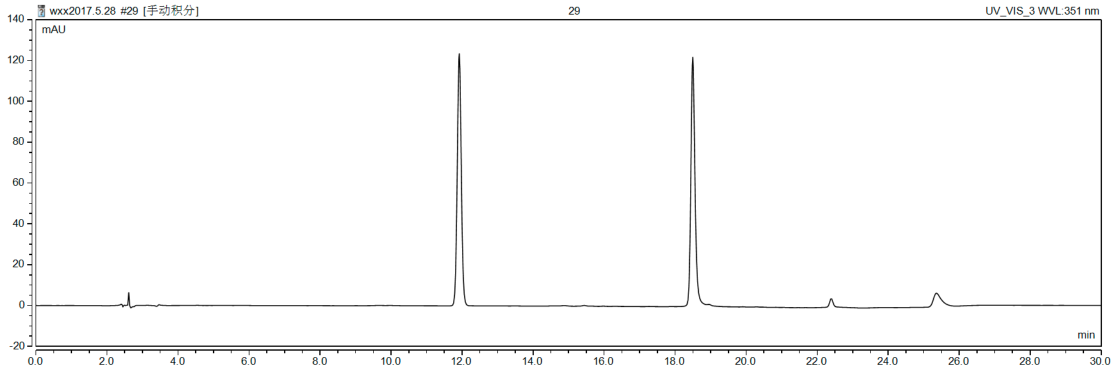
wxx2017.5.28 #29 [手动积分]
29
UV_VIS_3 WVL:351 nm
mAU
140
120
100
80
60
40
20
0
-20
min
0.0
2.0
4.0
6.0
8.0
10.0
12.0
14.0
16.0
18.0
20.0
22.0
24.0
26.0
28.0
30.0

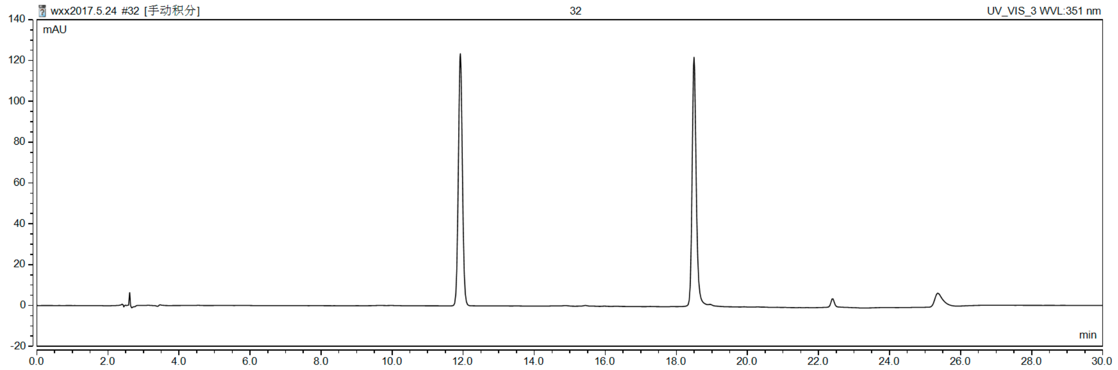
wxx2017.5.24 #32 [手动积分]
32
UV_VIS_3 WVL:351 nm
mAU
min

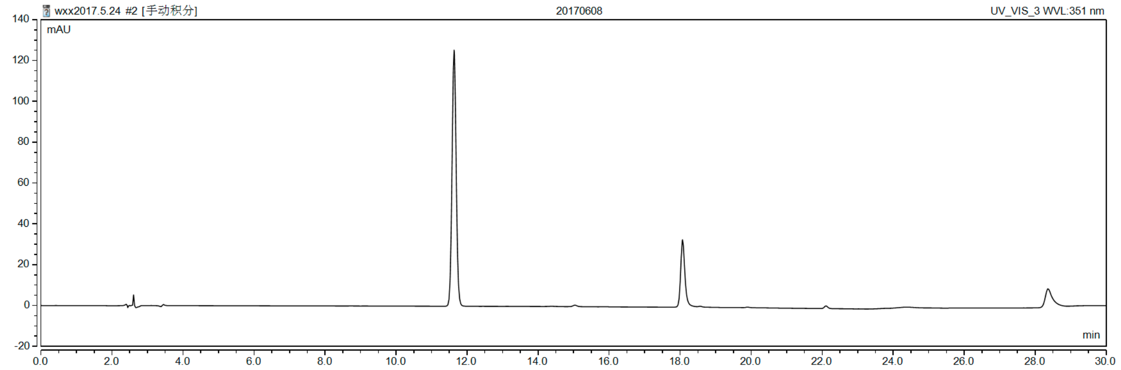
wxx2017.5.24 #2 [手动积分]
20170608
UV_VIS_3 WVL:351 nm
mAU
min

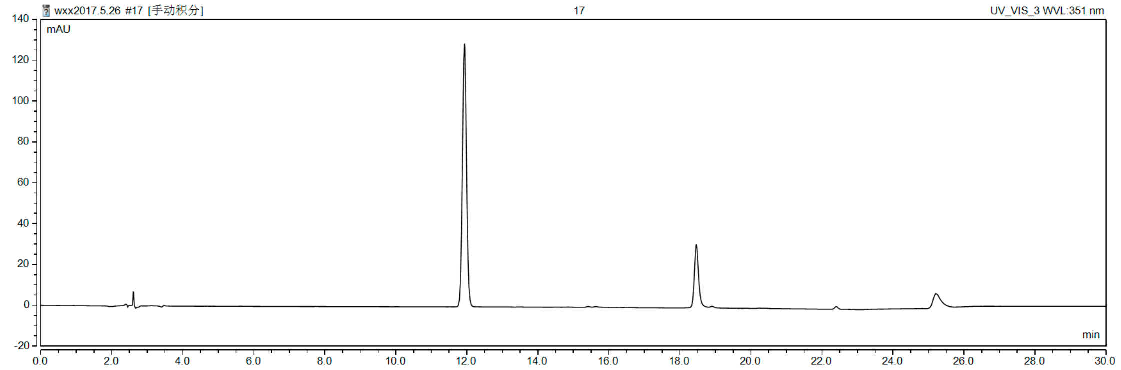
wxx2017.5.26 #17 [手动积分]
17
UV_VIS_3 WVL:351 nm
mAU
min

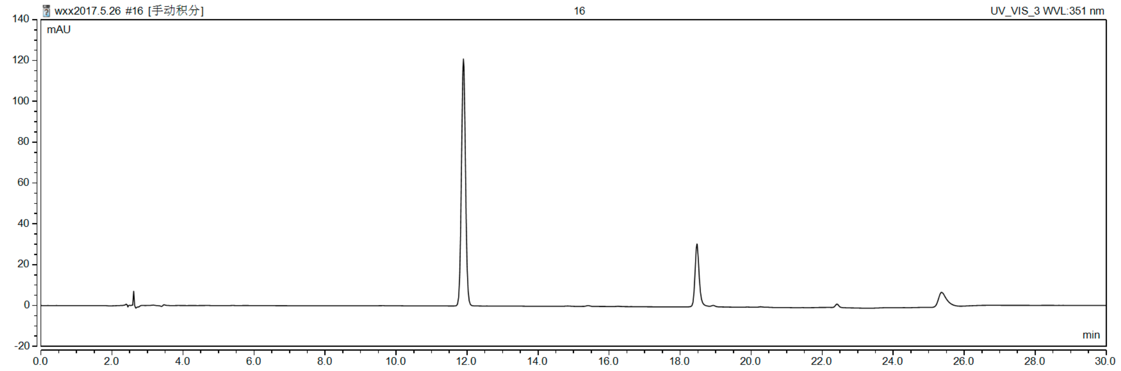
wxx2017.5.26 #16 [手动积分]
16
UV_VIS_3 WVL:351 nm
mAU
min

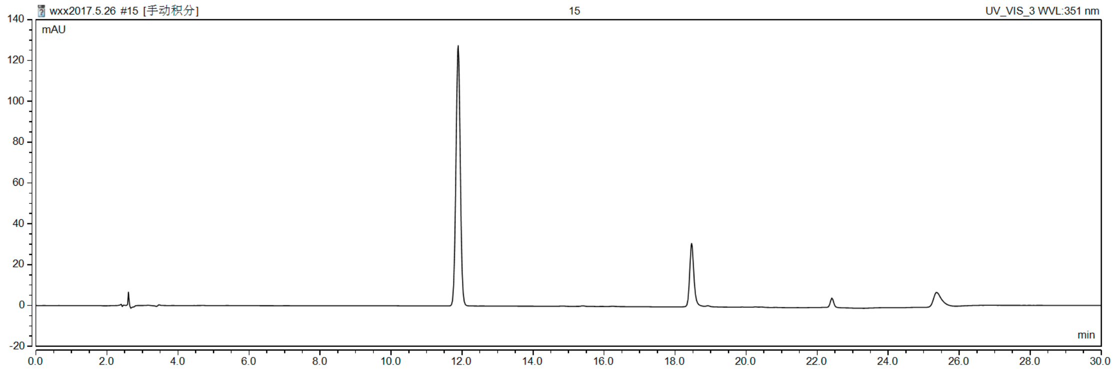
wxx2017.5.26 #15 [手动积分]
15
UV_VIS_3 WVL:351 nm
mAU
min

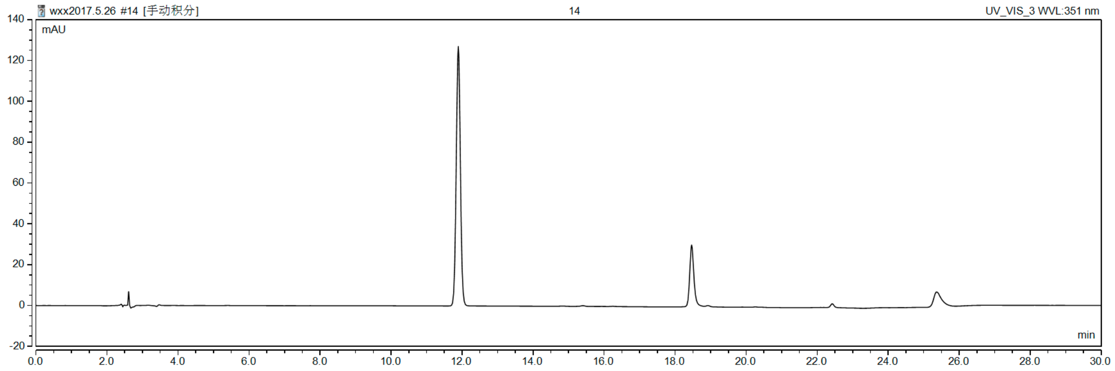
wxx2017.5.26 #14 [手动积分]
14
UV_VIS_3 WVL:351 nm
mAU
min

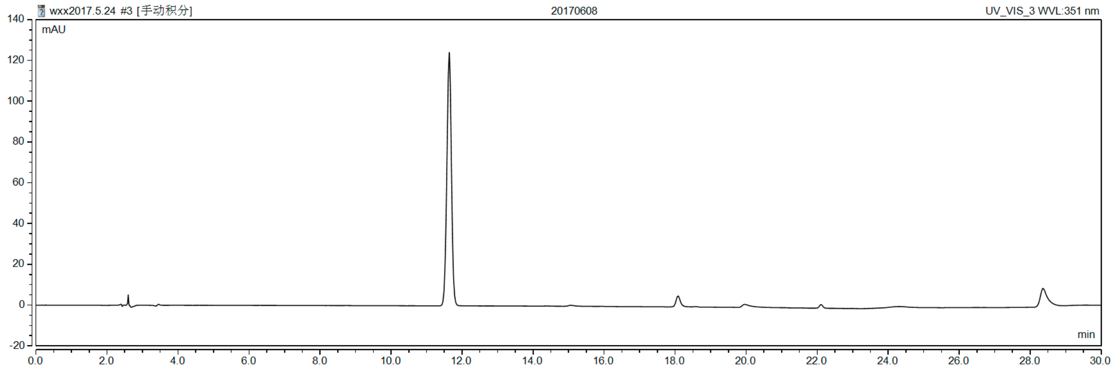
wxx2017.5.24 #3 [手动积分]
20170608
UV_VIS_3 WVL:351 nm
mAU
min

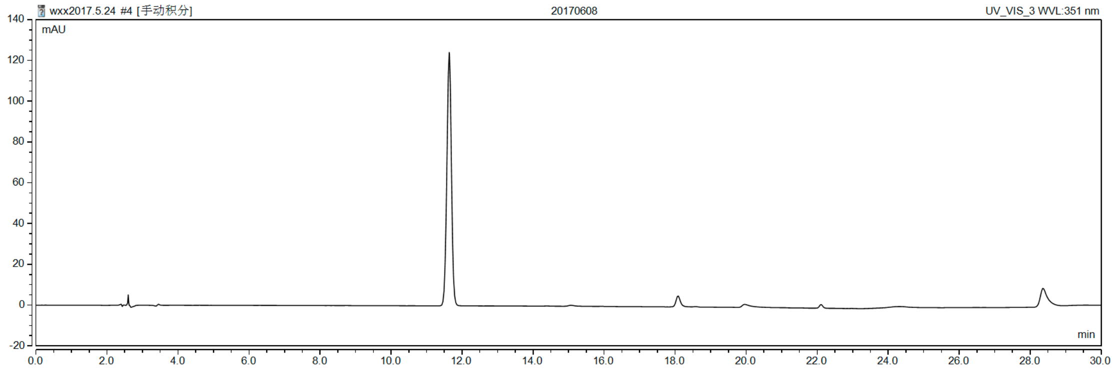
wxx2017.5.24 #4 [手动积分]
20170608
UV_VIS_3 WVL:351 nm
mAU
min

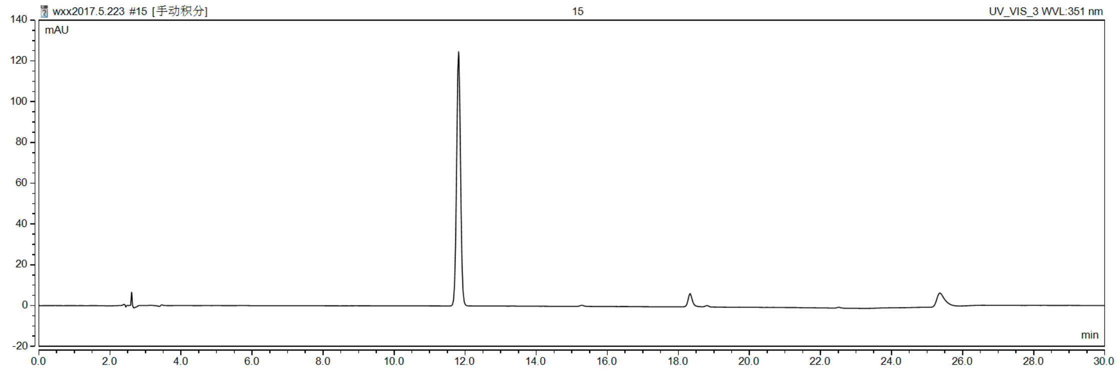
wxx2017.5.223 #15 [手动积分]
15
UV_VIS_3 WVL:351 nm
mAU
min

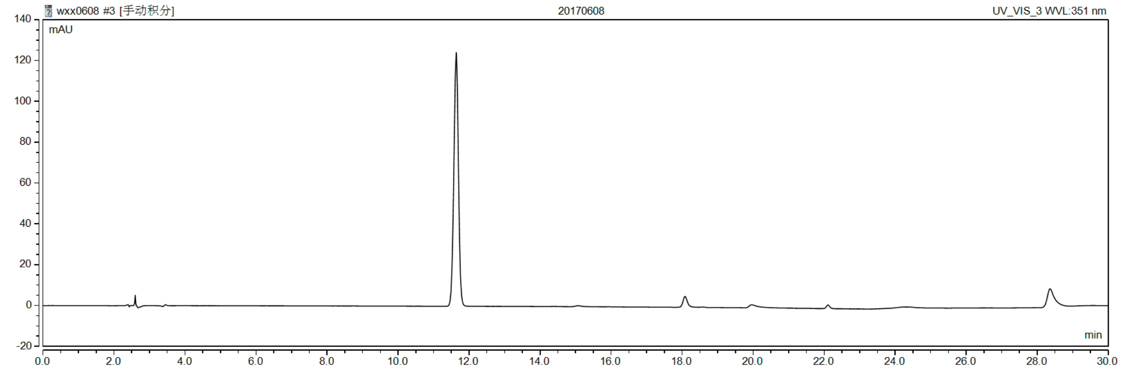
wxx0608 #3 [手动积分]
20170608
UV_VIS_3 WVL:351 nm
mAU
min

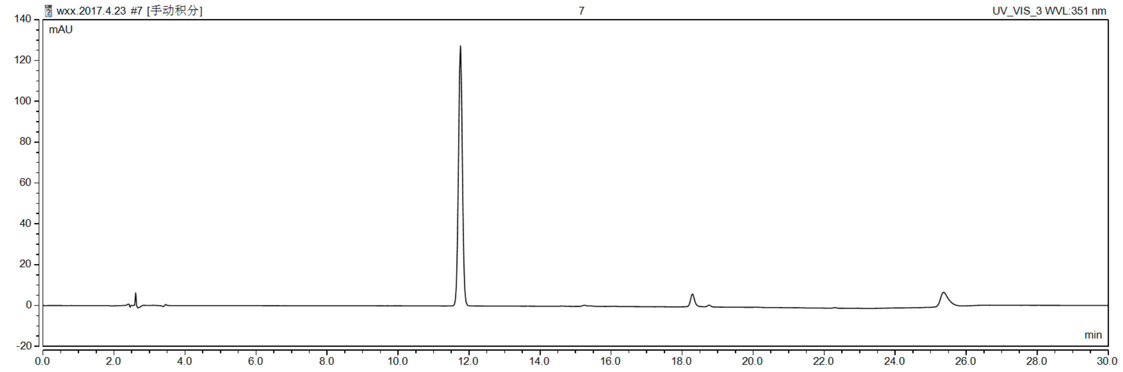
wxx.2017.4.23 #7 [手动积分]
7
UV_VIS_3 WVL:351 nm
mAU
min

-20℃

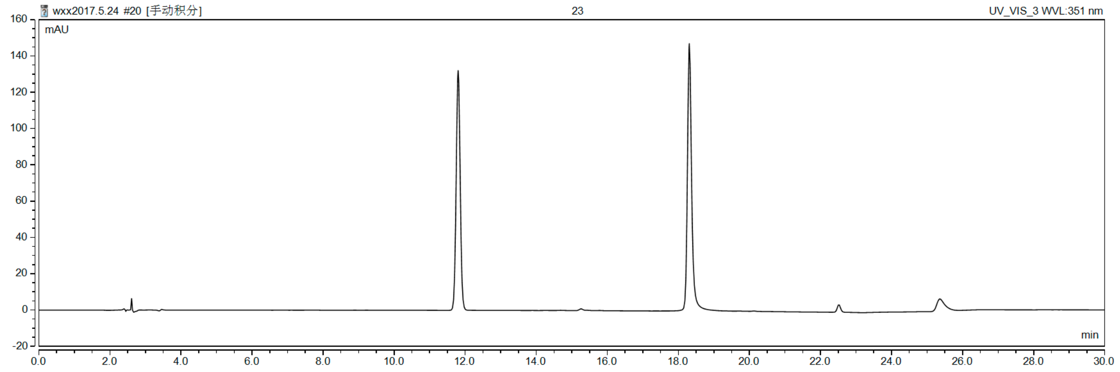
wxx2017.5.24 #20 [手动积分]
23
UV_VIS_3 WVL:351 nm
mAU
min

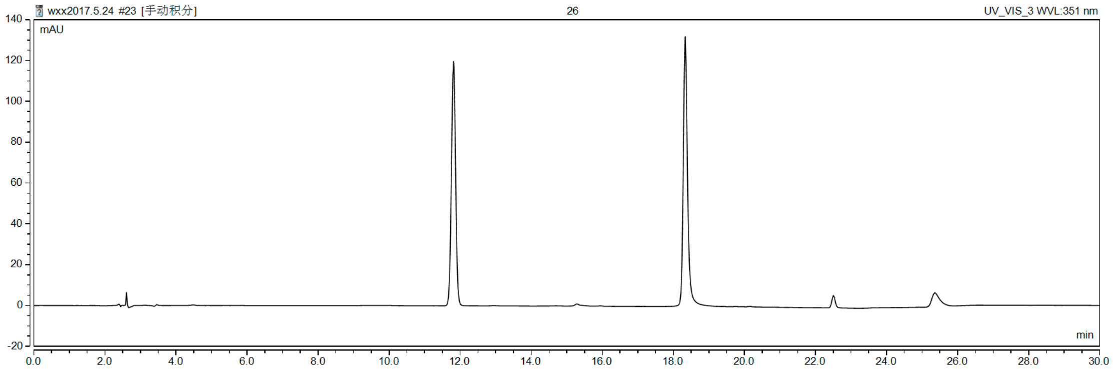
wxx2017.5.24 #23 [手动积分]
26
UV_VIS_3 WVL:351 nm
mAU
min

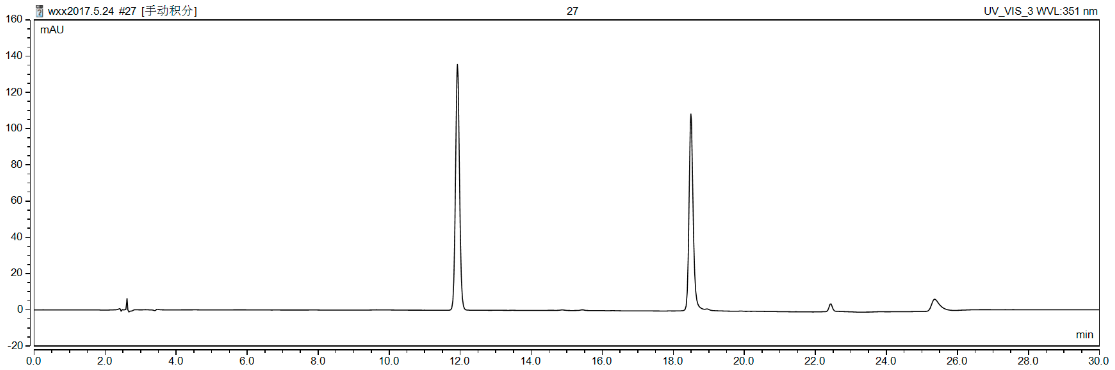
wxx2017.5.24 #27 [手动积分]
27
UV_VIS_3 WVL:351 nm
mAU
min

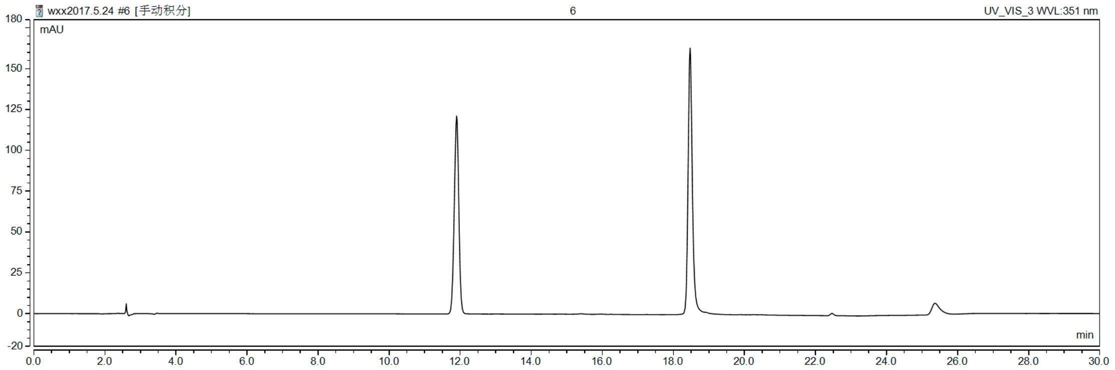
wxx2017.5.24 #6 [手动积分]
6
UV_VIS_3 WVL:351 nm
mAU
min

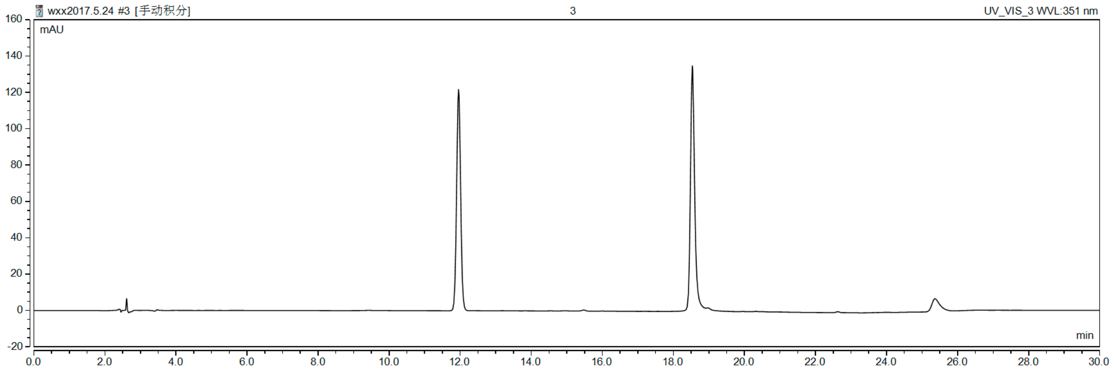
wxx2017.5.24 #3 [手动积分]
3
UV_VIS_3 WVL:351 nm
mAU
min

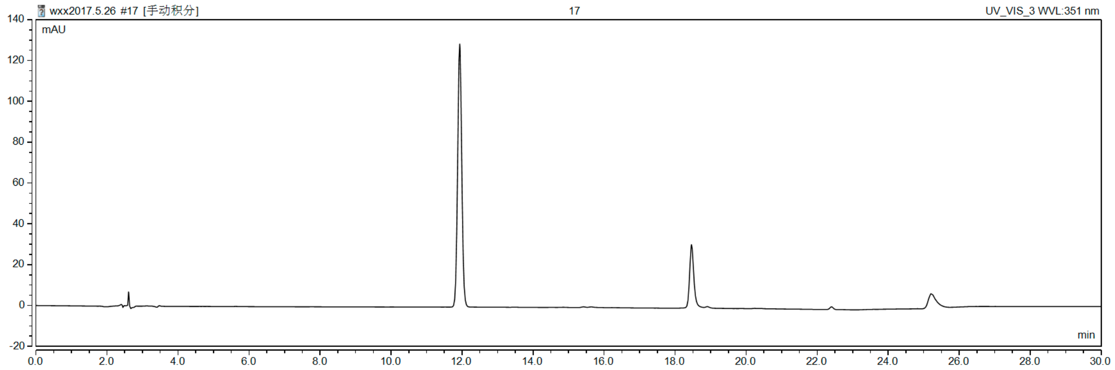
wxx2017.5.26 #17 [手动积分]
17
UV_VIS_3 WVL:351 nm
mAU
min

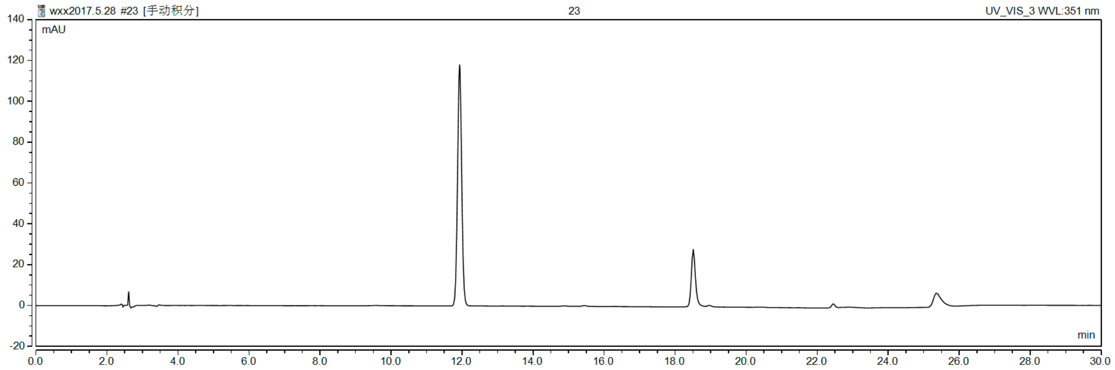
wxx2017.5.28 #23 [手动积分]
23
UV_VIS_3 WVL:351 nm
mAU
min

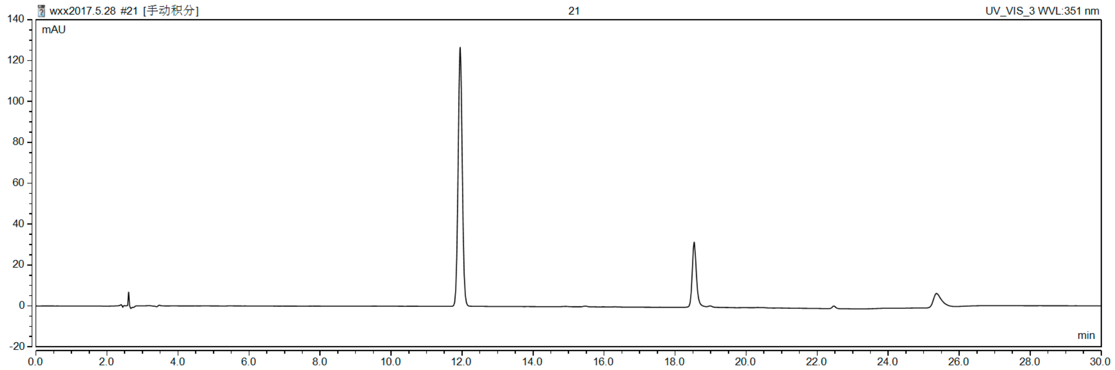
wxx2017.5.28 #21 [手动积分]
21
UV_VIS_3 WVL:351 nm
mAU
min

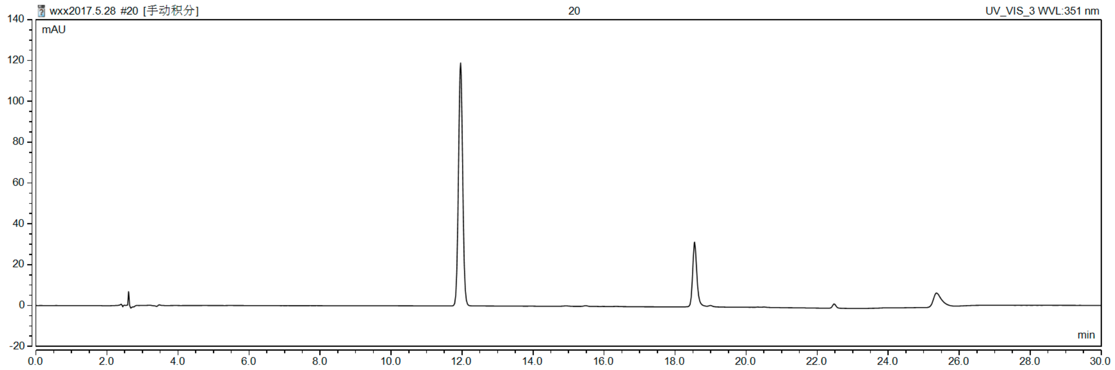
wxx2017.5.28 #20 [手动积分]
20
UV_VIS_3 WVL:351 nm
mAU
min

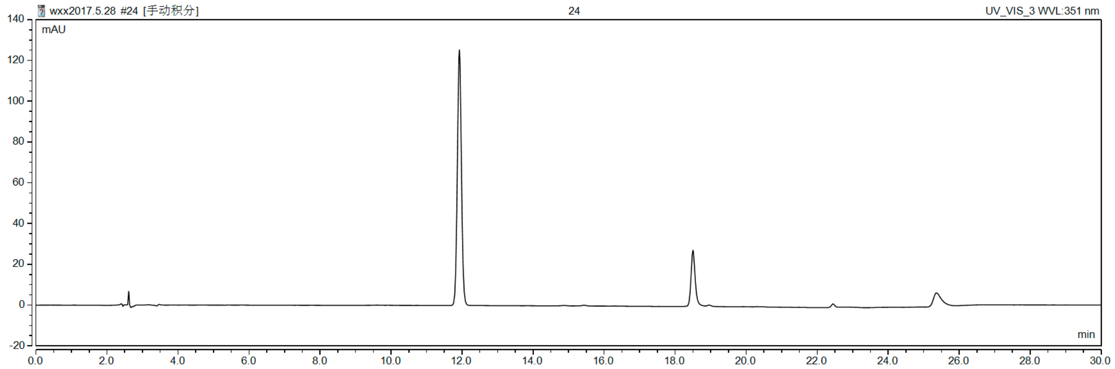
wxx2017.5.28 #24 [手动积分]
24
UV_VIS_3 WVL:351 nm
mAU
min

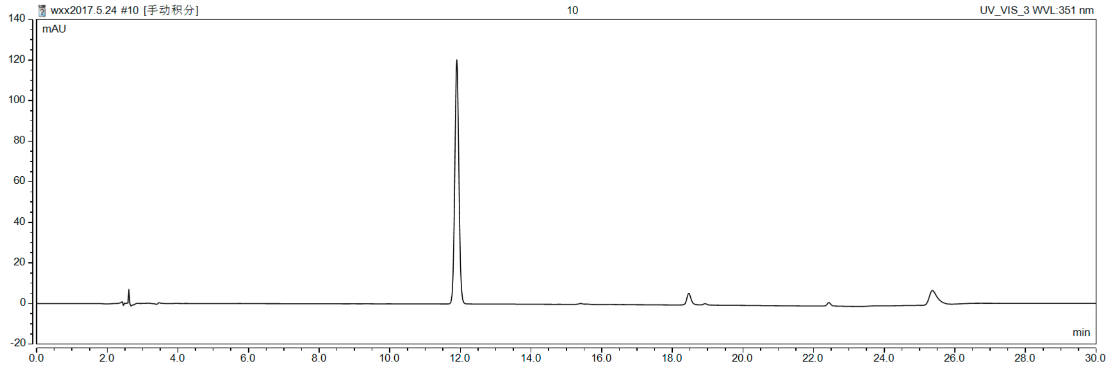
wxx2017.5.24 #10 [手动积分]
10
UV_VIS_3 WVL:351 nm
mAU
min

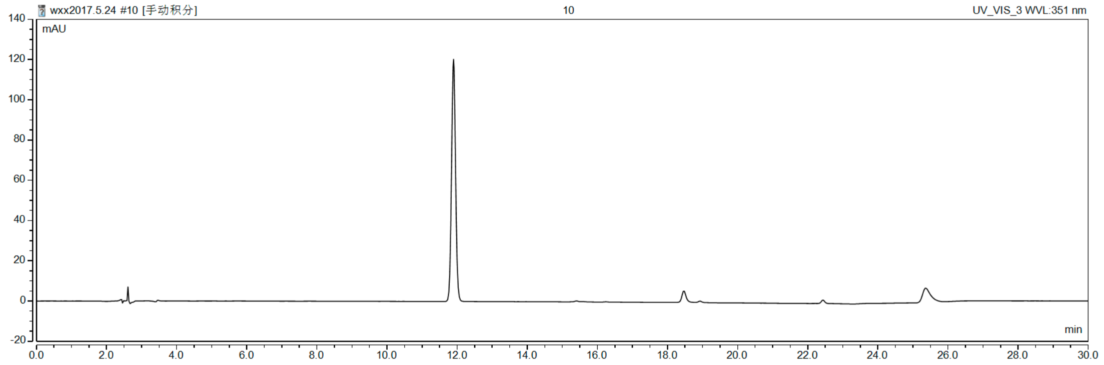
wxx2017.5.24 #10 [手动积分]
10
UV_VIS_3 WVL:351 nm
mAU
min

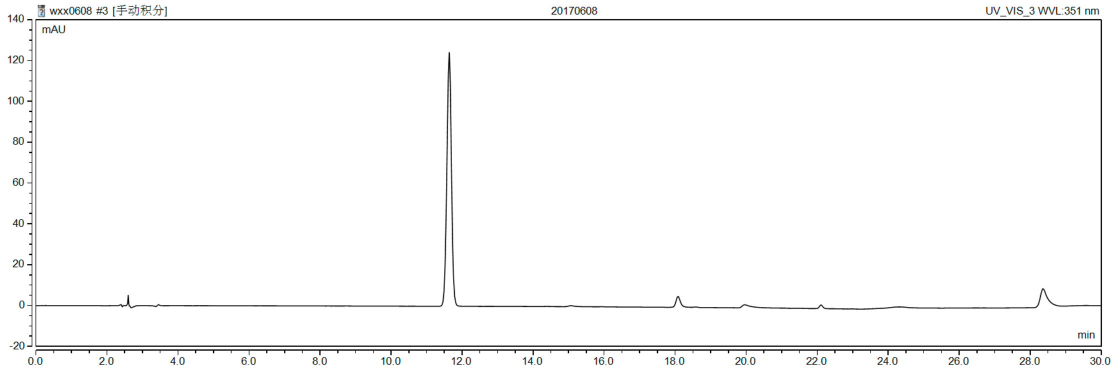
wxx0608 #3 [手动积分]
20170608
UV_VIS_3 WVL:351 nm
mAU
min

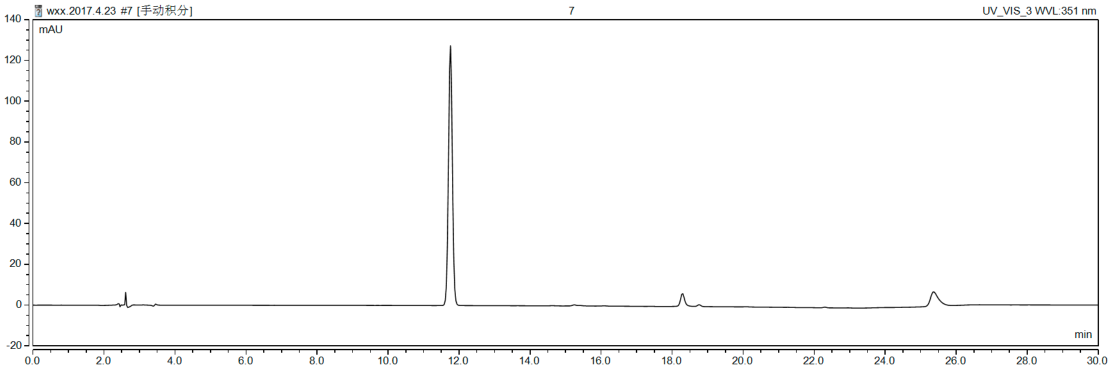
wxx.2017.4.23 #7 [手动积分]
7
UV_VIS_3 WVL:351 nm
mAU
min

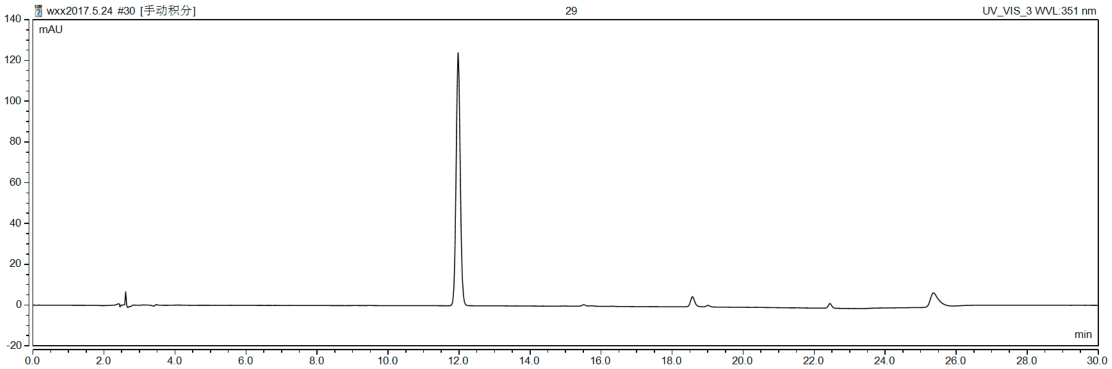
wxx2017.5.24 #30 [手动积分]
29
UV_VIS_3 WVL:351 nm
mAU
min

Six Rats

one

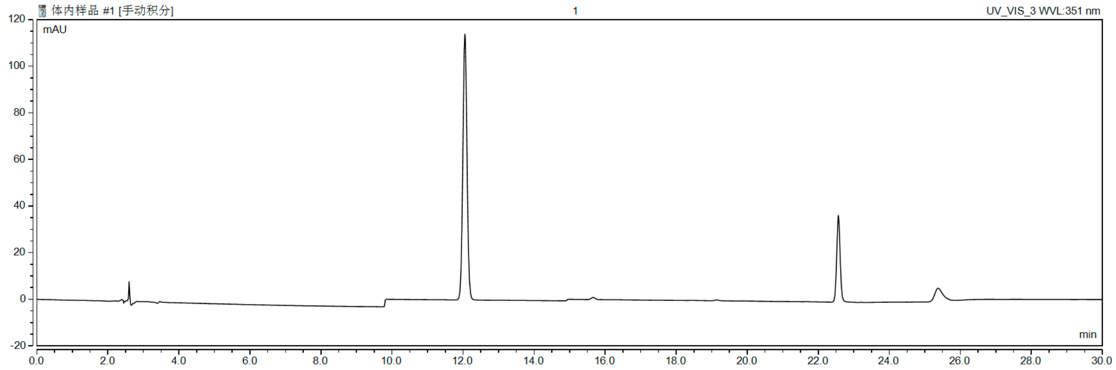

体内样品 #1 [手动积分]
1
UV_VIS_3 WVL:351 nm
mAU
min

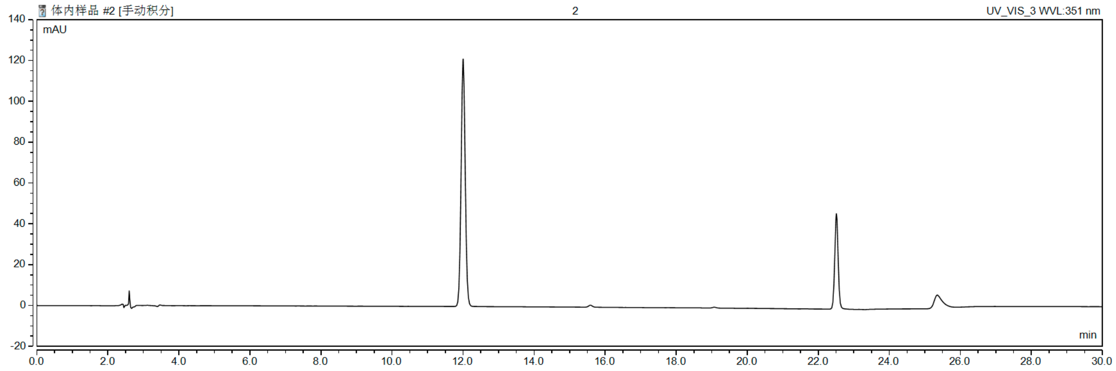

体内样品 #2 [手动积分]
2
UV_VIS_3 WVL:351 nm
mAU
min

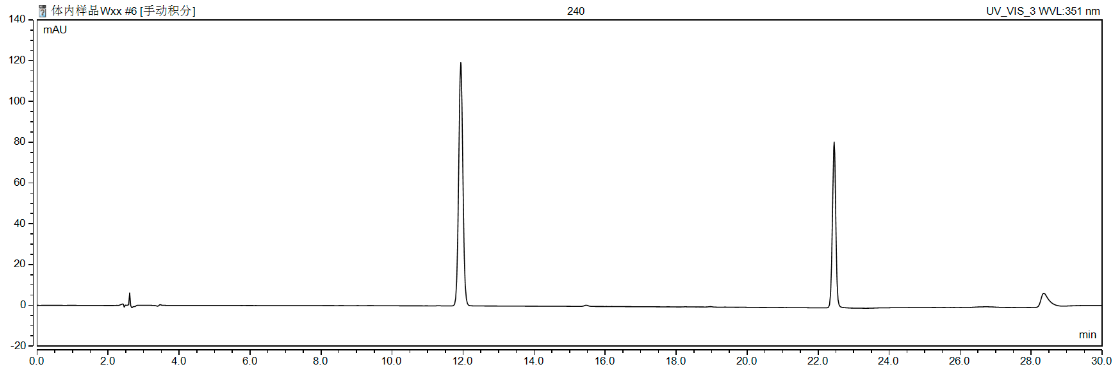

体内样品Wxx #6 [手动积分]
240
UV_VIS_3 WVL:351 nm
mAU
min

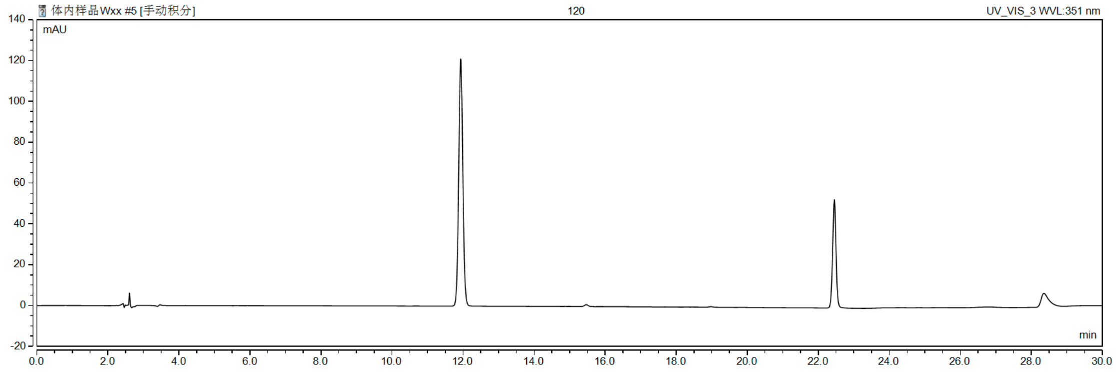

体内样品Wxx #5 [手动积分]
120
UV_VIS_3 WVL:351 nm
mAU
min

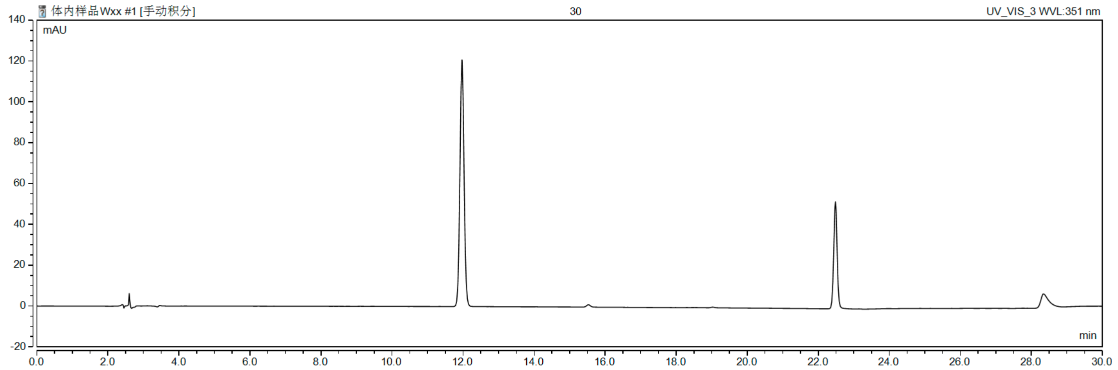
体内样品Wxx #1 [手动积分]
30
UV_VIS_3 WVL:351 nm
mAU
min

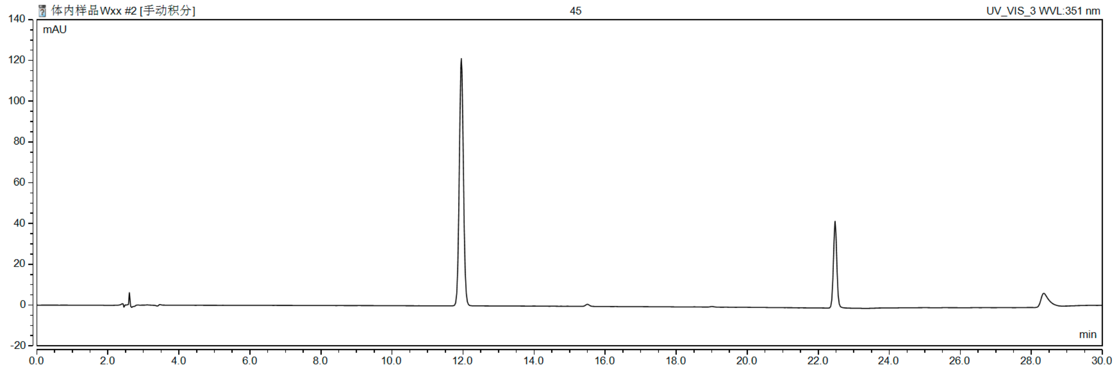
体内样品Wxx #2 [手动积分]
45
UV_VIS_3 WVL:351 nm
mAU
min

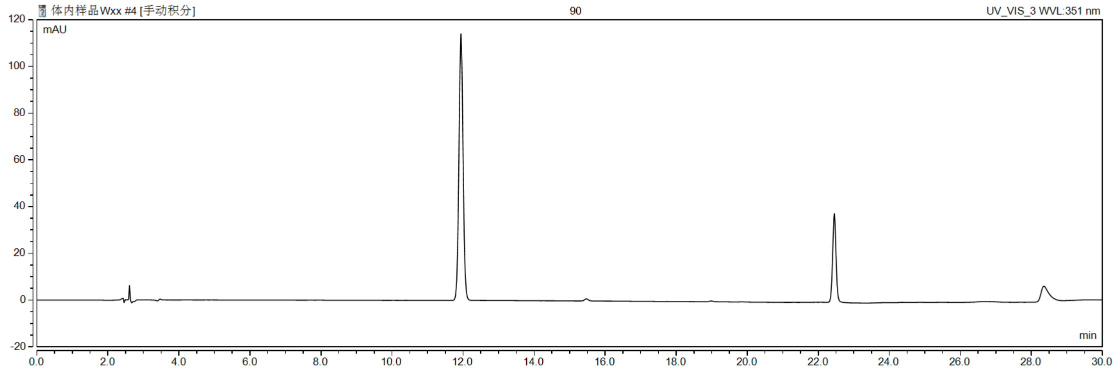
体内样品Wxx #4 [手动积分]
90
UV_VIS_3 WVL:351 nm
mAU
min

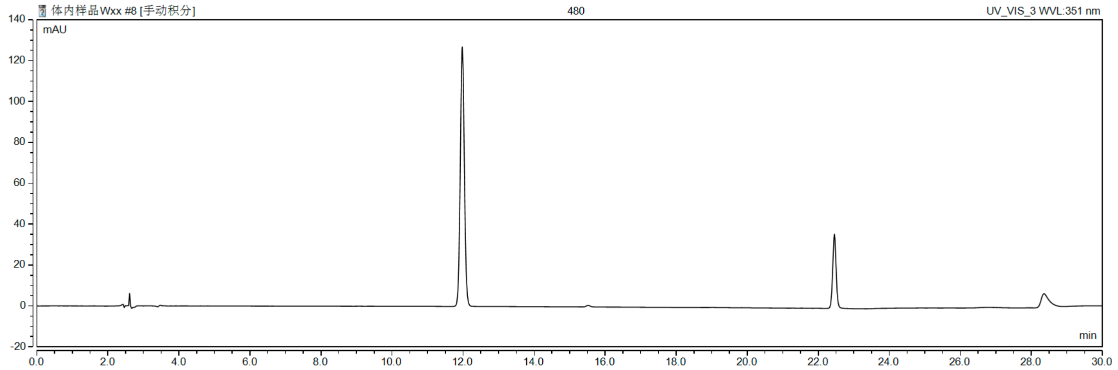
体内样品Wxx #8 [手动积分]
480
UV_VIS_3 WVL:351 nm
mAU
min

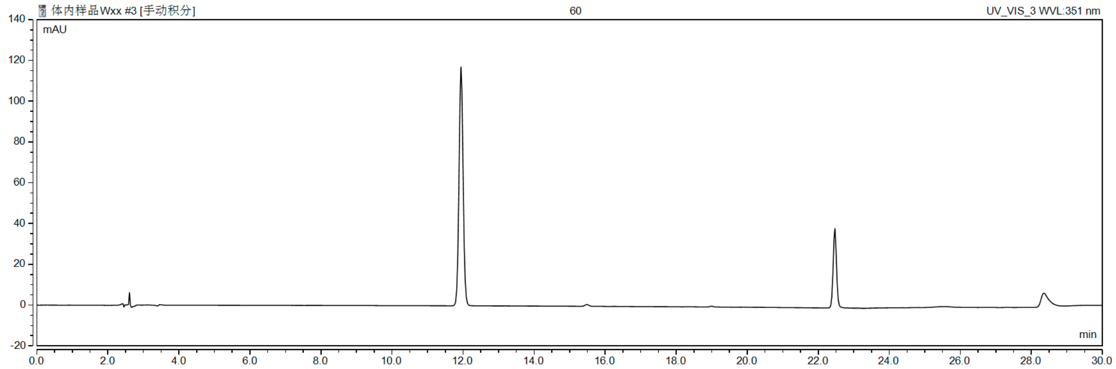
体内样品Wxx #3 [手动积分]
60
UV_VIS_3 WVL:351 nm
mAU
min

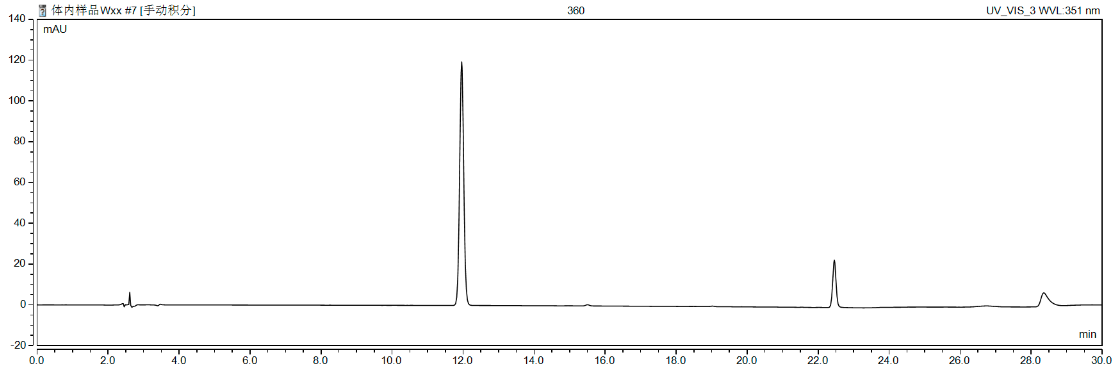
体内样品Wxx #7 [手动积分]
360
UV_VIS_3 WVL:351 nm
mAU
min

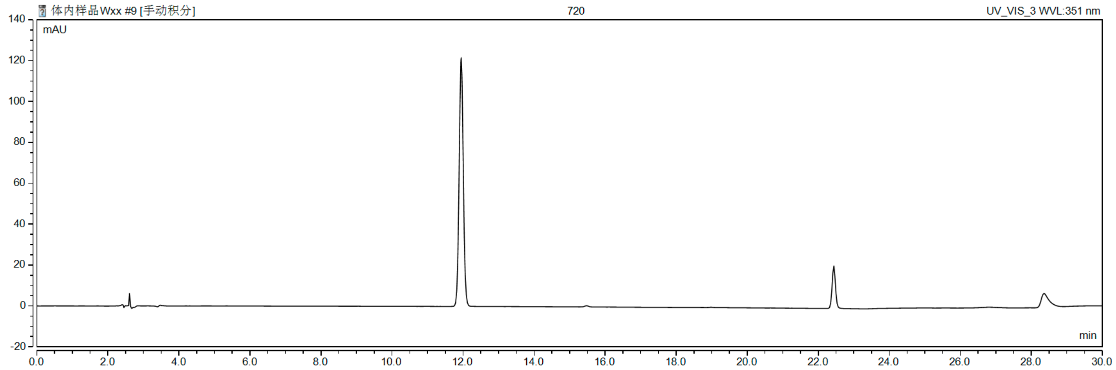
体内样品Wxx #9 [手动积分]
720
UV_VIS_3 WVL:351 nm
mAU
min

two

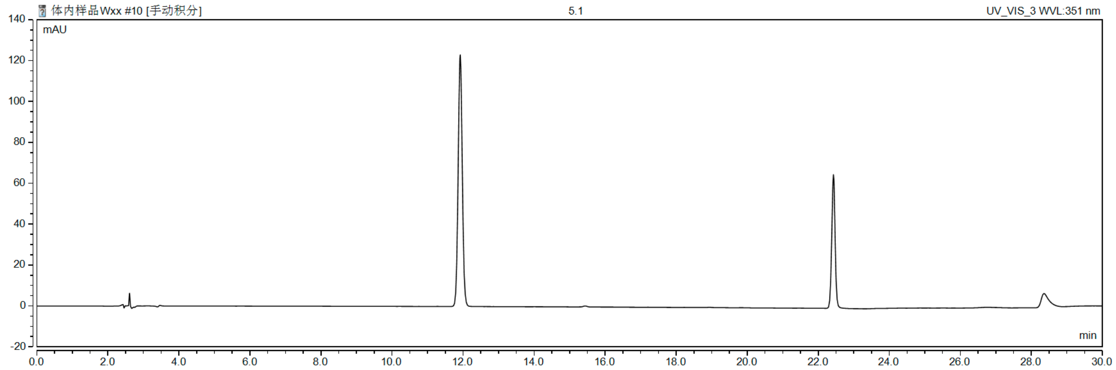
体内样品Wxx #10 [手动积分]
5.1
UV_VIS_3 WVL:351 nm
mAU
min

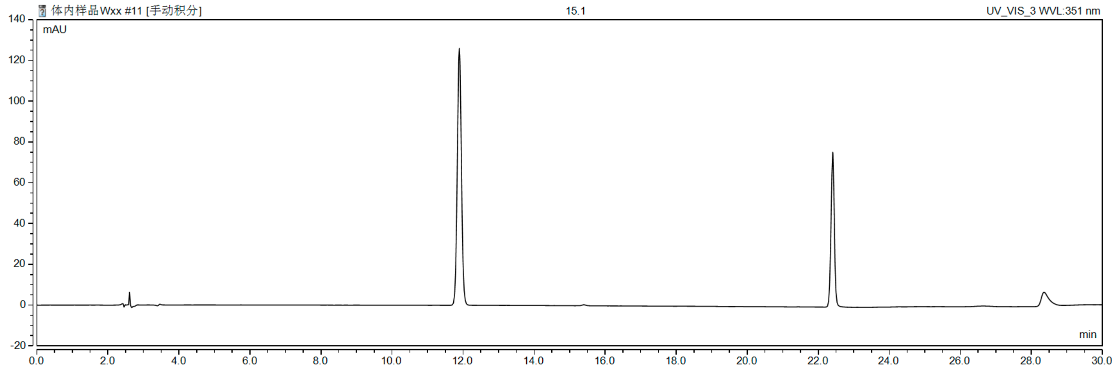
体内样品Wxx #11 [手动积分]
15.1
UV_VIS_3 WVL:351 nm
mAU
min

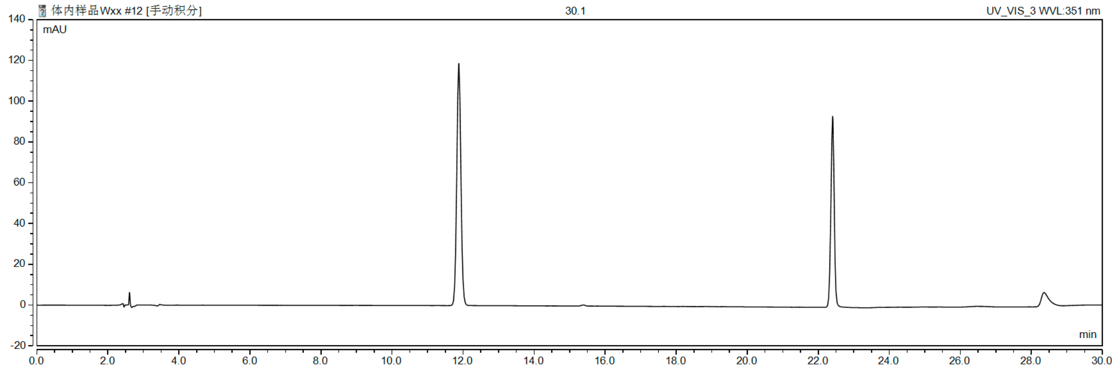
体内样品Wxx #12 [手动积分]
30.1
UV_VIS_3 WVL:351 nm
mAU
min

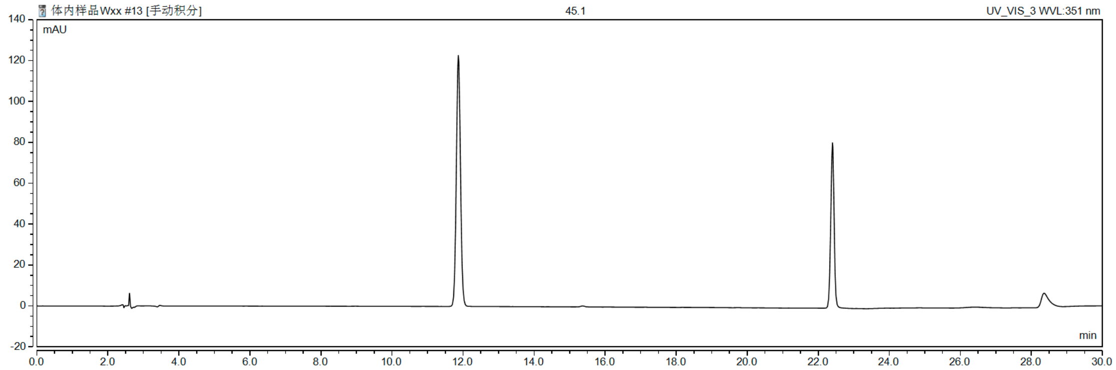
体内样品Wxx #13 [手动积分]
45.1
UV_VIS_3 WVL:351 nm
mAU
min

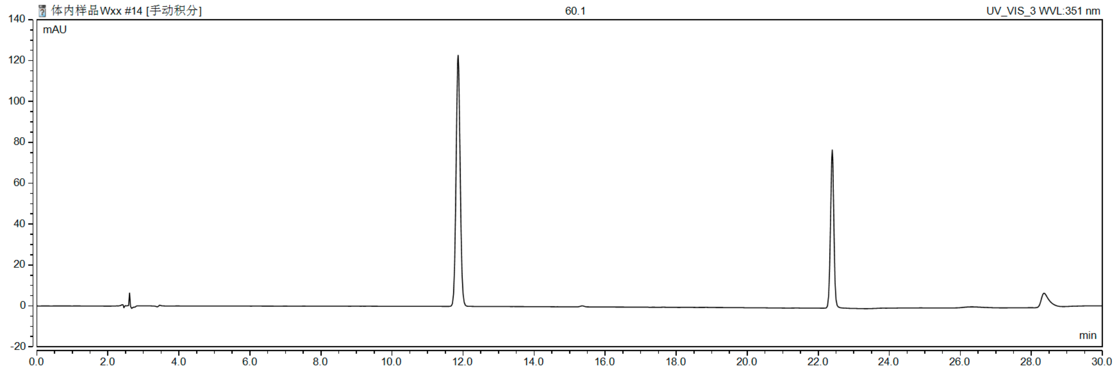
体内样品Wxx #14 [手动积分]
60.1
UV_VIS_3 WVL:351 nm
mAU
min

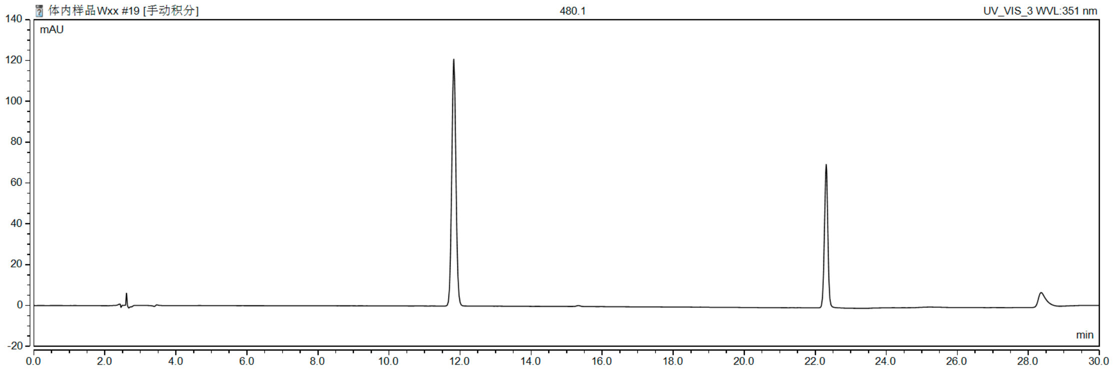
体内样品Wxx #19 [手动积分]
480.1
UV_VIS_3 WVL:351 nm
mAU
min

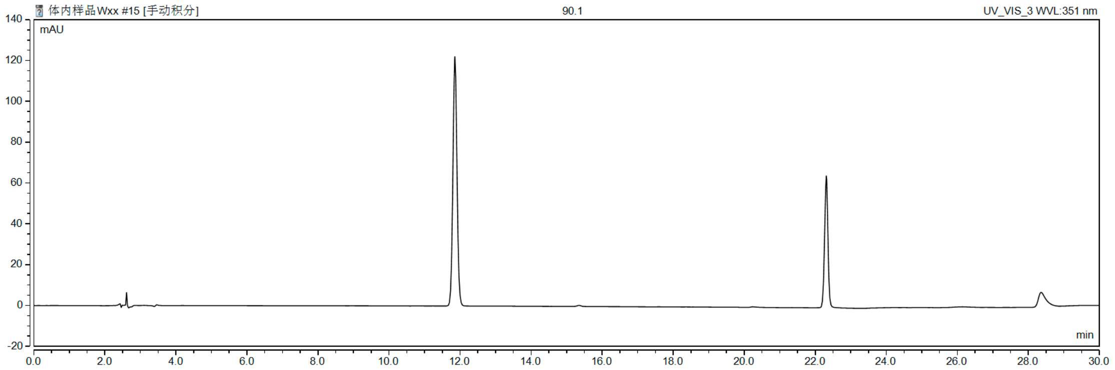
体内样品Wxx #15 [手动积分]
90.1
UV_VIS_3 WVL:351 nm
mAU
min

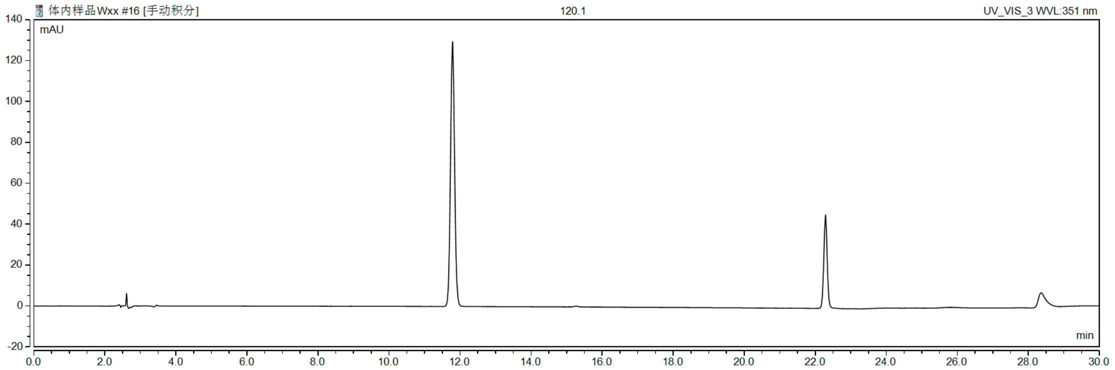
体内样品Wxx #16 [手动积分]
120.1
UV_VIS_3 WVL:351 nm
mAU
min

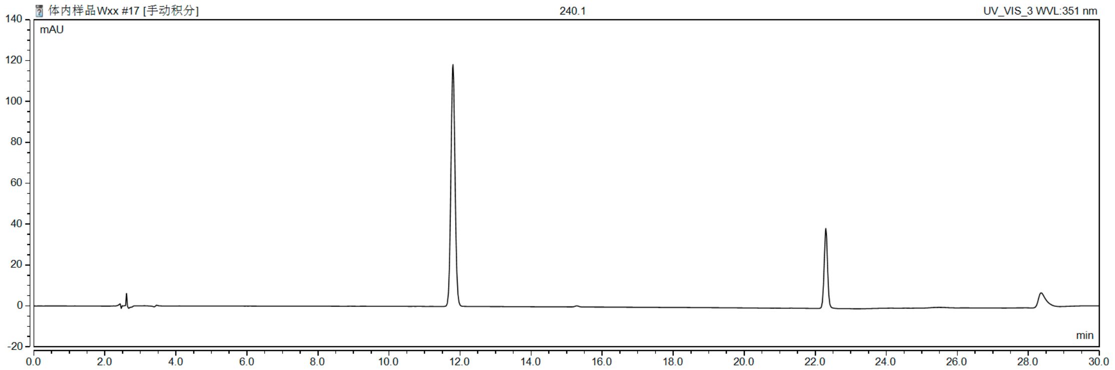
体内样品Wxx #17 [手动积分]
240.1
UV_VIS_3 WVL:351 nm
mAU
min

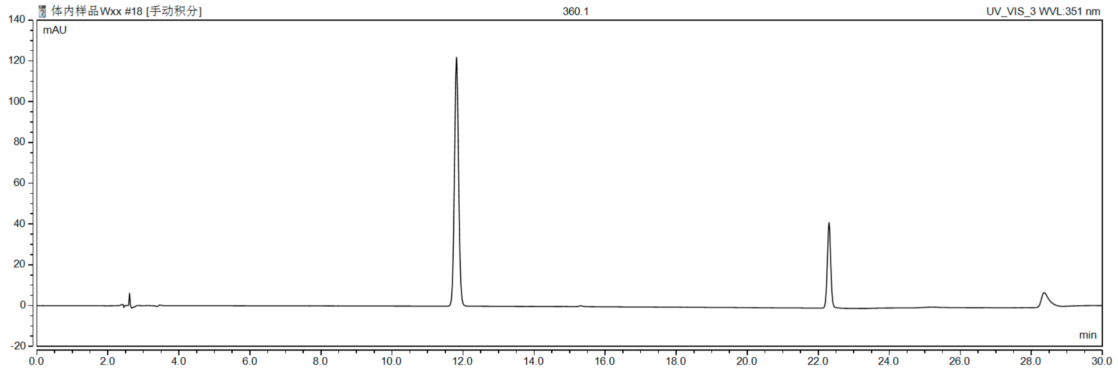
体内样品Wxx #18 [手动积分]
360.1
UV_VIS_3 WVL:351 nm
mAU
min

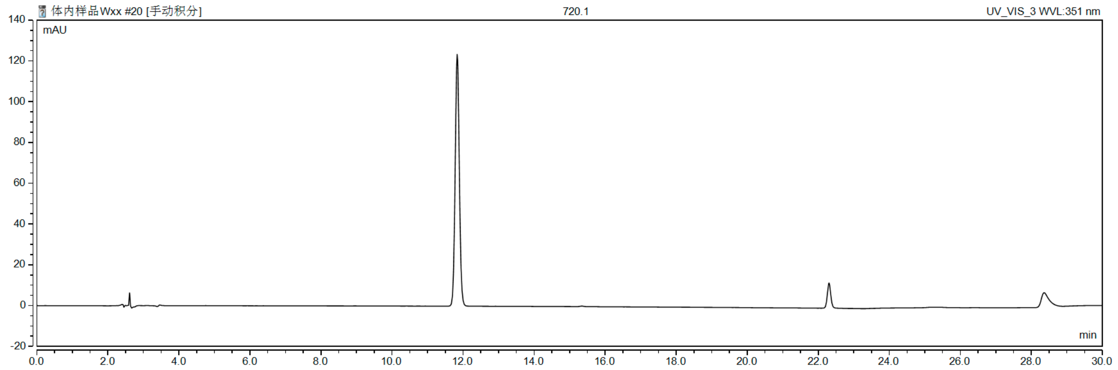
体内样品Wxx #20 [手动积分]
720.1
UV_VIS_3 WVL:351 nm
mAU
min

three

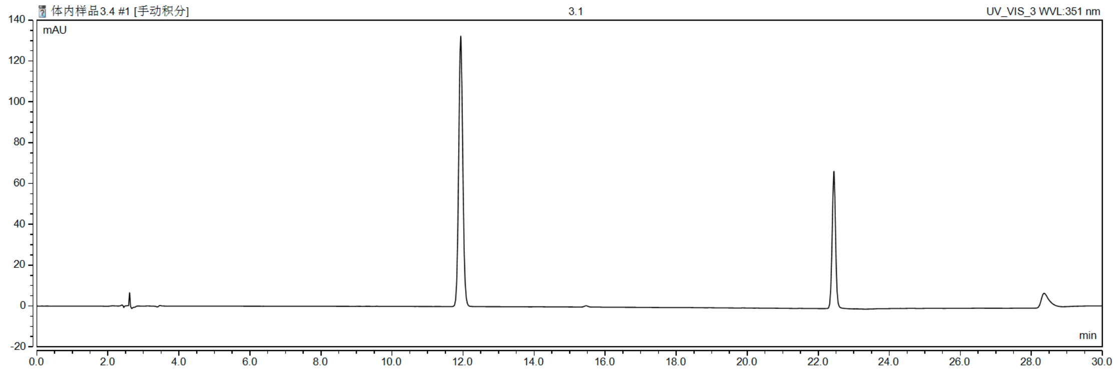
体内样品3.4 #1 [手动积分]
3.1
UV_VIS_3 WVL:351 nm
mAU
min

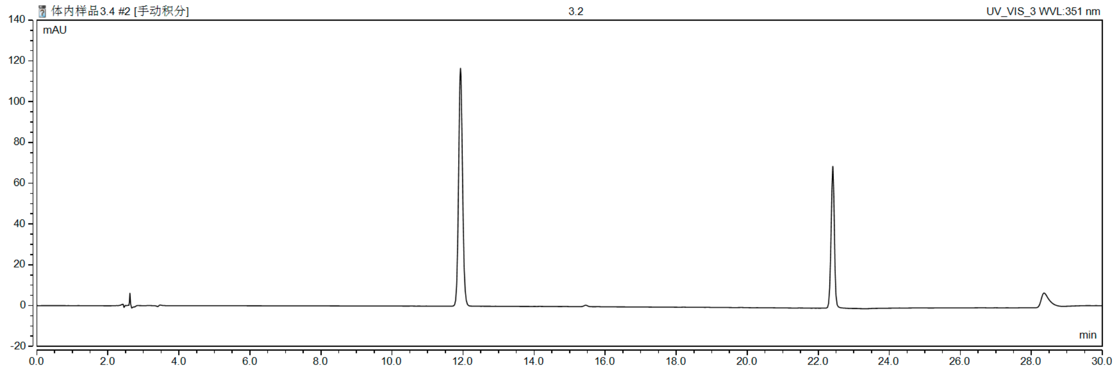
体内样品3.4 #2 [手动积分]
3.2
UV_VIS_3 WVL:351 nm
mAU
min

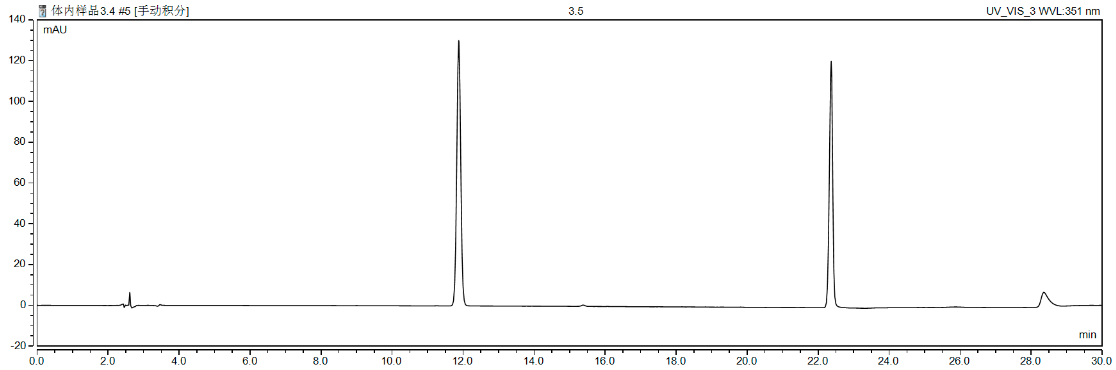

体内样品3.4 #5 [手动积分]
3.5
UV_VIS_3 WVL:351 nm
mAU
min

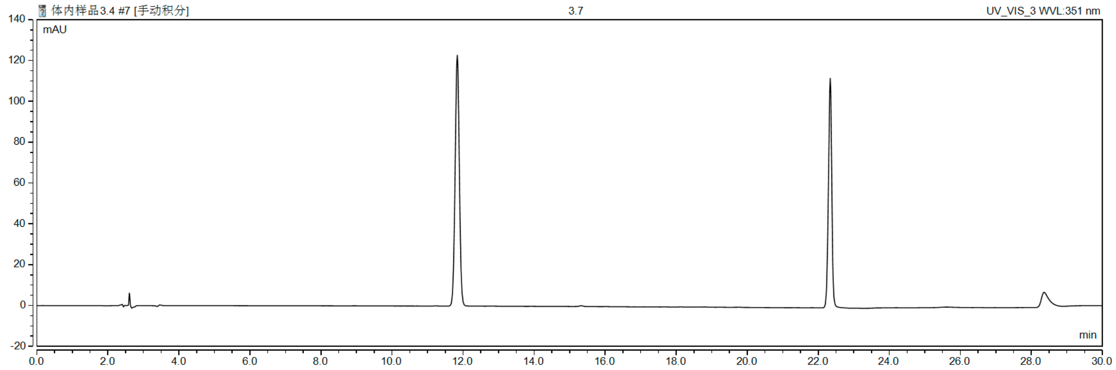

体内样品3.4 #7 [手动积分]
3.7
UV_VIS_3 WVL:351 nm
mAU
min

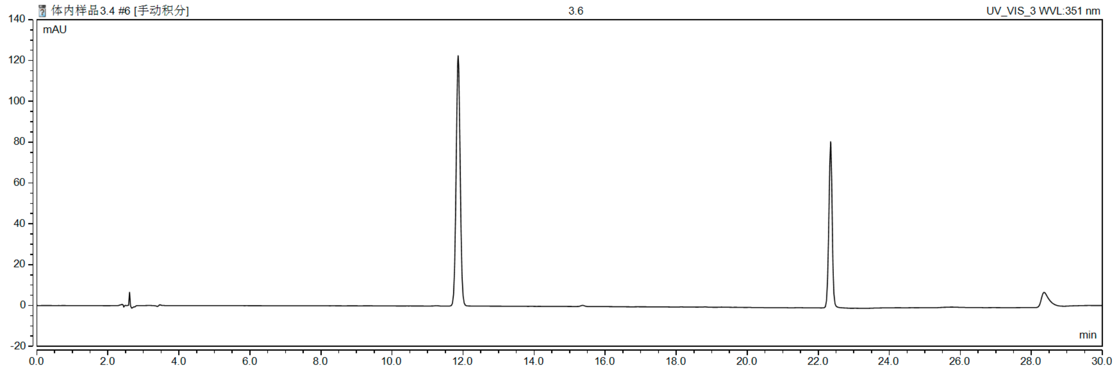

体内样品3.4 #6 [手动积分]
3.6
UV_VIS_3 WVL:351 nm
mAU
min

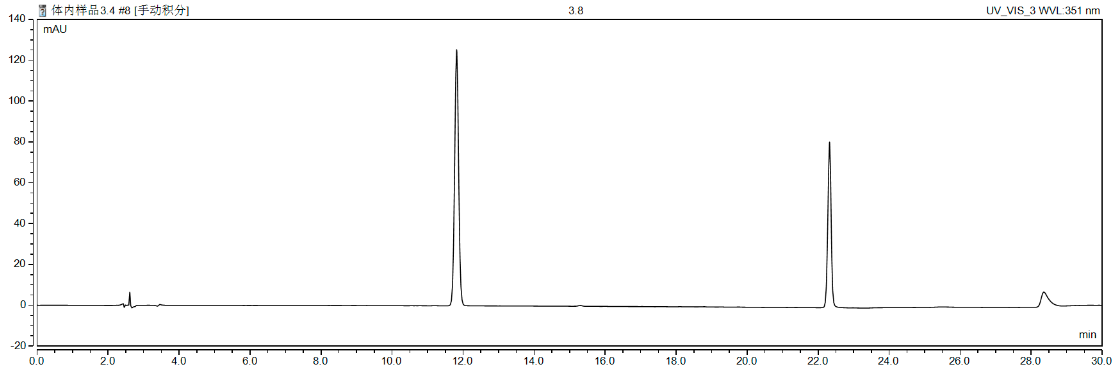

体内样品3.4 #8 [手动积分]
3.8
UV_VIS_3 WVL:351 nm
mAU
min

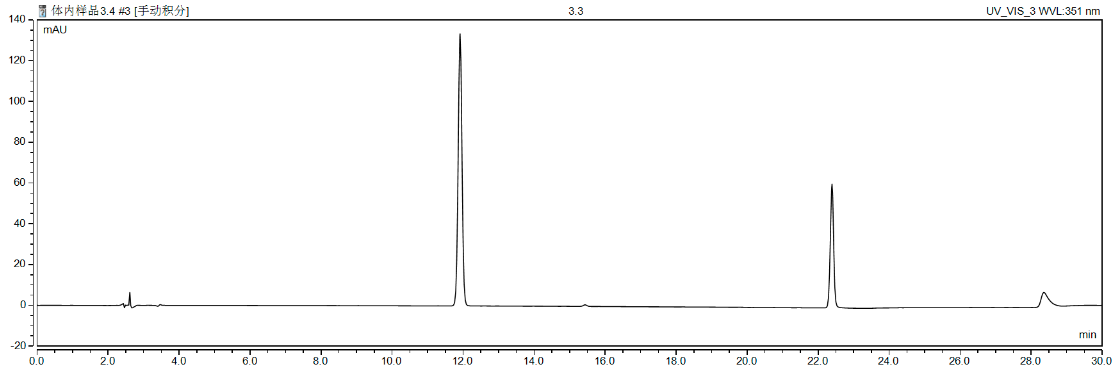
体内样品3.4 #3 [手动积分]
3.3
UV_VIS_3 WVL:351 nm
mAU
140
120
100
80
60
40
20
0
-20
min
0.0
2.0
4.0
6.0
8.0
10.0
12.0
14.0
16.0
18.0
20.0
22.0
24.0
26.0
28.0
30.0

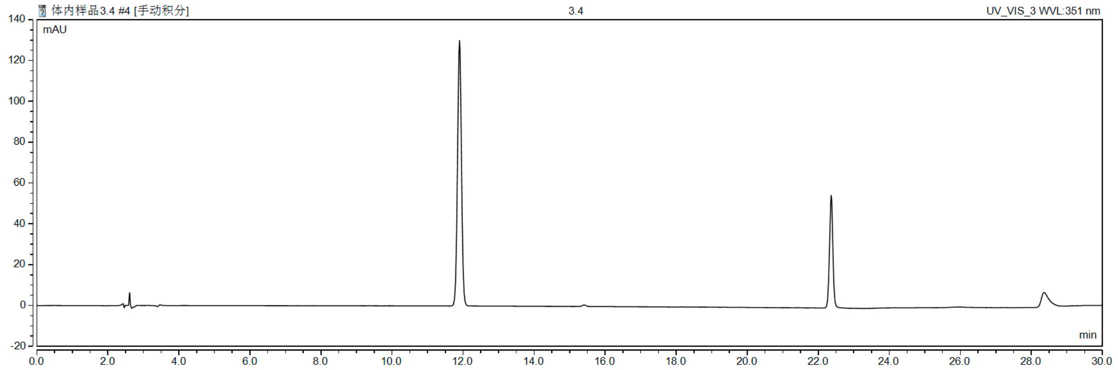
体内样品3.4 #4 [手动积分]
3.4
UV_VIS_3 WVL:351 nm
mAU
140
120
100
80
60
40
20
0
-20
min
0.0
2.0
4.0
6.0
8.0
10.0
12.0
14.0
16.0
18.0
20.0
22.0
24.0
26.0
28.0
30.0

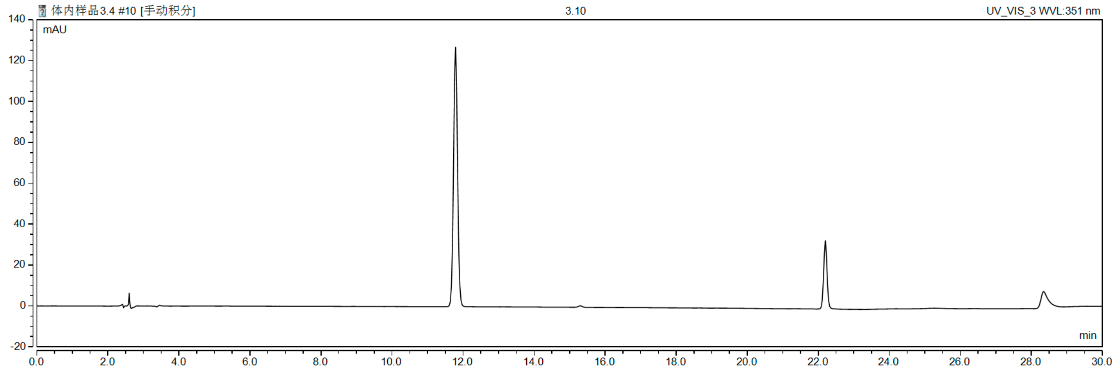
体内样品3.4 #10 [手动积分]
3.10
UV_VIS_3 WVL:351 nm
mAU
140
120
100
80
60
40
20
0
-20
min
0.0
2.0
4.0
6.0
8.0
10.0
12.0
14.0
16.0
18.0
20.0
22.0
24.0
26.0
28.0
30.0

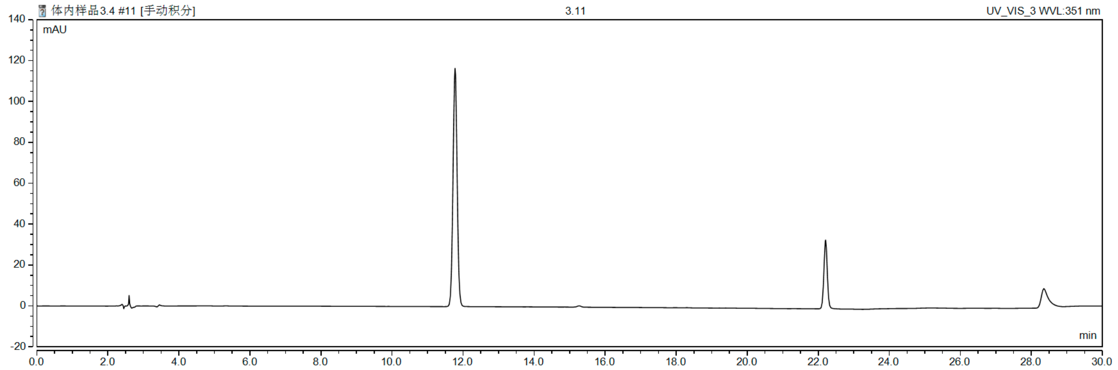
体内样品3.4 #11 [手动积分]
3.11
UV_VIS_3 WVL:351 nm
mAU
140
120
100
80
60
40
20
0
-20
min
0.0
2.0
4.0
6.0
8.0
10.0
12.0
14.0
16.0
18.0
20.0
22.0
24.0
26.0
28.0
30.0

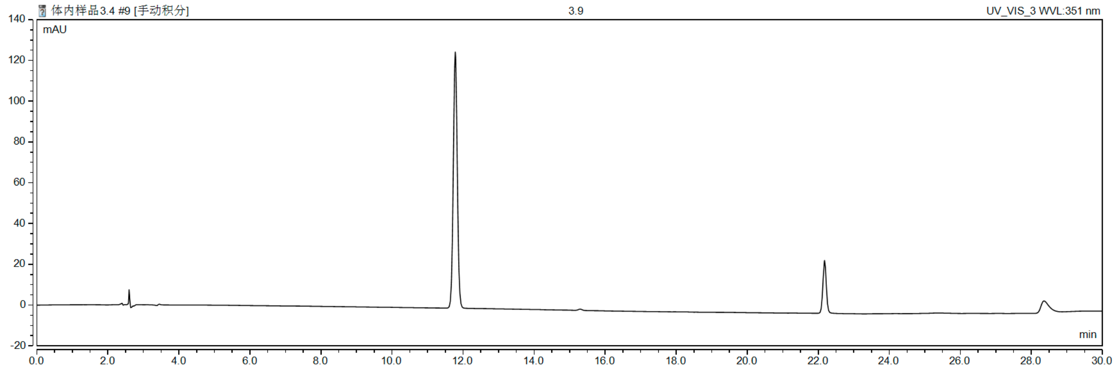
体内样品3.4 #9 [手动积分]
3.9
UV_VIS_3 WVL:351 nm
mAU
min

four

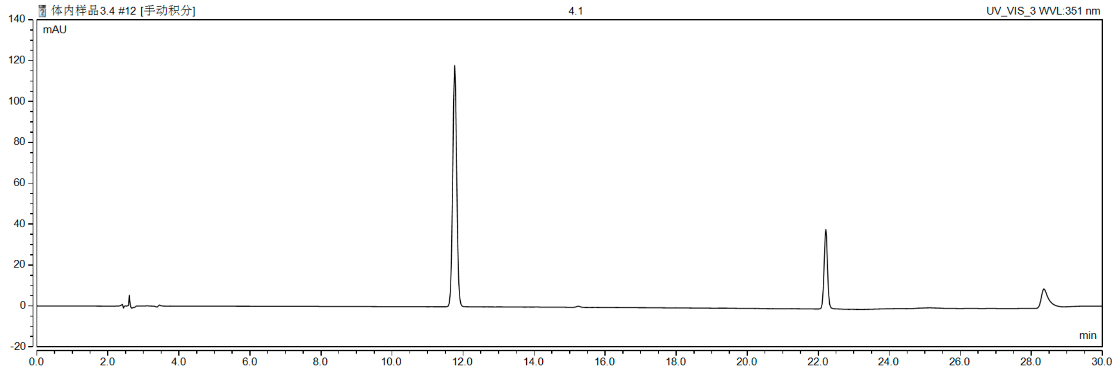
体内样品3.4 #12 [手动积分]
4.1
UV_VIS_3 WVL:351 nm
mAU
min

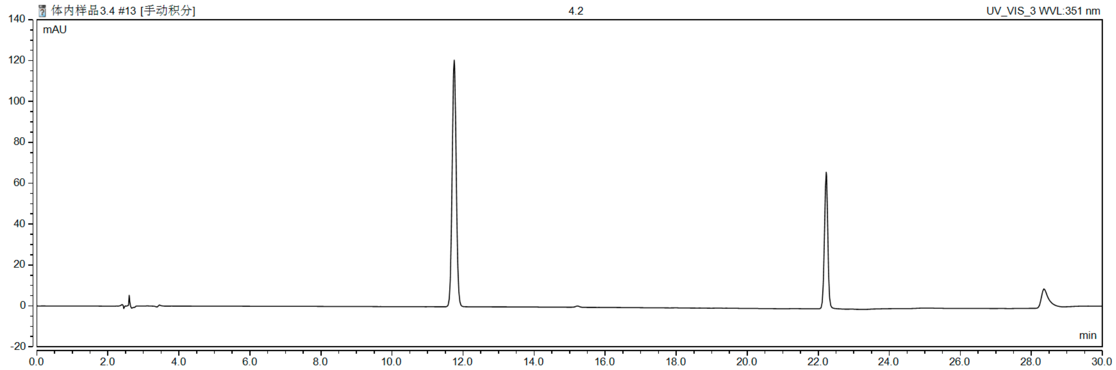
体内样品3.4 #13 [手动积分]
4.2
UV_VIS_3 WVL:351 nm
mAU
min

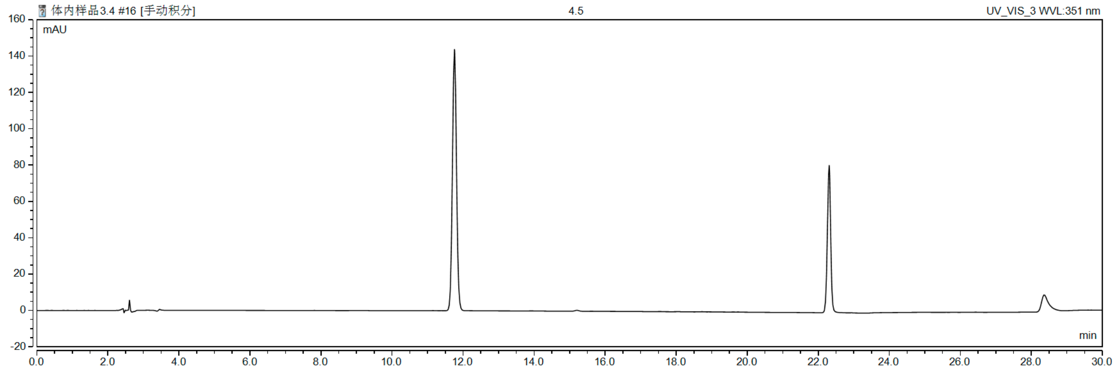
体内样品3.4 #16 [手动积分]
4.5
UV_VIS_3 WVL:351 nm
mAU
min

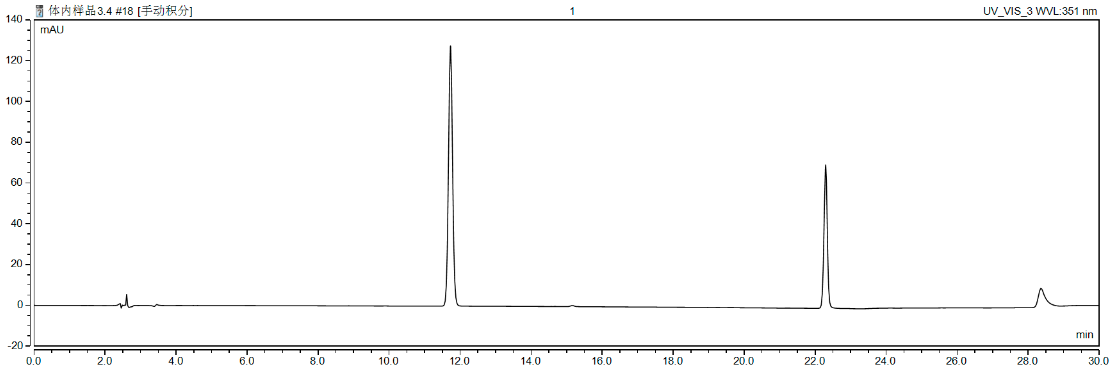
体内样品3.4 #18 [手动积分]
1
UV_VIS_3 WVL:351 nm
mAU
min

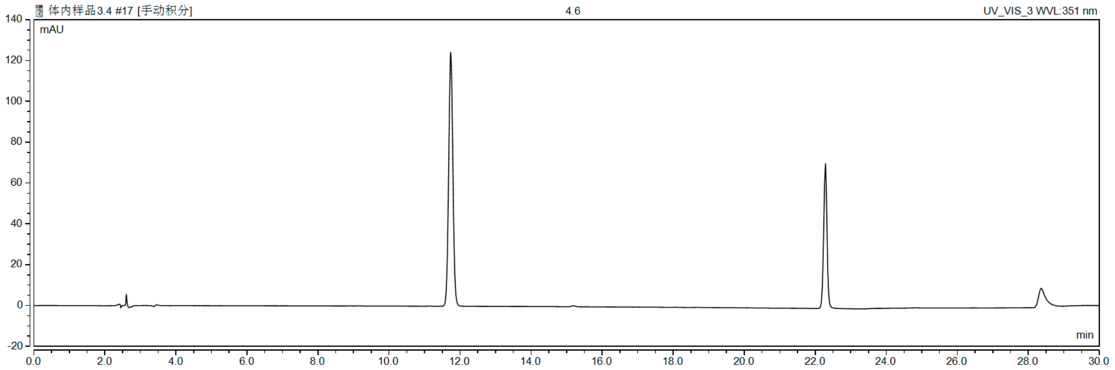
体内样品3.4 #17 [手动积分]
4.6
UV_VIS_3 WVL:351 nm
mAU
min

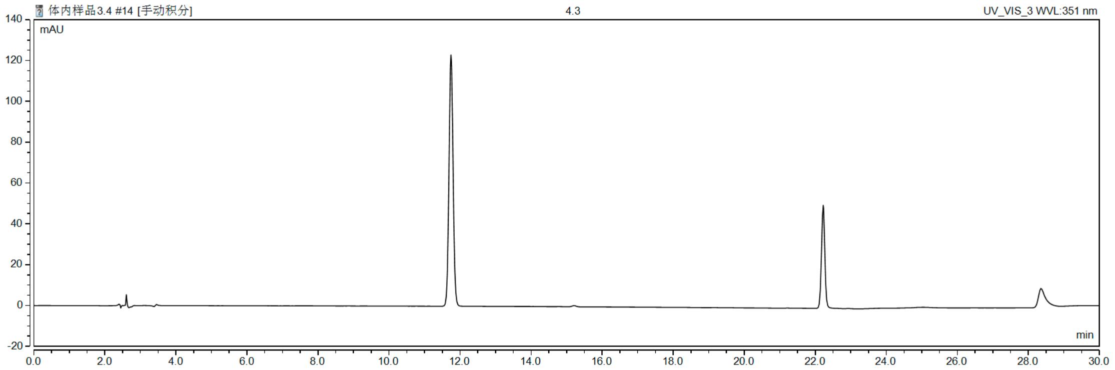
体内样品3.4 #14 [手动积分]
4.3
UV_VIS_3 WVL:351 nm
mAU
min

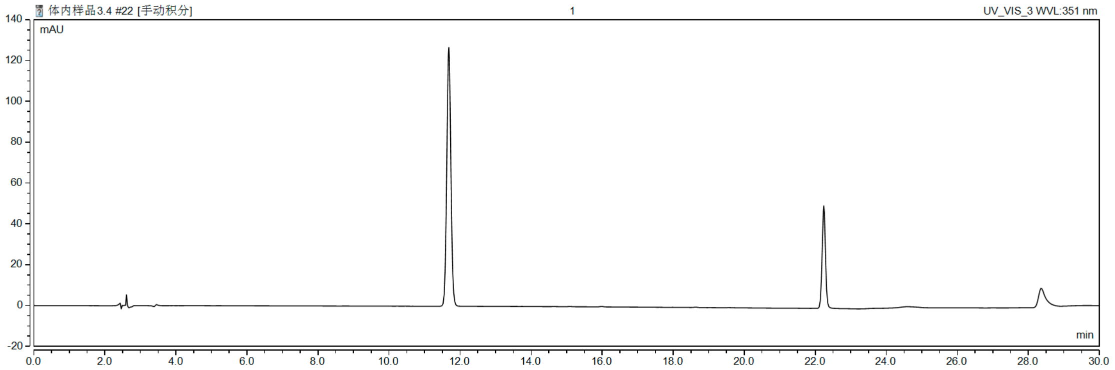
体内样品3.4 #22 [手动积分]
1
UV_VIS_3 WVL:351 nm
mAU
min

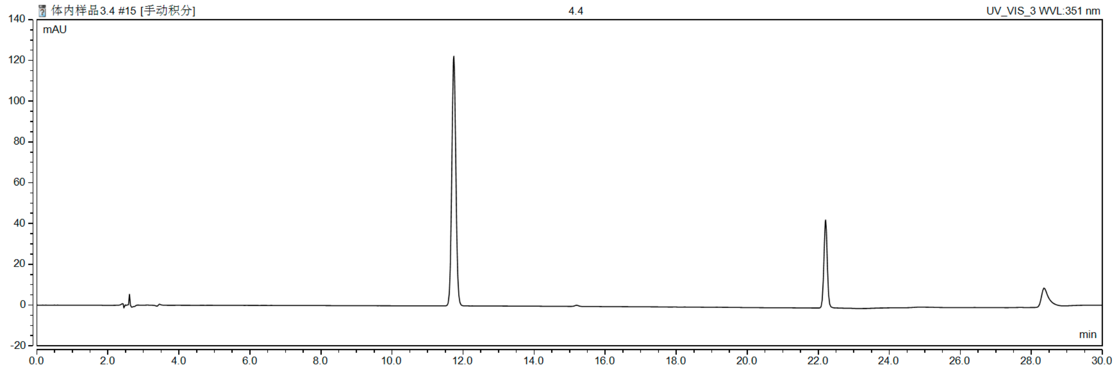
体内样品3.4 #15 [手动积分]
4.4
UV_VIS_3 WVL:351 nm
mAU
min

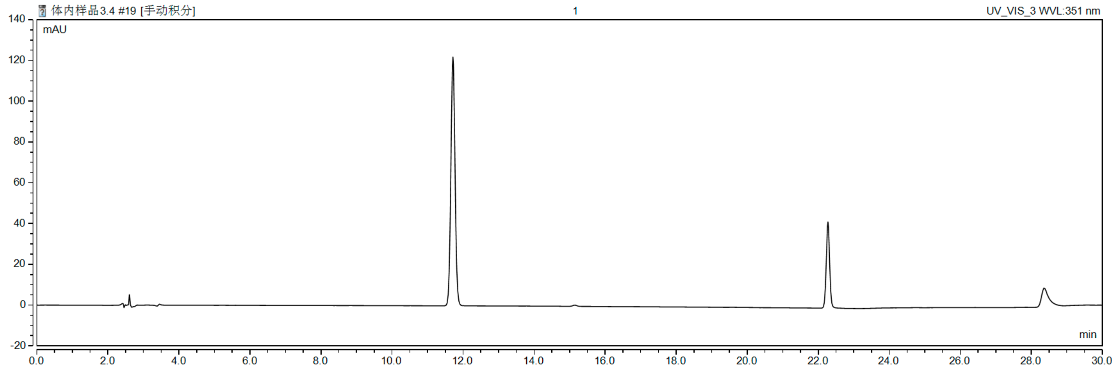
体内样品3.4 #19 [手动积分]
1
UV_VIS_3 WVL:351 nm
mAU
min

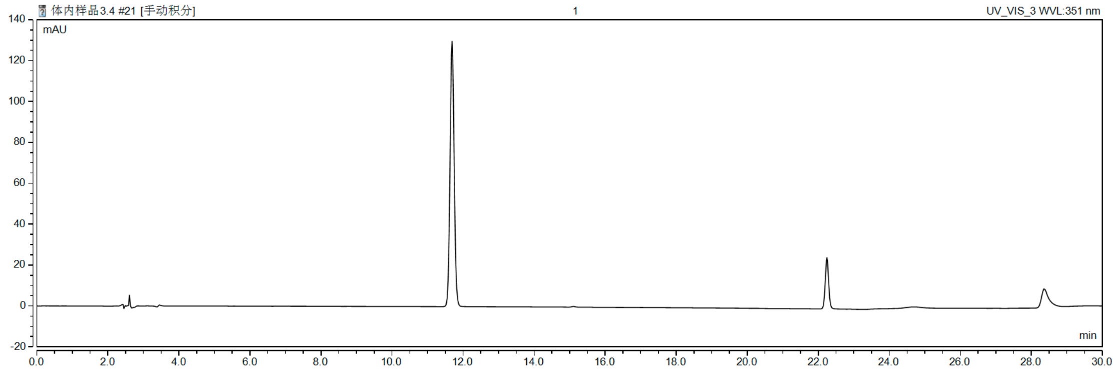
体内样品3.4 #21 [手动积分]
1
UV_VIS_3 WVL:351 nm
mAU
min

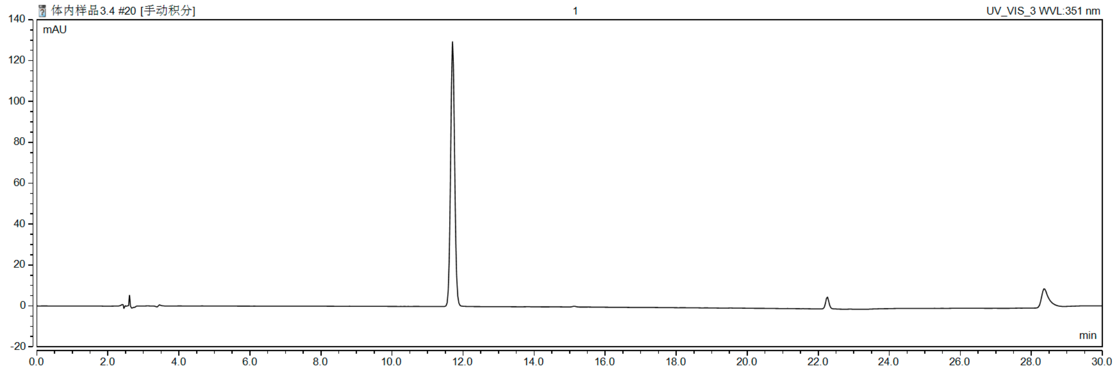
体内样品3.4 #20 [手动积分]
1
UV_VIS_3 WVL:351 nm
mAU
min

five

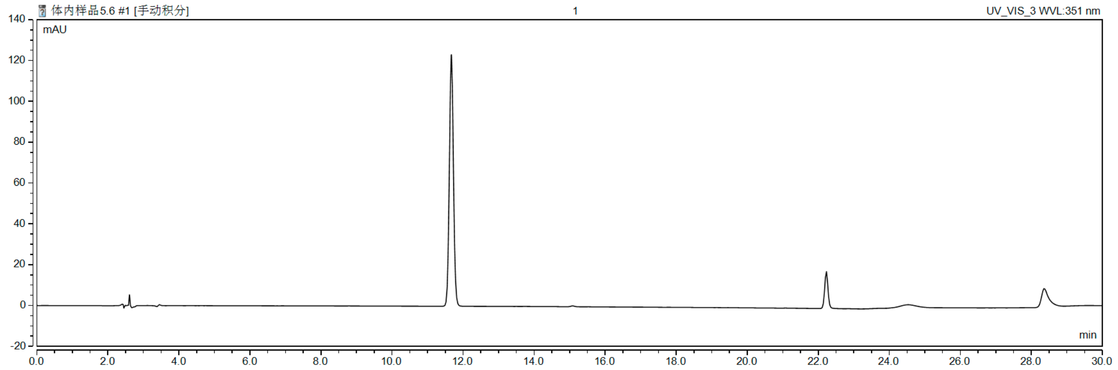
体内样品5.6 #1 [手动积分]
1
UV_VIS_3 WVL:351 nm
mAU
140
120
100
80
60
40
20
0
-20
min
0.0
2.0
4.0
6.0
8.0
10.0
12.0
14.0
16.0
18.0
20.0
22.0
24.0
26.0
28.0
30.0

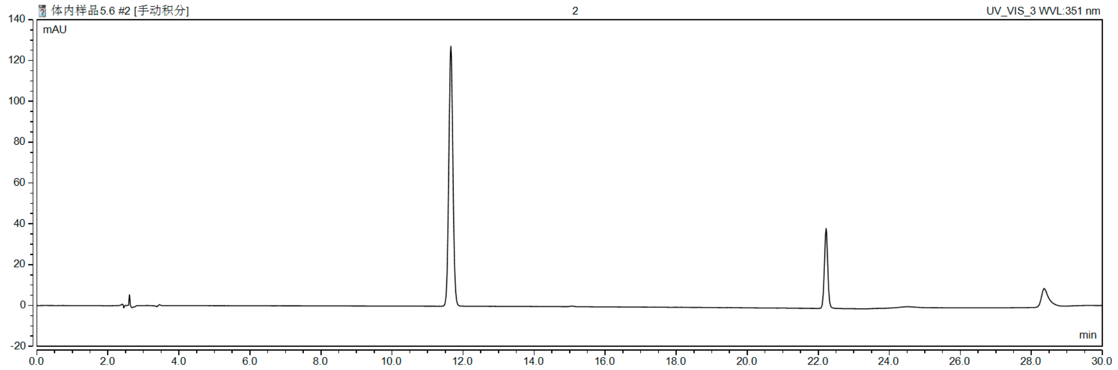
体内样品5.6 #2 [手动积分]
2
UV_VIS_3 WVL:351 nm
mAU
140
120
100
80
60
40
20
0
-20
min
0.0
2.0
4.0
6.0
8.0
10.0
12.0
14.0
16.0
18.0
20.0
22.0
24.0
26.0
28.0
30.0

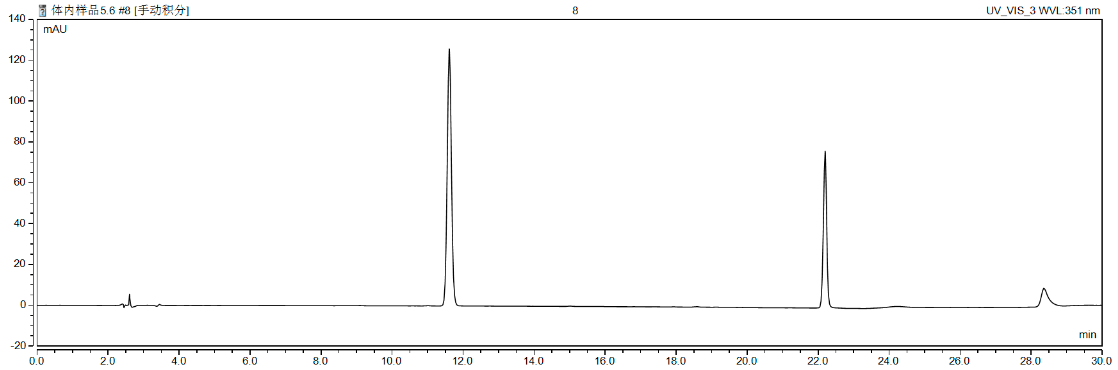
体内样品5.6 #8 [手动积分]
8
UV_VIS_3 WVL:351 nm
mAU
140
120
100
80
60
40
20
0
-20
min
0.0
2.0
4.0
6.0
8.0
10.0
12.0
14.0
16.0
18.0
20.0
22.0
24.0
26.0
28.0
30.0

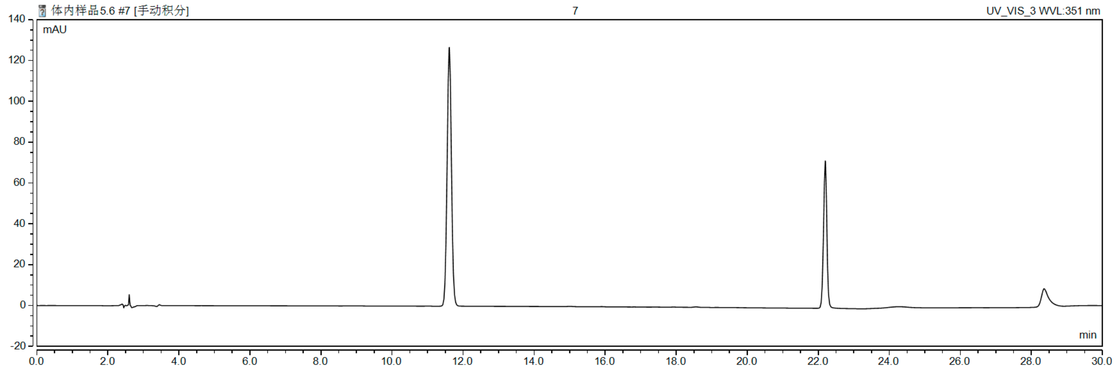
体内样品5.6 #7 [手动积分]
7
UV_VIS_3 WVL:351 nm
mAU
140
120
100
80
60
40
20
0
-20
min
0.0
2.0
4.0
6.0
8.0
10.0
12.0
14.0
16.0
18.0
20.0
22.0
24.0
26.0
28.0
30.0

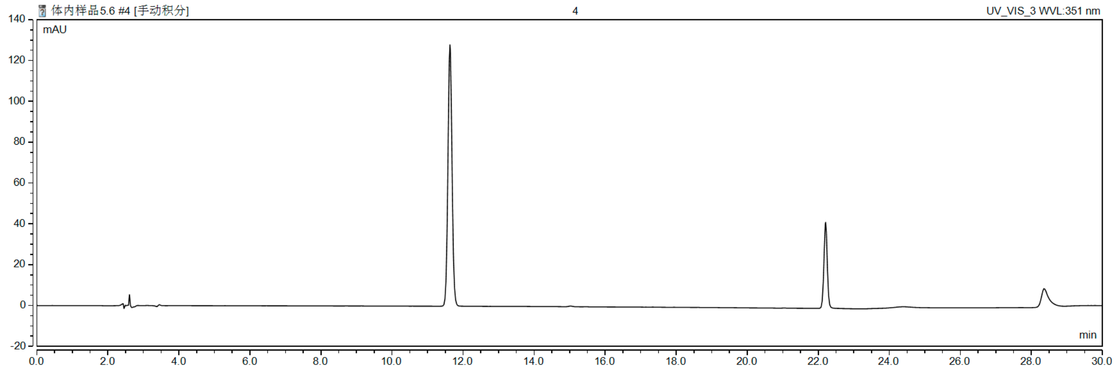
体内样品5.6 #4 [手动积分]
4
UV_VIS_3 WVL:351 nm
mAU
min

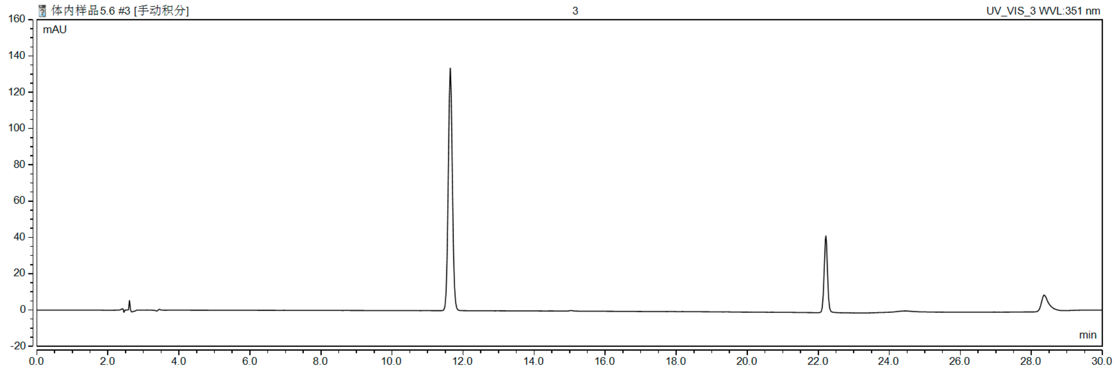
体内样品5.6 #3 [手动积分]
3
UV_VIS_3 WVL:351 nm
mAU
min

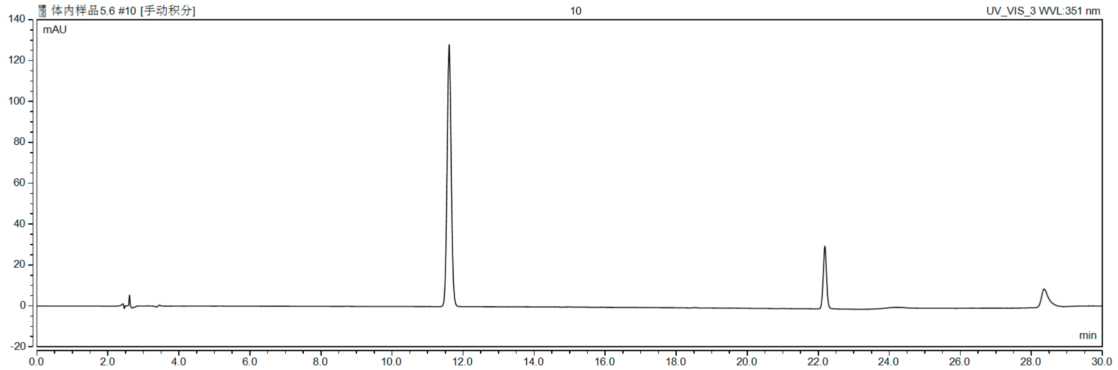
体内样品5.6 #10 [手动积分]
10
UV_VIS_3 WVL:351 nm
mAU
min

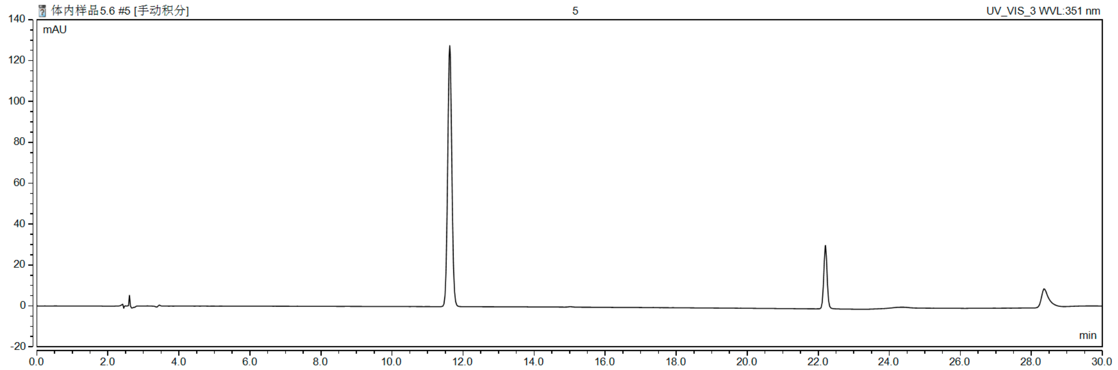
体内样品5.6 #5 [手动积分]
5
UV_VIS_3 WVL:351 nm
mAU
min

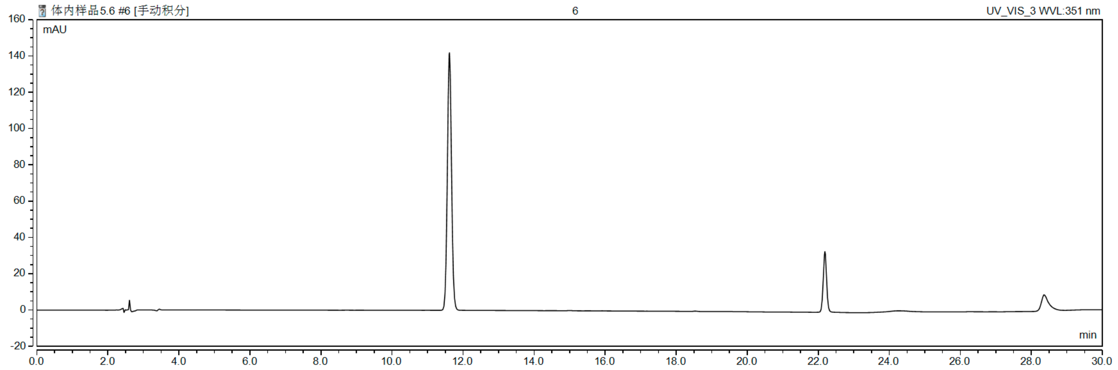
体内样品5.6 #6 [手动积分]
6
UV_VIS_3 WVL:351 nm
mAU
min

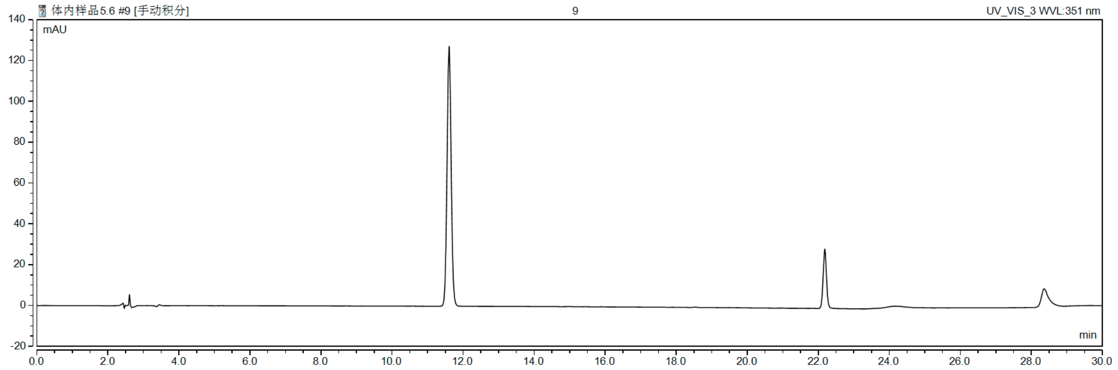
体内样品5.6 #9 [手动积分]
9
UV_VIS_3 WVL:351 nm
mAU
min

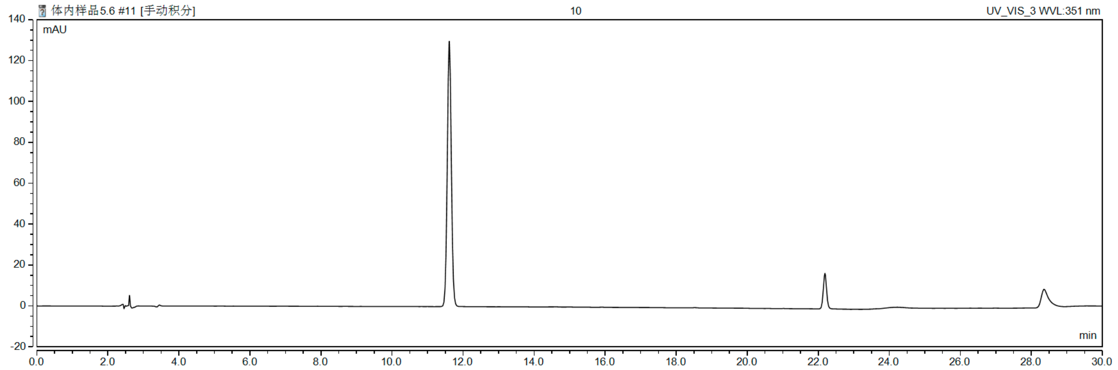
体内样品5.6 #11 [手动积分]
10
UV_VIS_3 WVL:351 nm
mAU
min

six

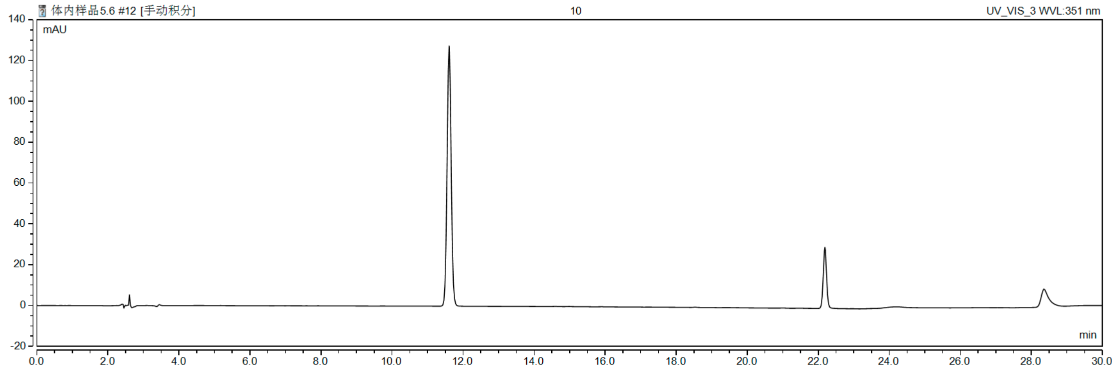
体内样品5.6 #12 [手动积分]
10
UV_VIS_3 WVL:351 nm
mAU
min

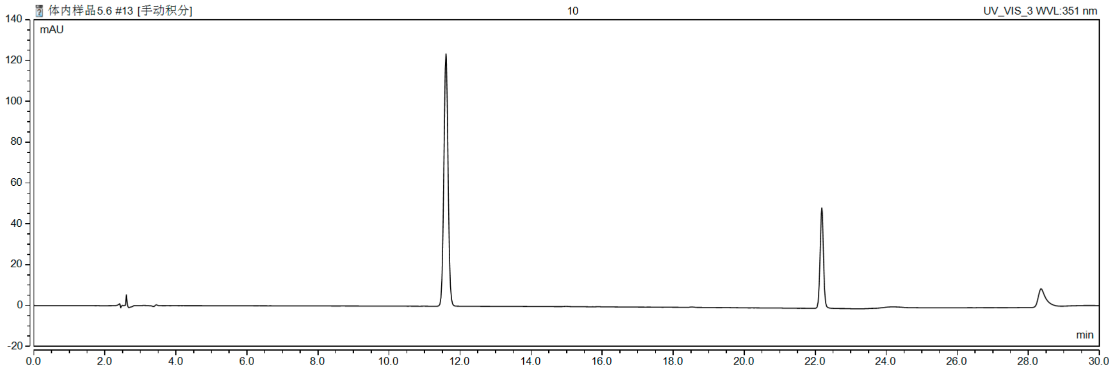
体内样品5.6 #13 [手动积分]
10
UV_VIS_3 WVL:351 nm
mAU
min

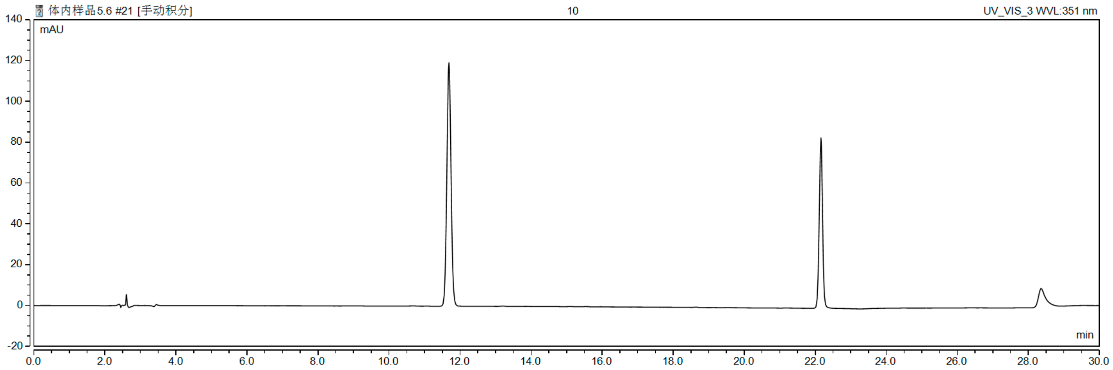
体内样品5.6 #21 [手动积分]
10
UV_VIS_3 WVL:351 nm
mAU
min

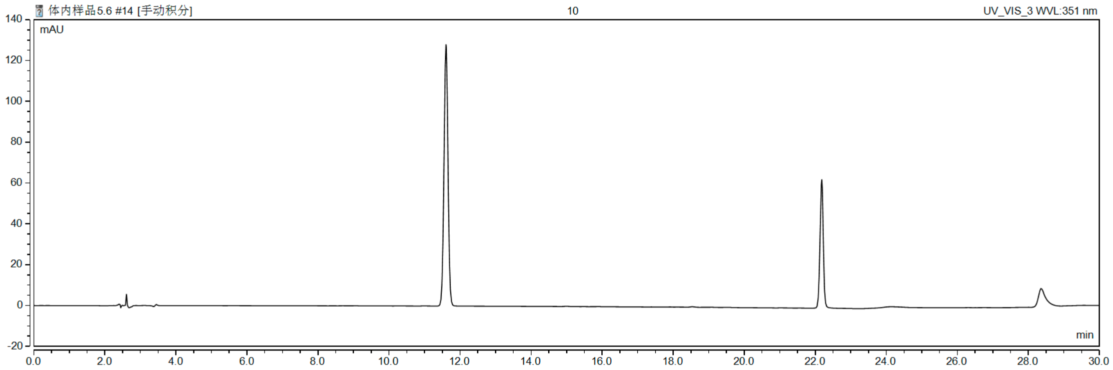
体内样品5.6 #14 [手动积分]
10
UV_VIS_3 WVL:351 nm
mAU
min

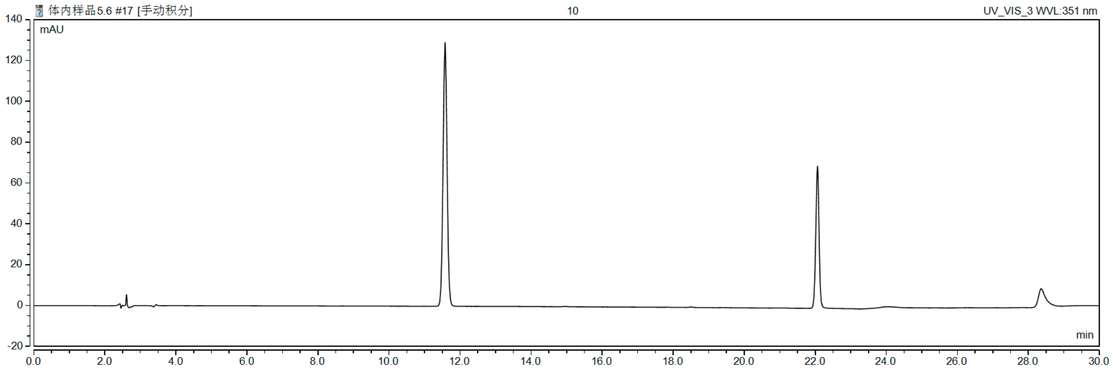
体内样品5.6 #17 [手动积分]
10
UV_VIS_3 WVL:351 nm
mAU
min

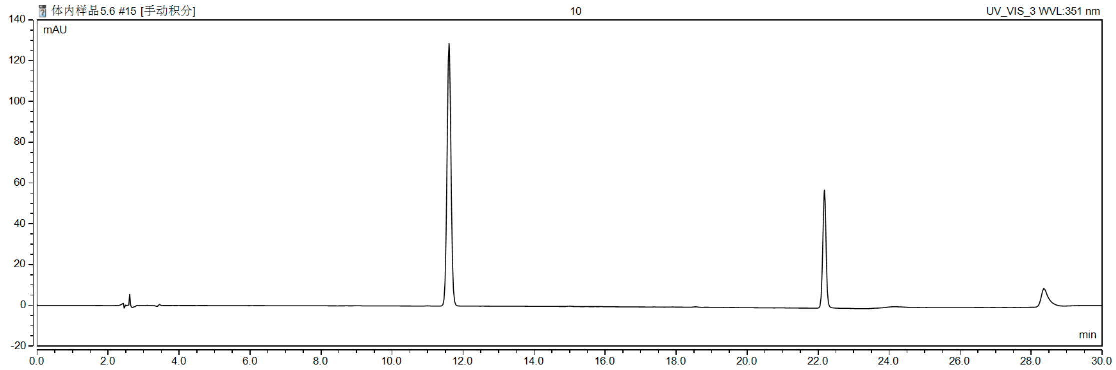
体内样品5.6 #15 [手动积分]
10
UV_VIS_3 WVL:351 nm
mAU
min

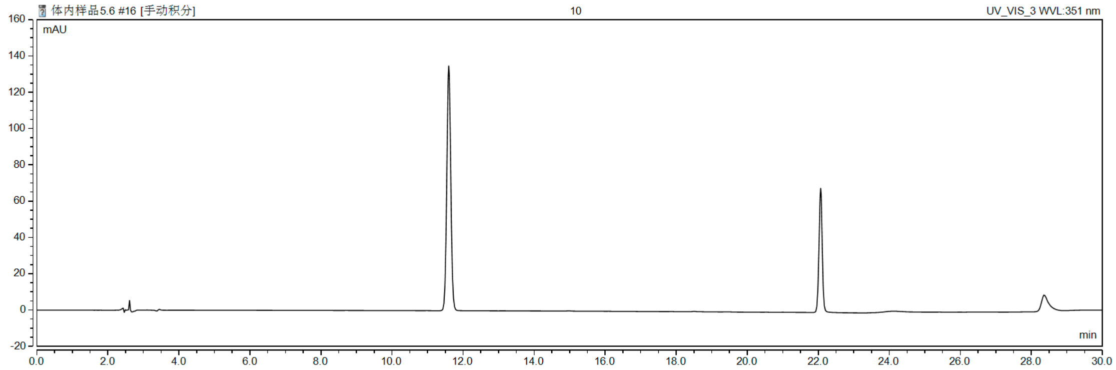
体内样品5.6 #16 [手动积分]
10
UV_VIS_3 WVL:351 nm
mAU
min

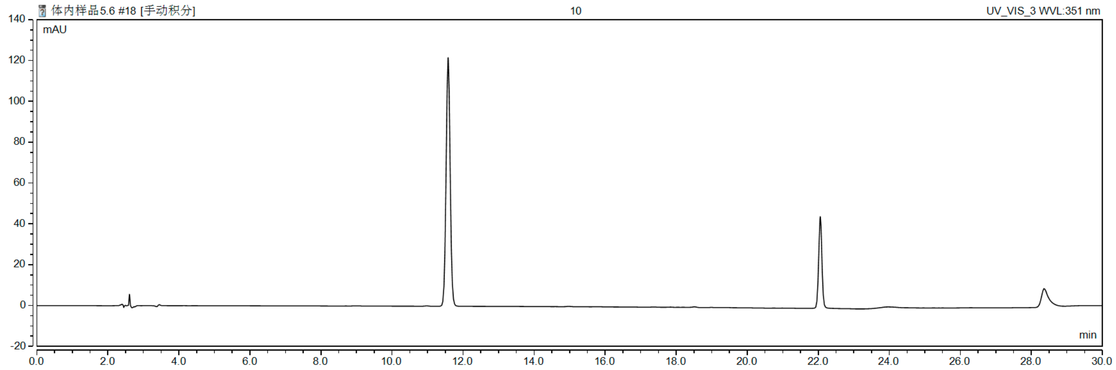
体内样品5.6 #18 [手动积分]
10
UV_VIS_3 WVL:351 nm
mAU
min

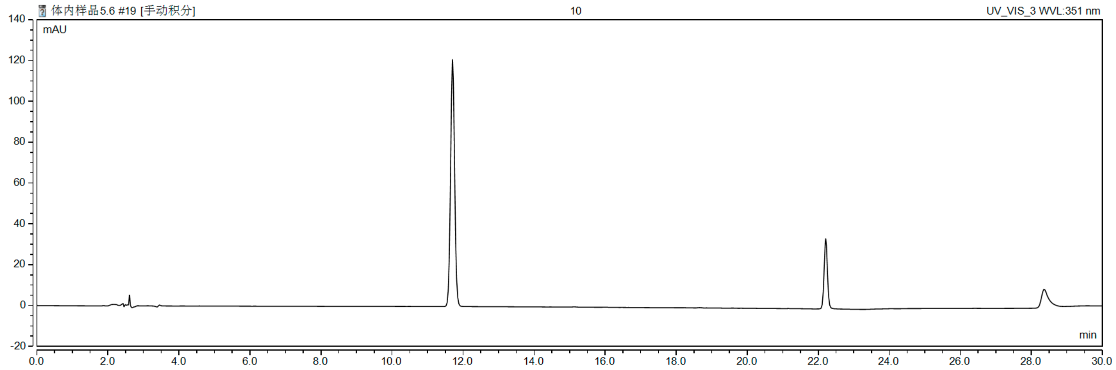
体内样品5.6 #19 [手动积分]
10
UV_VIS_3 WVL:351 nm
mAU
min

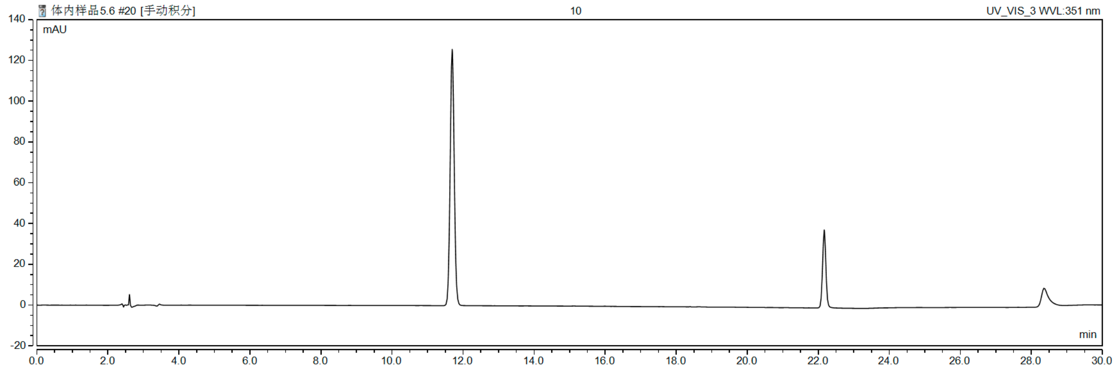
体内样品5.6 #20 [手动积分]
10
UV_VIS_3 WVL:351 nm
mAU
min

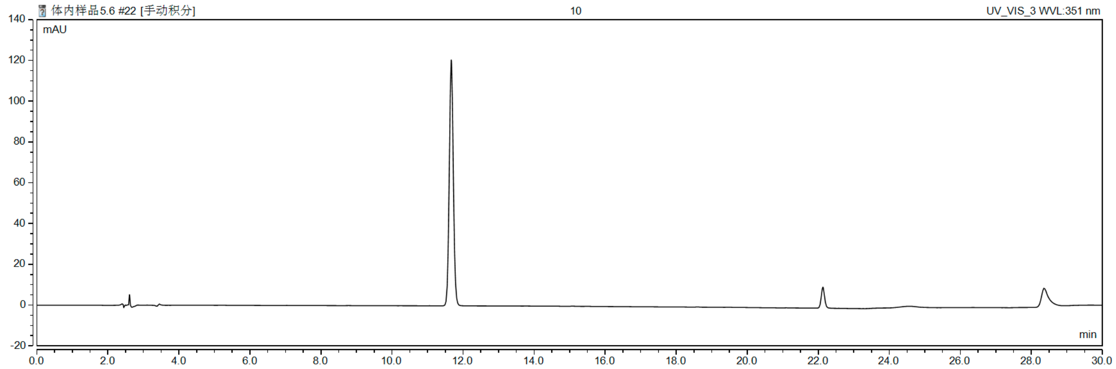
体内样品5.6 #22 [手动积分]
10
UV_VIS_3 WVL:351 nm
mAU
min
